# Outer membrane phospholipase A’s roles in *Helicobacter pylori* acid adaptation

**DOI:** 10.1186/s13099-017-0184-y

**Published:** 2017-06-12

**Authors:** Hilde S. Vollan, Tone Tannæs, Dominique A. Caugant, Gert Vriend, Geir Bukholm

**Affiliations:** 1 Department of Clinical Molecular Biology (EpiGen), Division of Medicine, Akershus University Hospital and University of Oslo, PO box 28, 1478 Lørenskog, Norway; 2 Norwegian Institute of Public Health, Box 4404, Nydalen, 0403 Oslo, Norway; 3 Department of Community Medicine and Global Health, Faculty of Medicine, University of Oslo, P.O. Box 1130, Blindern, 0318 Oslo, Norway; 4 CMBI, Radboudumc, 6525 GA Nijmegen, The Netherlands; 5 Norwegian University of Life Sciences, PO Box 5003, 1430 Ås, Norway

**Keywords:** *Helicobacter pylori*, Acid tolerance, Multifunctional OMPLA, Urea pathway

## Abstract

**Background:**

The pH of the human gastric mucosa varies around 2.5 so that only bacteria with strong acidic stress tolerance can colonize it. The ulcer causing *Helicobacter pylori* thrives in the gastric mucosa. We analyse the roles of the key outer membrane protein OMPLA in its roles in acid tolerance.

**Results:**

The homology model of *Helicobacter pylori* outer membrane phospholipase A (OMPLA) reveals a twelve stranded β-barrel with a pore that allows molecules to pass with a diameter up to 4 Å. Structure based multiple sequence alignments revealed the functional roles of many amino acids, and led to the suggestion that OMPLA has multiple functions. Besides its role as phospholipase it lets urea enter and ammonium exit the periplasm. Combined with an extensive literature study, our work leads to a comprehensive model for *H. pylori*’s acid tolerance. This model is based on the conversion of urea into ammonium, and it includes multiple roles for OMPLA and involves two hitherto little studied membrane channels in the OMPLA operon.

**Conclusion:**

The three-dimensional model of OMPLA predicts a transmembrane pore that can aid *H. pylori*’s acid tolerance through urea influx and ammonium efflux. After urea passes through OMPLA into the periplasm, it passes through the pH-gated inner membrane channel UreI into the cytoplasm where urease hydrolyses it into NH_3_ and CO_2_. Most of the NH_3_ becomes NH_4_^+^ that is likely to need an inner membrane channel to reach the periplasm. Two genes that are co-regulated with OMPLA in gastric Helicobacter operons could aid this transport. The NH_4_^+^ that might leave the cell through the OMPLA pore has been implicated in *H. pylor’s* pathogenesis.

**Electronic supplementary material:**

The online version of this article (doi:10.1186/s13099-017-0184-y) contains supplementary material, which is available to authorized users.

## Background

### *H. pylori* survives in the human gastric mucosa

Most bacterial proteins require a distinct pH to function correctly, and that optimal pH is usually much higher than the gastric pH of around 2.5 [1]. Some bacteria, however, thrive in the acidic gastric mucosa [1]. Bacterial cell walls and membranes generally are leaky [2], which would, in the stomach, rapidly lower their cytosolic pH if they had no effective acid stress relieve mechanism [1]. The fact that the stomach pH fluctuates depending on food and liquid intake [3] puts additional constraints on this acid stress relieve mechanism.

The *Helicobacter* genus comprises a versatile group of species that is found in different hosts, usually colonizing the intestine, liver, or stomach [4]. They can be divided into gastric- and enterohepatic-*Helicobacters*, with different morphology and genetic diverse lineages [5]. *H. pylori* is the most prevalent species observed in the human gastric mucosa [6].

When *H. pylori* enters the acidic gastric lumen, it migrates towards the epithelial surface. The pH in the epithelial surface is approximately 5, but it can fluctuate from pH 6 to pH 1 [1]. When *H. pylori* is in an acidic environment [7] pH-dependent transcription factors like Fur, NikR, and ArsRS [8] trigger overexpression of proteins involved in motility and ammonia production. The increased expression of motility genes allows the bacteria to move away from the acidity [7], while the ammonia will protect the bacterium by buffering the influx of protons [1].

### *H. pylori* OMPLA implicated in colonization

Outer membrane (OM) proteins often are multifunctional and involved in maintaining the membrane integrity [9]. They form the first line of defence by detecting possible attacks on the membrane for which purpose many OM proteins function as signal transducers [9].

An intact *H. pylori* outer membrane phospholipase A (OMPLA) has been suggested necessary for colonization of the human gastric ventricle [10, 11]. Only *H. pylori* with intact OMPLA will survive in acidic environments in vitro, whereas phase variants with truncated OMPLA can survive at neutral pH. At neutral pH the bacterial variants with intact OMPLA, have an altered lipid composition. The switch between intact and truncated OMPLA is explained by a phase-variable DNA slippage in the homopolymeric tract of the OMPLA gene, *pldA* [12]. Phase-variable proteins are often implicated in roles involving bacteria—environment interactions [13]. At pH 5 only *H. pylori* with intact OMPLA (OMPLA_ON_-variant) is selected in vitro, even though the enzymatic activity at pH 5 is turned off because the pH optimum for enzymatic activity is 7.

OMPLA is found in several Gram-negative species [14], and it has been suggested that OMPLA activity is triggered by diverse events, such as temperature shift or heat shock, toxin release, or membrane instability. OMPLA activity in *Escherichia coli* is related to loss of membrane integrity [9]. *E. coli* OMPLA is activated under a wide variety of conditions (e.g. membrane perturbation) by calcium-induced dimerization [15]. OMPLA may be activated under various stress conditions in different species a where phospholipase activity contributes to cell wall degradation [9, 11], but *H. pylori* OMPLA is not likely to be activated by acidity since the bacterium needs an intact OM to survive the harsh conditions in the gastric mucosa.

### The *H. pylori* acid defence mechanism hypothesis

**Fig. 1:**
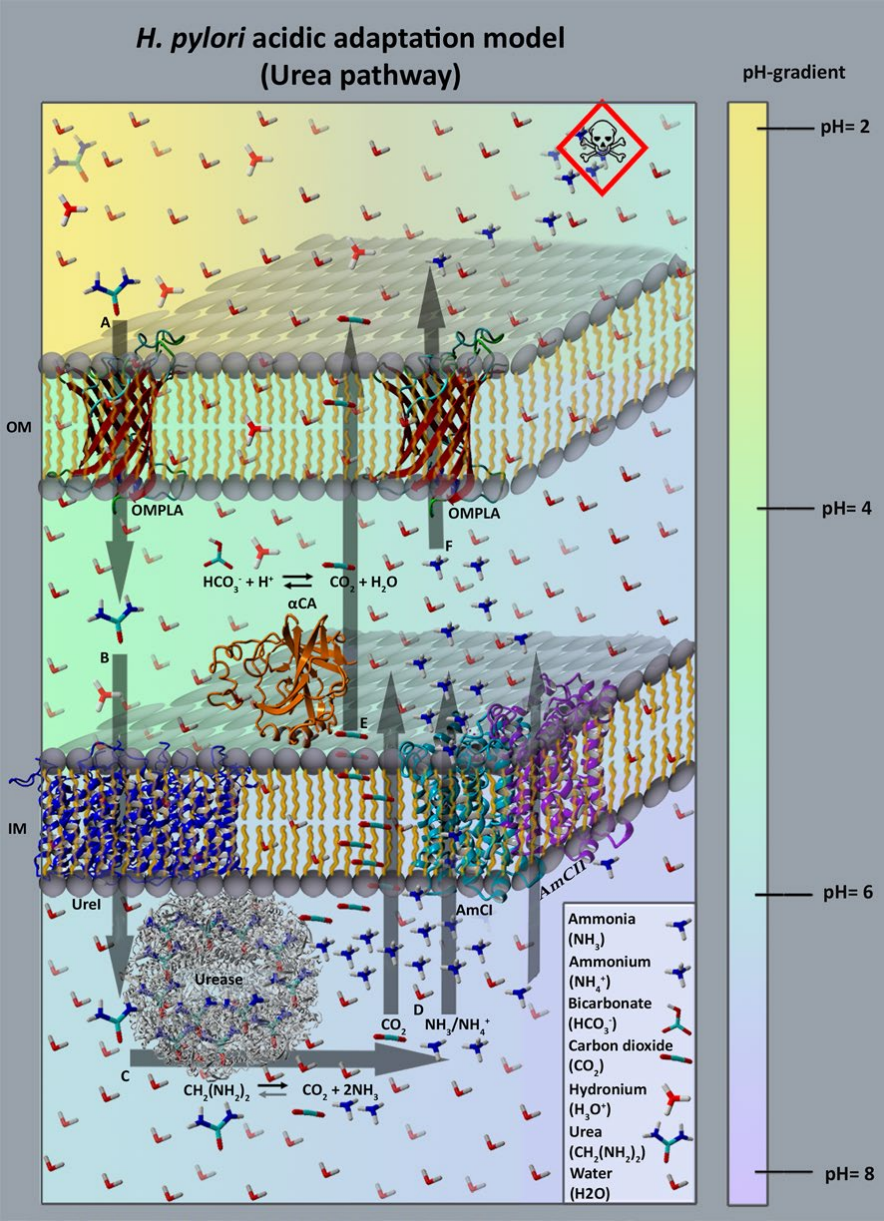
*H. pylori* acid adaptation model. This figure schematically depicts our hypothesis on how *H. pylori* uses urea in acid protection. *A* Urea passes through OMPLA into the periplasm simply driven by the diffusion gradient. *B* Urea then diffuses through the proton-dependent inner membrane (IM) urea channel UreI to the cytoplasm. *C* Urease converts urea into NH_3_ and CO_2_. *D* These molecules diffuse into the periplasm. CO_2_ diffuses through the membrane simply following the gradient. At pH around 6 NH_3_ will largely be converted to NH_4_^+^ that passes through IM channels. A very small portion of NH_3_ will continuously remain and a small fraction of this NH_3_ will diffuse through the IM. *E* Most of the CO_2_ diffuses out of the cell, but some is converted by the periplasmic enzyme α-carbonic anhydrase (αCA) into bicarbonate. This bicarbonate forms a buffer that helps maintaining the periplasmic pH around 6.1. A sudden enlarged influx of protons will be buffered by this bicarbonate. *F* NH_4_^+^ leaves the cell mainly through OMPLA. The toxicity sign represents the damage caused by NH_4_^+^ in the gastric mucosa. The pH gradient is indicated in the *colour bar* at the *right-hand side*. PDB files used in this illustration: 3UX4 [15] (UreI; *blue*), 4XFW [16] (αCA; *orange*) and 1E9Y [17] (Urease; *grey*). The *red structure* shown twice in the outer membrane, is a homology model (*H. pylori* OMPLA model based on the 1QD5 [14] PDB template). The *purple and turquoise* proteins embedded in the *right-front corner* of the IM are COG0733 transporter models. We suggest these transporters can diffuse NH_4_^+^ (and NH_3_?) across the IM (we call them ammonium channels I and II, AmCI and AmCII; these models are based on the 4US3 [18] PDB template structure). This figure does not reflect real-life concentrations

Intact OMPLA is required for survival of *H. pylori* in acidic environments despite the fact that OMPLA is enzymatically inactive at low pH [10, 12]. Combining these findings with experimental work on five novel isogenic pairs of OMPLA variants, and in silico analyses of OMPLA sequence and structure data augmented with a literature study, we arrive at a hypothesis for a functional mechanism for this acid protection. This hypothesis is depicted in Fig. 1, and a detailed overview of the six steps in Fig. 1 can be found in Additional file 1. All details of this model and the corroborating evidence are presented in the remainder of this article.

## Results

Our bioinformatics work, combined with an extensive literature study, and backed up by experimental work on variants with non-functional OMPLA is summarized in Fig. 1. The remainder of this article discusses all aspects of this model.

### Survival of *H. pylori* OMPLA variants in acidic environment

Sequencing the variants revealed classical phase variants displaying shorter OMPLA sequences in euBL, euAP, and euBF; see Table 1. Two variants (euBZ and euBB; see Table 1) have missense mutations resulting in a non-functional OMPLA. Under prolonged growth in acidic pH conditions, only those with an ON phase and/or without missense mutations survive [12]. Thus, OMPLA_OFF_ is non-functional OMPLA due to either a truncated OMPLA or a missense mutation. All five OFF variants grow at neutral pH, lacking OMPLA activity and they do not survive at all at low pH.

**Table 1:** Characteristic of *H. pylori* colony variants showing altered phospholipase A activity

*H. pylori* isolates		OMPLA length (amino acids)	Enzyme activity	Truncated OMPLA or missense mutation?	Sequence ID (GenBank ID)
euBL (98019)	OMPLA_ON_	355	Yes	–	AFR51755.1
	OMPLA_OFF_	263	No	Truncated OMPLA	N/A
euAP (29A)	OMPLA_ON_	355	Yes	–	AFR51733.1
	OMPLA_OFF_	265	No	Truncated OMPLA	N/A
euBF (5A)	OMPLA_ON_	355	Yes	–	AFR51749.1
	OMPLA_OFF_	263	No	Truncated OMPLA	N/A
euBZ (9B)	OMPLA_ON_	355	Yes	–	AFR51769.1
	OMPLA_OFF_	355	No	Missense mutation (Pro157Ser)	N/A
euBB (53A)	OMPLA_ON_	355	Yes	–	AFR51745.1
	OMPLA_OFF_	355	No	Missense mutation (Ser235Arg)	N/A

The isolates used in this article are the same as previously published [14]

The existence of these variants further emphasizes the importance of OMPLA for survival at low pH.

### *H. pylori* OMPLA 3D structure model

**Fig. 2:**
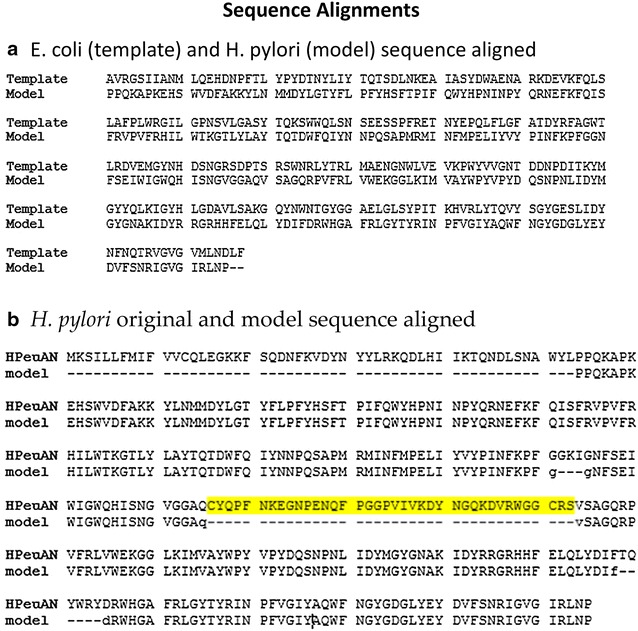
Sequence alignments. The sequence alignment used in constructing a homology model of *H. pylori* OMPLA from *E. coli* OMPLA (1QD5 PDB template file). **a** The model (*H. pylori* OMPLA) and template (*E. coli* OMPLA) sequences extracted from the MSA (see Additional file 2). **b** The original HPeuAN sequence (GenBank Accession ID: AFR51731) aligned to the model sequence extracted from the MSA. The *highlighted yellow* fragment is the not-modelled 38 residues long insert

A total of 3084 sequences were included in the OMPLA Multiple Sequence Alignment (MSA; see Additional file 2). Extracting *E. coli* and *H. pylori* OMPLA sequences from the MSA yielded the sequence alignment shown in Fig. 2a. This alignment was used to construct the OMPLA 3D structure model. The extracted *H. pylori* OMPLA sequence is compared to the original unaligned sequence (HPeuAN, GenBank Accession ID: AFR51731) in Fig. 2b. *H. pylori* OMPLA has a 38-residues long insert (the so-called ‘not-modelled insert’ that is highlighted yellow in Fig. 2b). This region consists of a sequence not found in the template *E. coli* sequence, and Blastp only finds acid-tolerant gastric *Helicobacteraceae* containing this sequence element. The YASARA/WHAT IF twinset predicts that this insert constitutes a loop without any regular secondary structure.

**Fig. 3:**
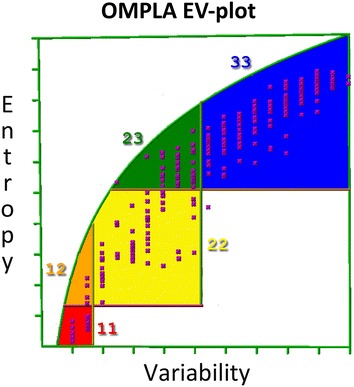
EV-plot for 3084 OMPLA sequences. *Each box* represents a class of entropy-variability and is coloured as described in the “Methods” section

**Fig. 4:**
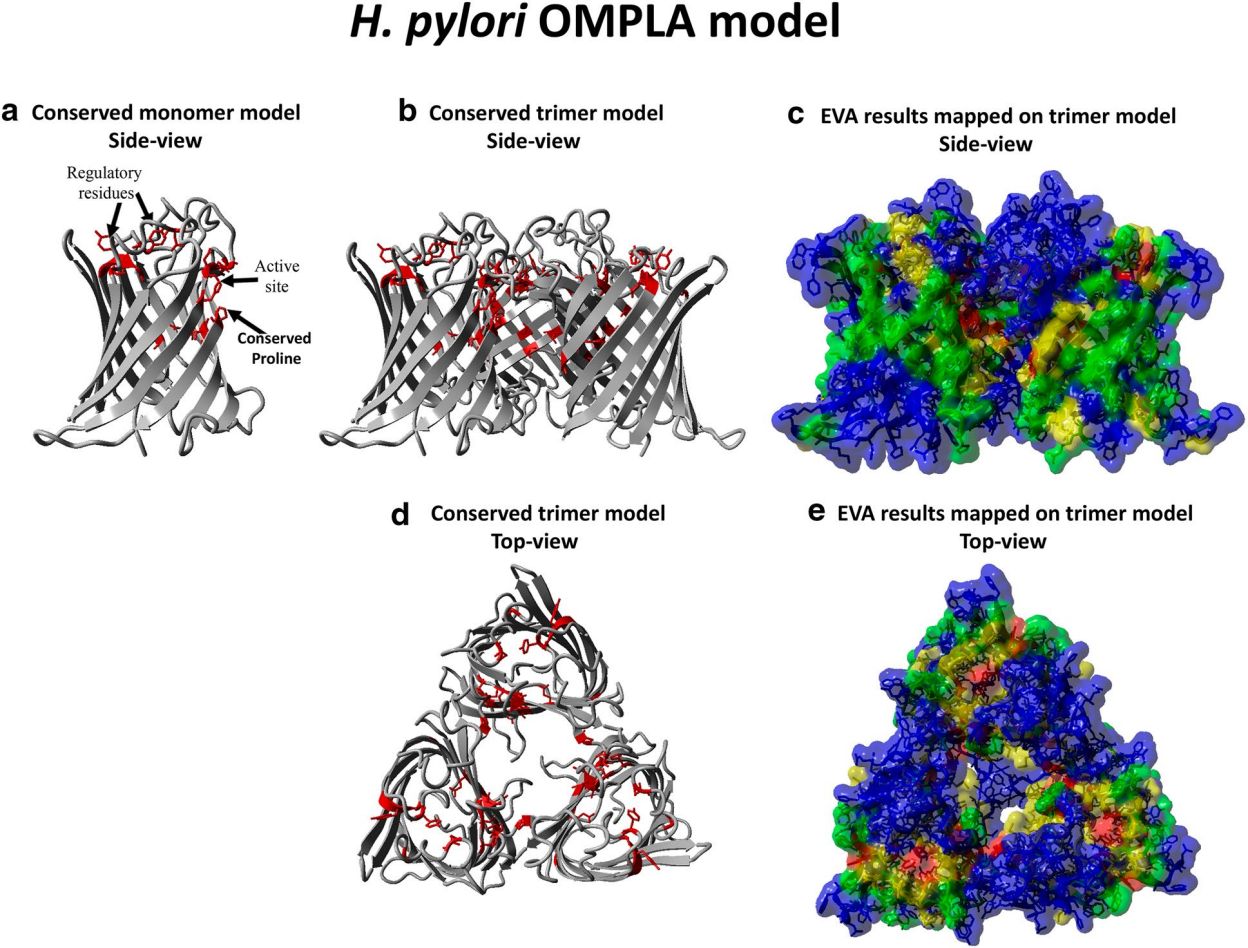
EVA and *H. pylori* OMPLA model. **a** The most conserved residues are shown as *red stick model* on a OMPLA monomer model (*side view*) in which the active site is indicated. **b** As **a**, but now a OMPLA trimer is used. **c** EVA results mapped on the *H. pylori* OMPLA all-atom trimer (*side view*) coloured by EV-plot section. **d** As **b**, but *top view*. **e** As **c**, but *top view*. The *blue* residues that cover the trimeric hole are periplasmic loop residues (*coloured grey* in **d**)

The MSA was used for entropy-variability analyses (EVA), and Fig. 3 shows the resulting EV plot for OMPLA sequences, which were mapped onto the *H. pylori* OMPLA model structure in Fig. 4. There are highly variable (blue) residues located in the exterior loops, while the most conserved (red) residues are located in the trimer interface, the active site, and the calcium-binding site, while a few are located in extracellular loops. The highly conserved extracellular loop-residues, that must have an important function for OMPLA, include two tyrosine residues: Y233 and Y240 in loops 4 and 6, respectively (see Fig. 4a, b).

**Fig. 5:**
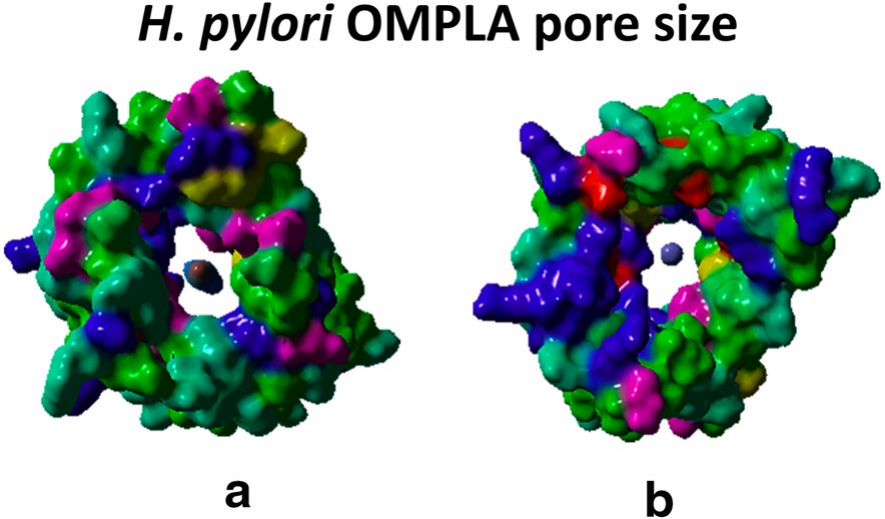
OMPLA pore. Both OMPLA and substrates are visualized with their molecular surface using YASARA/WHAT IF twinset. Urea and NH_4_^+^ (and perhaps NH_3_) easily pass through the OMPLA polar pore. Positively charged residues (Arg, Lys, and His) are *coloured blue*, negatively charged residues (Glue, and Asp) are *coloured red*, polar residues (Asn, and Gln) are *coloured purple*, hydrophobic residues (Gly, Ala, Val, Leu, and Pro) are *coloured grass green*, alcoholic residues (Thr and Ser) are *coloured light green*, aromatic residues (Phe, Tyr, and Trp) are *coloured light blue-green*, and sulphur containing residues (Cys, Met). **a** *Top-view* w/urea. ** b** *Bottom-view* w/ammonium

The resulting 3D structure model of *H. pylori* OMPLA shows that molecules like urea and ammonium could easily pass through the *H. pylori* OMPLA pore which has a diameter of at least 4 Å, which is much wider than the 1.5 Å diameter of the UreI (see Fig. 5; see Additional file 3) pore that is known to let urea pass into the cytosol (see Additional file 1).

**Fig. 6:**
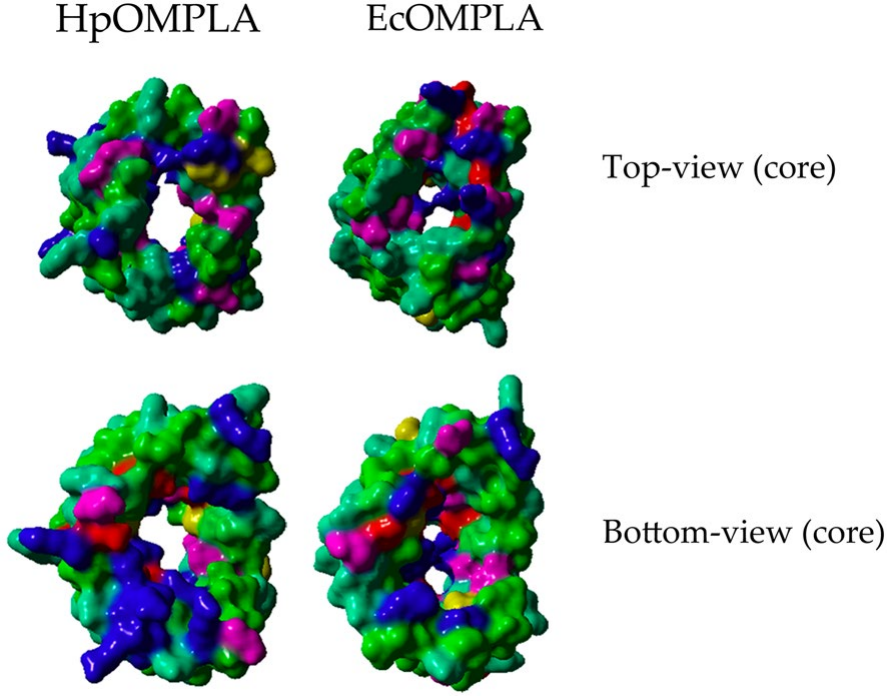
Comparing *H. pylori* and *E. coli* core OMPLA residues. Structures are visualized in YASARA/WHAT IF twinset with molecular surfaces. Residues are *coloured yellow*. Residues are coloured according to amino acid properties as described in Fig. 5

The differences in pore size and amino acid composition between the *H. pylori* OMPLA model and *E. coli* OMPLA structure are visualized in Fig. 6.

### Sequence similarities in acid tolerant species of the *Helicobacteraceae* family

**Fig. 7:**
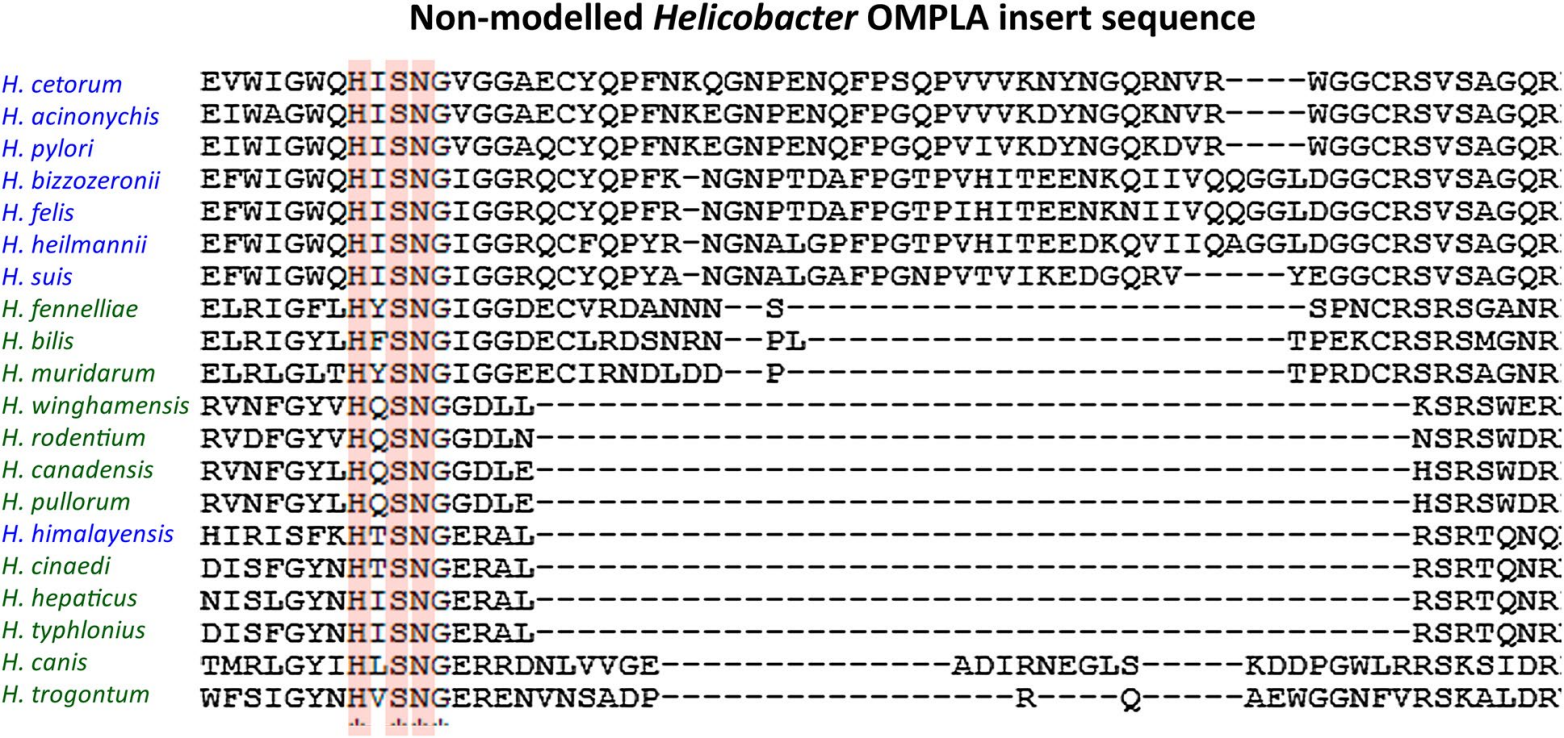
Not-modelled insert sequence. The alignment of the inserted region among the *Helicobacter* species is shown (see Table 2 for more information). The residues *highlighted in red* constitute the catalytic triad in OMPLA. Gastric species names are in *blue*; enterohepatic species names are in *green*. The *H. himalayensis* YS1 genome lacks urease genes, indicating lack of the urease pathway

The not-modelled sequence highlighted in Fig. 2b is found in nearly all gastric *Helicobacter* species, but is lacking in enterohepatic *Helicobacter* species (see Table 2; Fig. 7). *H. pylori*’s OMPLA shares the highest sequence identity with other gastric *Helicobacter* OMPLAs, and we will explore how they differ from enterohepatic *Helicobacters* (see Fig. 7).

**Table 2:** Non-modelled OMPLA insert in *Helicobacter* sequences

Species	Representative strain	Classification	Predicted loop insert
*H. acinonychis*	Sheeba	Gastric	Insert
*H. bizzozeronii*	CIII-1	Gastric	Insert
*H. cetorum*	MIT 99-5656	Gastric	Insert
*H. heilmannii*	ASB1.4	Gastric	Insert
*H. pylori*	ATCC 43504	Gastric	Insert
*H. suis*	HS5 (partially sequenced)	Gastric	Insert
*H. felis*	ATCC 49179	Gastric	Insert
*H. mustelae*	12198	Gastric	NA
*H. himalayensis*	YS1	Gastric	No insert
*H. bilis*	ATCC 43879	Enterohepatic	Short insert
*H. canadensis*	MIT 98-5491	Enterohepatic	No insert
*H. canis*	NCTC 12740	Enterohepatic	No insert
*H. cinaedi*	CCUG 18818 = ATCC BAA-847	Enterohepatic	No insert
*H. fennelliae*	MRY 12-0050	Enterohepatic	Short insert
*H. hepaticus*	ATCC 51449	Enterohepatic	No insert
*H. muridarum*	ST-1	Enterohepatic	Short insert
*H. pullorum*	MIT 98-5489	Enterohepatic	No insert
*H. rodentium*	ATCC 700285	Enterohepatic	No insert
*H. trogontum*	ATCC 700114	Enterohepatic	No insert
*H. typhlonicus*	MIT 97-6810	Enterohepatic	No insert
*H. winghamensis*	ATCC BAA-430	Enterohepatic	No insert

This table lists *Helicobacter* species, strain, classification (gastric vs enterohepatic) and whether the OMPLA sequences contain the predicted loop. The “Predicted loop insert”—column lists those species that have a long, unique insert that could not be modelled, but predicted to be a longer loop. They are typically found in gastric bacteria. The term “short insert” indicates shorter loops, likely to have a different function based on their residue composition. See Additional file 4 for the sequence alignment file

### In silico *pldA* operon prediction

**Fig. 8:**
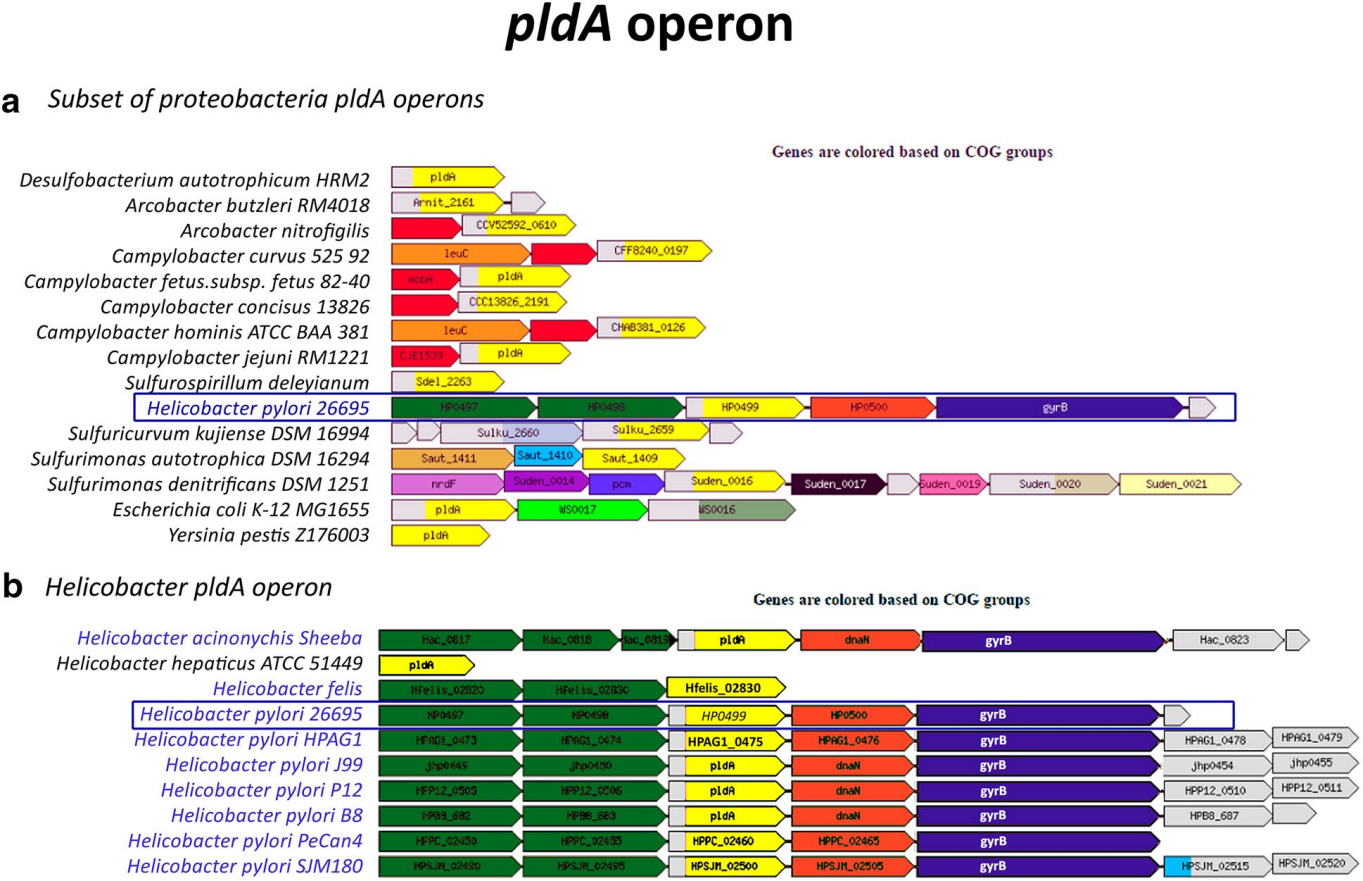
*pldA* operon prediction. Operon prediction for clusters of orthologous groups (COGs) COG2829 (OMPLA; or *pldA* on gene-level) using ProOpDB where **a** illustrates a subset of proteobacteria, including *H. pylori*, *E. coli* and *Yersinia pestis* and **b** illustrates the *Helicobacters* found in the ProOpDB. Gastric *Helicobacter* bacteria are *highlighted in blue*. *Each colour* represents a COG, except *grey* which indicate genes with an unknown COG. *Yellow* represents *pldA* (COG2829); Na^+^-dependent AmCI and AmCII (COG0377) *coloured green*; *dnaN* (COG0592) *coloured orange* and *gyrB* (COG0187) *coloured purple*

Figure 8 shows how the OMPLA gene, *pldA,* is organized in gastric *Helicobacters* compared to a series of other bacteria. Gastric *Helicobacter* species have a common operon organization in which *pldA* lies downstream of two channels that belong to the COG0733 family that is also known as ‘Na^+^-dependent transporters (channels) of the SNF family’ (AmCI and AmCII). According to ProOpDB *pldA* is not part of any operon in enterohepatic *H. hepaticus* (see Fig. 8b). In Table 3 we explore the differences between the gastric and enterohepatic *Helicobacter* gene organizations. It is clearly seen that the AmCI and AmCII channel are found only in the *pldA* operon of gastric *Helicobacter* species, which strongly suggests a role in pH management.

**Table 3:** Operon prediction

Species	Representative strain	Host	Classification	Upstream	Downstream
*H. acinonychis*	Sheeba	Cheetah	Gastric	AmCI and AmCII	*dnaN*, *gyrB*, 2 hypothetical genes
*H. cetorum*	MIT 99-5656	Dolphin	Gastric	AmCI* and AmCII	*dnaN*, *gyrB*
*H. pylori*	ATCC 43504	Human	Gastric	AmCI and AmCII	*dnaN*, *gyrB*, 2 hypothetical genes
*H. bizzozeronii*	CIII-1	Human	Gastric	AmCI and AmCII	No
*H. himalayensis*	YS1	Marmota himalayana	Gastric	No	No
*H. suis*	HS5	Swine	Gastric	AmCI and AmCII	No
*H. felis*	ATCC 49179	Cat	Gastric	AmCI and AmCII	No
*H. mustelae*	12198	Ferret	Gastric	No	No
*H. heilmannii*	ASB1.4	Cat	Gastric	AmCI* and AmCII	No
*H. bilis*	ATCC 43879	Human	Enterohepatic	No	No
*H. canadensis*	MIT 98-5491	Human	Enterohepatic	No	No
*H. canis*	NCTC 12740	Human	Enterohepatic	No	*pseH*
*H. cinaedi*	CCUG 18818 = ATCC BAA-847	Human	Enterohepatic	No	*metE*
*H. fennelliae*	MRY 12-0050	Human	Enterohepatic	Hypothetical	SAM-dependent MTase genes
*H. hepaticus*	ATCC 51449	Mouse	Enterohepatic	No	*metE*, and gene encoding NADPH
*H. muridarum*	ST-1	Rat	Enterohepatic	No	ABC transporter gene
*H. pullorum*	MIT 98-5489	Human	Enterohepatic	No	No
*H. rodentium*	ATCC 700285	Mouse	Enterohepatic	No	No
*H. trogontum*	ATCC 700114	Rat	Enterohepatic	No	No
*H. typhlonicus*	MIT 97-6810	Mouse	Enterohepatic	No	No
*H. winghamensis*	ATCC BAA-430	Human	Enterohepatic	No	No

Operon prediction comparing gastric and enterohepatic genes upstream/downstream from *pldA. Helicobacter* species. Operons are predicted by Fgenesb software (Softberry Inc., Mount Kisco, NY, US). COG0377 corresponds to the “Na^+^-dependent transporters (channels) of the SNF family” (this includes AmCI and AmCII). The COG0377 are phase variable genes

* Truncated proteins

### 3D modelling of IM channels

**Fig. 9:**
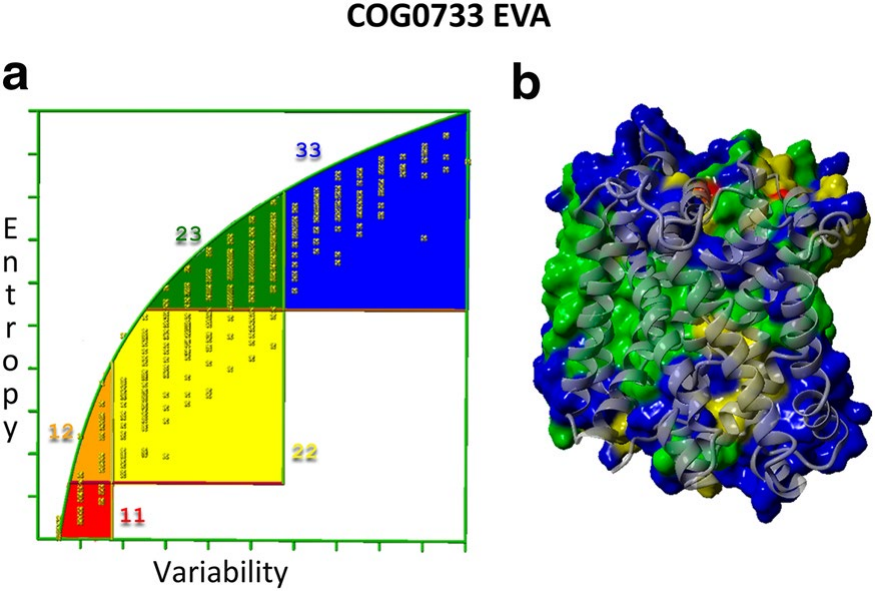
EVA for 4444 COG0733 sequences. EV-plot to the *left* with the residues mapped onto the MhsT structure to the *right* (4US3 PDB template file). *Each box* represents a degree of entropy-variability and is coloured as described in “Methods” section.** a** EV-plot.** b** EVA mapped onto MhsT

The model depicted in Fig. 1 includes two channels to allow NH_3_/NH_4_^+^ to pass from the cytosol to the periplasm. We constructed 3D models for AmCI and AmCII as described for OMPLA, with the same methodology that was used for the analyses of porins [16]. AmCI and AmCII share a pairwise sequence identity of 31–34% to the template, and ~50% to each other. The results are shown in Fig. 9. Visual inspection of these models reveals that AmCI has a more polar pore and thus might be the channel for NH_4_^+^, while AmCII pore is more hydrophobic and thus seems more suitable for letting NH_3_ diffuse to the periplasm. It is also not yet clear if the activity of either of these two channels is a function of either the cytosolic or the periplasmic pH, and we do not (yet) know if AmCI and/or AmCII are sodium symporter or antiporters, or perhaps they even are simple channels and not transporters. Most well characterized sodium or sodium and chlorine dependent transporters have much longer sequences than AmCI and AmCII, suggesting that AmCI and AmCII might miss the co-transporter related domains. Further experiments are needed to answer all these questions, but both the operon structure and the 3D models do not disagree with the idea that AmCI and AmCII help ammonia/ammonium travel from the cytosol to the periplasm.

## Discussion

The *H. pylori* acid regulation pathway has been well studied and often debated (see Additional file 1 for further details) [1], but all currently available functional models still require unknown OM components for urea influx and ammonium efflux. Our in silico results indicate that *H. pylori* OMPLA can be an OM urea and ammonium channel, while AmCI and AmCII, that are likely co-regulated with OMPLA, can be involved in ammonium efflux from the cytosol. The hypothesis presented in this paper, depicted in Fig. 1, is mainly based on in silico models combined with current literature findings on the urea pathway and *H. pylori* acid tolerance.

Unlike *E. coli* OMPLA, *H. pylori* OMPLA is continuously breaking down membrane phospholipids to lysophospholipids when cultivated at physiological pH [17]. The optimal pH for OMPLA’s enzymatic activity is around 7.0, and this activity is abolished at pH 5.0 or lower, yet this protein is required for in vitro survival at very low pH [12].

The OMPLA structure is composed of a 12-stranded transmembrane β-barrel with short periplasmic turns and long extracellular loops. This is consistent with the structure of other outer membrane proteins (OMPs; including that of porins, the largest OMP subfamily) that diffuse molecules through the membrane [16]. The *H. pylori* OMPLA barrel is large enough to have pore activity [16]. Figure 5 supports the idea that there is enough space for urea to pass the OM through OMPLA. The pores of the *E. coli* and *H. pylori* OMPLA vary in shape and amino acid composition, as shown in Fig. 6, supporting our hypothesis that they differ in function. We therefore suggest that *H. pylori* OMPLA is a multifunctional protein with one function being a phospholipase and a second function being acid protection.

Standard sequence alignment methods are reliable for closely related sequences, but often fail when diverse sequences-like OMPLAs- are analysed [18, 19]. The alignment of the *H. pylori* OMPLA sequence with the sequence of the *E. coli* OMPLA template structure (1QD5 PDB) is complicated. To get this alignment we collected as much information as possible about homologous OMPLA sequences and generated a MSA using an iterative profile alignment process. Unlike standard sequence alignment methods, iterative profile alignments can use both structure and function information. The *H. pylori* and *E. coli* OMPLA protein sequences are highly different (see Additional file 2) and their sequence identity is just above the threshold for homology modelling [20] when the not-modelled insert (Fig. 2) is not taken into account.

EV analyses the evolutionary footprint left behind in a MSA and identifies regions in the protein in which conservation and variation relate to various aspects of thee protein’s function [16, 19]. The highly variable residues (coloured blue in Fig. 4) observed mainly at the outside facing loops likely are (a-specifically) involved in host evasion [16]. The two highly conserved loop tyrosines (Fig. 4) are located far from the trimer interface, yet they must be functionally important because they are conserved in the MSA.

Most gastric *Helicobacters* have a sequence insert that is lacking in the enterohepatic *Helicobacters* (and other species). Table 2 shows two gastric *Helicobacters* that are very different and that lack this sequence insert: *Helicobacter mustelae* and *Helicobacter himalayensis*. In phylogenetic analyses, *H. mustelae* cluster with enterohepatic *Helicobacters* [21]. The genome from *H. mustelae* type strain 12,198 [22] lacks *pldA,* the gene coding for OMPLA. *H. mustelae* possess a nickel-independent urease, called UreAB2 [23]. In the presence of urea this metalloprotein is by itself already sufficient for the bacteria to survive acid shock. It is activated by ferrous ions in the absence of auxiliary proteins [23]. *H. mustelae* has adapted a different mechanism to survive in the stomach of its host, the ferret. The other gastric species that differs, *H. himalayensis* lacks urease genes, which indicates that it lacks the whole urease pathway. Generally, gastric and enterohepatic *Helicobacter* OMPLAs are quite different; enterohepatic *Helicobacters* lack the insert found in most gastric *Helicobacters*, except the OMPLAs from *H. mustelae* and *H. himalayensis,* that seem to have evolved totally different systems to cope with low pH.

The not-modelled insert is similar to that found in the sequence alignment of a subset of sequences presented by Istvan et al. [11]. This not-modelled insert (see Table 2; Figs. 2, 7 for more details) can be of importance for pH-gating as observed in other proteins such as *H. pylori* UreI [24] and *E. coli* OmpG [25]. pH sensitivity has also been detected in the *E. coli* OmpF constriction loop [26].

We do not know the function of the residues in the predicted extracellular loop (the not-modelled insert highlighted in Fig. 2b), but literature has shown that mutating a charged extracellular loop residue can have deleterious effects on acid survival [27, 28]. Our laboratory results (the missense mutations P157S and S235R, see Additional file 5 for residue numbering) revealed the importance of these two OMPLA residues. Ser235 is located in the not-modelled loop insert. Arginine is a positively charged residue and its introduction will lead to electrostatic changes, perhaps resulting in a functionally closed pore. P157 is one of the highly-conserved residues. It is located at the trimer interface (see Fig. 4). This mutation likely destabilizes the β-barrel structure [16, 29, 30], or might disable acidic protection by disturbing the trimer interface that is required for pore-activity [16]. The conserved cysteines, found in the gastric *Helicobacter* OMPLA lie in this extracellular loop. Cys residues are seldom found in OMPs [31] and they are seldom found facing the surface; however, conserved cysteines have a wide range of functions and are usually of great importance to the protein [32–34]. Although protein stabilization would be a likely function for this conserved disulfide bond, further laboratory work is needed to confirm its function. Inspection of the model suggests that Y240 stabilizes the extracellular loops; especially the interactions between the first and third extracellular loop. We have previously predicted that all porins function as a trimer [16], and provided evidence that OMPLA forms a trimeric structure too. The Y233 is located near the putative trimeric hole. This suggests a regulatory role for this residue, but what that role might be remains unclear.

OMPLA’s operon structure sheds light on other relevant proteins in the urea pathway (see Fig. 1). The gastric and enterohepatic *Helicobacter* species have different operon organizations, as illustrated in Fig. 8. Genes located in the same operon normally show co-expression and tend to be regulated by the same promoter. Price et al. found that the life-cycle of an operon is under strong selection [35] and genes found in the same operon are likely involved in the same process [36, 37]. Gastric *Helicobacters* have a different gene expression compared to enterohepatic *Helicobacters*, including a higher level of urease expression [23]. The hosts of the gastric *Helicobacter* group are more diverse than the hosts of the enterohepatic group, but their *pldA* operons nevertheless are more similar to each other; some consisting of five consecutive genes (2 *SLC6sbd_Tyt1*-*Like* genes, *pldA*, *dnaN,* and *gyrB*). The two transporter (channel) genes upstream of the *pldA* gene are generally predicted to lie in the same operon as *pldA* in gastric *Helicobacters*, as shown in Table 3. The gene encoding AmCI is also predicted to be phase variable (see Table 3). The two gastric bacteria *H. himalayensis* (lacking the urease genes) and *H. mustelae* (lacking the *pldA* gene) and enterohepatic *Helicobacters,* have different organization and they have probably evolved different mechanisms for acid adaptation.

Homology searches in clusters of orthologous groups (COGs) database [37] revealed that AmCI and AmCII both belong to COG0733 (Na^+^-dependent transporters (channels) of the SNF family). Since genes in an operon are regulated together, the two Na^+^-dependent transporters (channels) of the SNF family (AmCI and AmCII) are also likely implicated in acid survival and we suggest that they are involved in ammonium/ammonia transport or diffusion from the cytosol to the periplasm. We do not know why two very similar channels are needed in this process, but visual inspection of their 3D structure models suggests the possibility that one of the channels can be ammonium specific while the other is specific for ammonia. While the OMPLA family (COG2829) is mainly found in *Proteobacteria*, Na^+^-dependent COG0733 channels are widespread throughout bacteria and eukaryotes. In order to better understand the possible role of these proteins in the urea pathway (being co-regulated with OMPLA), 3D model structures were constructed for AmCI and AmCII (see Fig. 9). The AmCI and AmCII transporters have closest sequence similarity with *Bacillus halodurans* MhsT (a BLAST search against the PDB resulted in 31–35% sequence identity for the *H. pylori* HP0497 and HP0498 sequences). MhsT is a Na^+^-dependent neurotransmitter/sodium symporter and belong to the SLC6 family of Na^+^/Cl^−^-dependent neurotransmitter transporters. These proteins transport small substances, e.g. amino acids or similar structures [38, 39]. The substrates found among SLC6 transporters include glycine, serotonin, dopamine, and norepinephrine [40]. They have a so-called 5 + 5 core helix motif that is embedded in the membrane, albeit that we cannot exclude the presence of more helices; MhsT, for example, has 11 helices, while LeuT has 12 helices [38]. Evolutionary, these transporters adjust quickly to changes; some of them, for example, are voltage-gated channels under certain conditions [41]. We believe that the two putative ammonium channels, AmCI and AmCII, are located in the inner membrane, because helical proteins are seldom found in the outer membrane of Gram-negative bacteria [16]. Likely functions include pH sensing, or solute transport of small substances that are involved in buffering the environment [42–46]. We hypothesize that since they are co-regulated with OMPLA, these transporters are involved in the urea pathway, as shown in Fig. 1. Since there are currently no NH_4_^+^ channels known, we believe the two COG0733 are better candidates than the suggested UreI. UreI have an important role in urea influx, but no experiments show a role for UreI in ammonium efflux [42].

## Conclusions

OMPs have multiple functions that aid adaptation in rapidly changing environments [16]. Current literature does not explain which *H. pylori* OMP is involved in maintaining higher periplasmic pH level compared to the acidic outside environment. In silico protein structure modelling of *H. pylori* OMPLA indicates a transmembrane pore of 4 Å, and *H. pylori* OMPLA could participate in acid protection through this transmembrane pore. Our group has previously linked intact *H. pylori* OMPLA to an increased risk for ulcer disease [47]. A possible explanation for this could be its role in the urea pathway that concludes with the efflux of ammonium that is implicated in ulcer formation.

We propose that urea passes through OMPLA into the periplasm, while ammonium might exit to increase the cytoplasmic and periplasmic pH. Since acid tolerance is observed only when OMPLA is intact, we suggest that OMPLA’s role is to maintain an optimal periplasmic pH as modelled in Fig. 9. We hypothesize that AmCI and AmCII, which are co-regulated with OMPLA, are also implicated in acid protection allowing NH_3_/NH_4_^+^ to move from the cytoplasm to the periplasm. We propose that OMPLA also is involved in the secretion of NH_4_^+^ from the cell. That gives OMPLA two transport roles -urea influx and, ammonium efflux- which is possible because it is likely constitutively expressed (as observed in other pathogenic bacteria [33]) which suggests that it probably is abundantly present in the outer membrane.

## Methods

### Survival of *H. pylori* OMPLA variants in acidic environment

We collected *H. pylori* clinical isolates in four hospitals in the Oslo region, Norway [12, 14]. 57 isolates were examined for spontaneous colony variants showing altered phospholipase A activity by thin-layer chromatography (TLC) as previously described [47]. Isogenicity of five selected variant pairs were confirmed by amplified fragment length polymorphism (AFLP) as previously described [12]. From each variant, the *pldA* gene was sequenced as previously described [47], in order to detect the genetic background for the OMPLA phenotype (Table 1). Survival of the selected variants at pH 3.5 was determined as previously described [12]. See Additional file 1 for more information.

### Operon predictions

ProOpDB [36] predicts operon structures of prokaryotic genomes, and presents results in figures in which all genes in a clusters of ortholog genes (COG) have the same colour. We used this software to determine all genes in the same operon as OMPLA. Not all species are represented in this database, so the genes used to analyse gastric vs enterohepatic *Helicobacters* were analysed in Fgenesb (Softberry Inc., Mount Kisco, NY, US). The gene sequences were manually curated using NCBI’s gene-view and BioEdit. We extracted the genes transcribed in the same direction as *pldA* and used Fgenesb to find out which genes likely are co-regulated.

### In silico protein sequence analyses

Structure-based MSAs were produced using the workflow described by Kuipers et al. [18]. This procedure uses core sequence elements (e.g. β-strands and α-helices) to generate an initial profile. The sequences of all homologs (found with Blastp [48]) are aligned iteratively to the profile that is updated after each alignment round. The aligned sequences of the model and template were extracted from the final MSA (see Additional file 2) and used to construct the 3D structure model (see Fig. 2a).

### In silico protein entropy-variability analyses

**Fig. 10:**
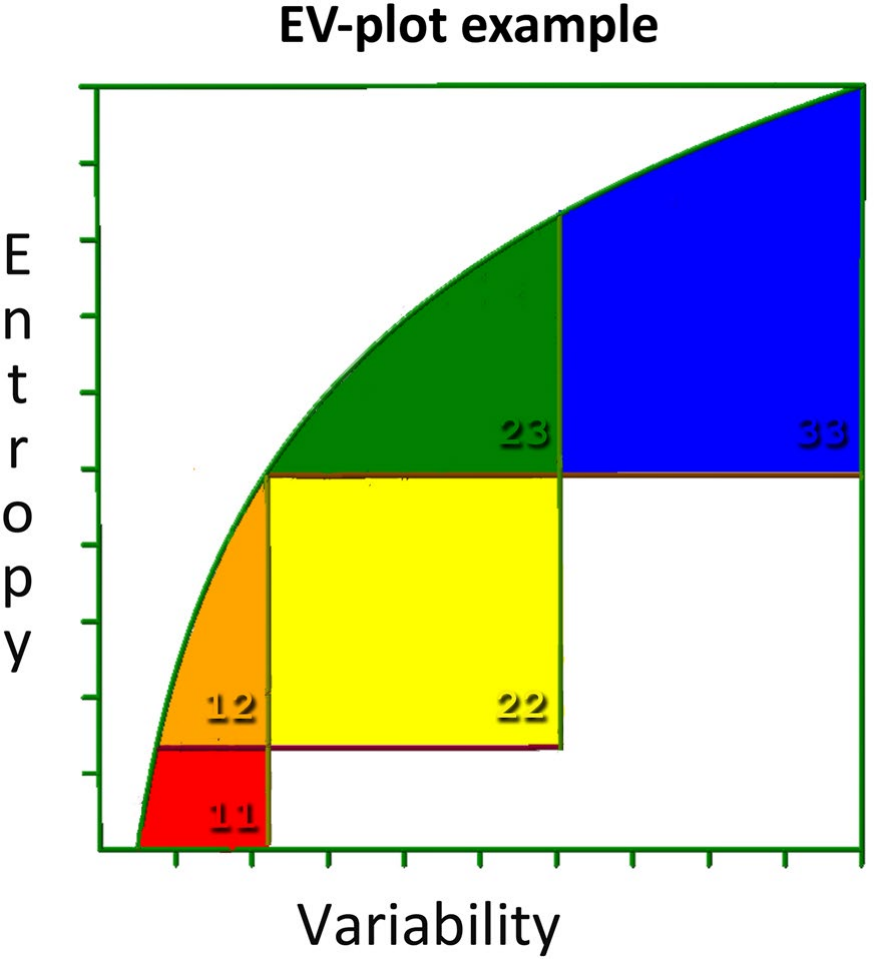
EV-plot to explain the *five different boxes* found in EVA. The main active site residues are found in the *box coloured red* (*box 11*, low variability and low entropy). Residues supporting the active site are *coloured orange* (*box 12*, intermediate entropy and low variability). *Yellow* represents residues involved in communication between the main active site and regulatory sites (*box 22*, intermediate entropy and intermediate variability). The *green area* holds residues involved in regulation (*box 23*, high entropy and intermediate variability). *Blue* represents residues with no known function (*box 33*, high entropy and high variability). In an actual EV-plot, one will find *crosses inside each box* that represents the residues in the consensus sequence of the MSA

The MSA was used to develop an evolutionary model through EVA. The MSA and EVA analyses were performed as described [16, 18] using the YASARA/WHAT IF twinset [49–51]. EVA plots the entropy versus the variability to describe the variability pattern for every residue in the MSA. This plot contains five sectors that each holds residues with common structural and functional characteristic, as shown in Fig. 10 [19].

### OMPLA 3D structure modelling

The *H. pylori* OMPLA model was built for the NCBI: AFR51731 sequence using the 1QD5 [15] (monomer PDB file downloaded from [http://www.rcsb.org] [52]) as template. The sequence alignment underlying the homology modelling was extracted from the aforementioned MSA. All waters, lipids, and crystallization additives were removed from the template file before modelling, and the remaining, reduced monomer was further subjected to the YASARA Clean function in the YASARA/WHAT IF twinset. The actual modelling was performed with the WHAT IF server [swift.cmbi.ru.nl/servers/html] because the sequence alignment was complicated and this server strictly follows the user-given alignment. The md_runmembranefast script in YASARA/WHAT IF twinset was used to optimize all 3D models. The very putative motif of the not-modelled insert was predicted by constructing the entire *H. pylori* OMPLA model automatically in YASARA/WHAT IF twinset, using the hm_build script.

### 3D structure modelling of two genes in the *pldA* operon: AmCI and AmCII

The two Na^+^-dependent channels of the SNF family (AmCI and AmCII; corresponding to the HP0497 and HP0498 genes in *H. pylori* strain 26695) homology models were constructed with YASARA/WHAT IF twinset, as described for *H. pylori* OMPLA. Both models were constructed using the same PDB file 4US3 as template. The alignments were extracted from a single large MSA that was generated as described below.

### In silico protein structure analyses

All structure analyses were performed using the YASARA/WHAT IF twinset. Models and template structures were superposed using the MUSTANG pairwise protein structure aligner [53] as implemented in YASARA. The OMPLA pore was visualized with different substrates with molecular surfaces shown using YASARA/WHAT IF twinset. Pore sizes were estimated using WHAT IF (as implemented in the YASARA/WHAT IF twinset), where solvent exclusion maps were produced for spherical probes with radius P using the surface map option in WHAT IF (srfmap) [49, 54]. PoreWalker was used to estimate possible pore paths (see Fig. 2 in Additional file 3) [55].

## Additional files

**Additional file 1.** Detailed overview of *H. pylori* acid tolerance mechanism (the urea pathway).

**Additional file 2.** OMPLA multiple sequence alignment file.

**Additional file 3.** Homology modelling results.

**Additional file 4.** Residue numbering in *E. coli* and *H. pylori *sequences. structures, and models.

**Additional file 5.** *H. pylori* OMPLA variants survival in acidic environment.

## Abbreviations

αCA: α-carbonic anhydrase
AFLP: amplified fragment length polymorphism
AmCI: ammonium channel I
AmCII: ammonium channel II
COG: clusters of orthologous group
EVA: entropy-variability analyses
IM: inner membrane
MSA: multiple sequence alignment
OM: outer membrane
OMPLA: outer membrane phospholipase A
TLC: thin-layer chromatography
