# Supplementary material for: Outer membrane phospholipase A’s roles in Helicobacter pylori acid adaptation
Source: Gut Pathog. 2017 Jun 12;9:36. doi: 10.1186/s13099-017-0184-y (PMC5469174; doi:10.1186/s13099-017-0184-y)
Supplement: Supplementary file 1 — Additional file 1. Detailed overview of H. pylori acid tolerance mechanism (the urea pathway). [file 13099_2017_184_MOESM1_ESM.docx]

**Additional File 1: Detailed description of *H. pylori* acid tolerance mechanism**

The *H. pylori* acid regulation pathway has been well studied and often debated [1-5]. This section focuses on the influx and outflux of urea, and includes the best characterized steps in the urea pathway together with our model. Figure 1 illustrates our hypothesis for *H. pylori’s* acid resistance that can be logically sub-divided into six steps:

Step A) Gastric epithelial cells produce different metabolites that can be detected by *H. pylori*, including urea, which is the primary metabolite detected by the bacteria through chemotaxis [6]. Flagella combined with the spiral shape of *H. pylori,* make it motile, and it moves away from the harsh acidic conditions towards a chemoattractant, *e.g.* urea [1, 6-8]. Huang *et al.* further identified the high-affinity *H. pylori* TlpB chemoreceptors that detect the smallest amounts of urea, recognizing its presence on a nanomolar-scale [6].

Step B) The proton-gated, pH-dependent, UreI channel located in the inner membrane allows urea to diffuse from the periplasm to the cytoplasm [3]. The transporter is closed at neutral to alkaline pH, but opens below pH 5 [9-11]. UreI is composed of six subunits that form a hexameric ring [11]. The UreI channel’s high substrate specificity is likely due to two constrictions found inside the channels that can be gated by internal hydrogen bonds [5]. Cáceres-Delpiano *et al.* [12] concluded that UreI cooperativity is critical for channel activity in an acidic environment, and that salt bridge formation in the periplasmic loops is also likely to contribute to pH-regulated gating. UreI forms a complex with urease at the cytoplasmic side of the membrane [3, 13]. As cytoplasmic urease uses up this urea (see step C) the gradient will remain intact.

Step C) Cytoplasmic urease converts urea and water into ammonia and carbon dioxide [14-17]. Urease exists in a variety of species, including plants and bacteria [15, 18]. The first crystallized urease was isolated from jack beans (*Canavalia ensiformis*) in 1926 [19]. *H. pylori* urease was crystallized in 2001 [20], just a few years after Bauerfeind *et al.* found that 10% of the total protein in *H. pylori* is cytoplasmic urease [21]. This 1.1 megadalton, spherical, dodecameric, metalloenzyme has 12 catalytic sites (α_12_β_12_) [20]. Nickel is incorporated into the active site through accessory proteins encoded by the *ureIEFGH* operon [22-26]. These accessory proteins are involved in urease stabilization (UreH) preventing premature Ni^2+^-binding to the active site (UreF), and Ni^2+^ incorporation into the active centre (UreE), while energy required for this process is provided by UreG [27]. Other accessory proteins that are activated by the NikR transcription factor and involved in obtaining nickel are the NixA nickel uptake protein, the nickel storage proteins (Hpn, HpnI and HspA) and the HypAB hydrogenase accessory protein required for NiFe hydrogenase activation [27]. Urease is activated when proton leakage drops the cytoplasmic pH till below 4.5 [3]. Nickel is incorporated after urease has been assembled at the membrane by the accessory proteins [3], where it then interacts with the pH-regulated UreI channel [28, 29]. When the pH falls below 5.0 this proton-gated channel opens to allow urea to flow into the cytoplasm.

The urease enzyme produces a short-lived intermediate molecule, carbamic acid and ammonia (CO(NH_2_)_2_ + H_2_O 🡪 NH_2_COOH + NH_3_); then, the carbamic acid is spontaneously converted to carbonic acid and ammonia (NH_2_COOH + H_2_O 🡪H_2_CO_3_ +NH_3_); finally, the carbonic acid is converted to water and carbon dioxide (H_2_CO_3_ ↔ H_2_O + CO_2_) [30]. Urease is an efficient enzyme [4, 31], found in abundance [6, 21], and bound to the membrane via UreI-interaction [3]. This locates it close to the source of its substrate. Urease activity is UreI-dependent and acidic UreI activation yields a 300-fold increase in urease activity [30, 32, 33].

Step D) Their gradients drive the diffusion of CO_2_ and NH_3_ (and the NH_4_^+^ that will form spontaneously) from the cytoplasm to the periplasm. CO_2_ will diffuse spontaneously through the IM [34], but ammonia/ammonium will not [35, 36] so that at least an IM channel for NH_4_^+^ must exist [31, 37]. Although ammonia might diffuse through the membrane [3, 34], it seems more efficient for the H^+^ efflux if only ammonium can leave the cytosol easily [36]. We hypothesize that the two COG0733 channels (Ammonium Channels I and II; AmCI and AmCII) encoded in the same operon as OMPLA are involved in NH_4_^+^ diffusion. The NH_3_ + H_3_O^+^ <-> NH_4_^+^ + H_2_O reaction will keep shifting to the right when ammonium moves to the periplasm.

Step E) Periplasmic α-carbonic anhydrase (αCA) converts carbon dioxide and water into bicarbonate and protons. The bicarbonate buffers the periplasmic pH around 6.1 [1, 3, 4, 6, 38]. Marcus *et al.* found in 2016 that the two-component ArsRS system that regulates genes involved in acid acclimation activates αCA [39, 40]. αCA is a membrane-bound dimer that is likely located near the cytoplasmic urease [30, 41]. The αCA active site is situated in a funnel-shaped pocket where a zinc ion is found interacting with three His-residues [42].

Step F) Ammonium diffuses into the environment. This removes one proton from the cell per ammonium. We suggest that OMPLA is the ammonium channel. Once outside the cell, ammonium converts the gel-like substance of the gastric mucosa to a liquid state [43, 44], enabling easily movement of *H. pylori* to the epithelial cells where ammonium causes ulceration [45, 46], and even, on the long run, promotes gastric cancer [47-50]. Literature has strongly implicated ammonium in these pathogeneses [51-58].

**References**

1. Fischer F, De Reuse H: Adaptation of *Helicobacter pylori* metabolism to persistent gastric colonization. In *Helicobacter pylori* Research: From Bench to Bedside. Backert S, Yamaoka Y, editors. Tokyo: Springer Japan; 2016: 29-56

2. Bury-Moné S, Mendz GL, Ball GE, Thibonnier M, Stingl K, Ecobichon C, Avé P, Huerre M, Labigne A, Thiberge JM, De Reuse H. Roles of α and β-carbonic anhydrases of *Helicobacter pylori* in the urease-dependent response to acidity and in colonization of the murine gastric mucosa. Infect Immun. 2008; 76:497-509.

3. Scott DR, Marcus EA, Wen Y, Singh S, Feng J, Sachs G. Cytoplasmic histidine kinase (HP0244)-regulated assembly of urease with UreI, a channel for urea and its metabolites, CO_2_, NH_3_, and NH_4_^+^, is necessary for acid survival of *Helicobacter pylori*. J Bacteriol. 2010; 192:94-103.

4. Lund P, Tramonti A, De Biase D. Coping with low pH: molecular strategies in neutralophilic bacteria. FEMS Microbiol Rev. 2014; 38:1091-1125.

5. McNulty R, Ulmschneider JP, Luecke H, Ulmschneider MB. Mechanisms of molecular transport through the urea channel of *Helicobacter pylori*. Nat Commun. 2013; 4:2900.

6. Huang JY, Sweeney EG, Sigal M, Zhang HC, Remington SJ, Cantrell MA, Kuo CJ, Guillemin K, Amieva MR. Chemodetection and destruction of host urea allows *Helicobacter pylori* to locate the epithelium. Cell Host Microbe. 2015; 18:147-156.

7. Scott DR, Marcus EA, Wen Y, Oh J, Sachs G. Gene expression in vivo shows that *Helicobacter pylori* colonizes an acidic niche on the gastric surface. Proc Natl Acad Sci U S A. 2007; 104:7235-7240.

8. Kao CY, Sheu BS, Wu JJ. *Helicobacter pylori* infection: An overview of bacterial virulence factors and pathogenesis. Biomed J. 2016; 39:14-23.

9. Scott DR, Marcus EA, Weeks DL, Lee A, Melchers K, Sachs G. Expression of the *Helicobacter pylori ureI* gene is required for acidic pH activation of cytoplasmic urease. Infect Immun. 2000; 68:470-477.

10. Weeks DL, Eskandari S, Scott DR, Sachs G. A H+-gated urea channel: the link between *Helicobacter pylori* urease and gastric colonization. Science. 2000; 287:482-485.

11. Strugatsky D, McNulty R, Munson K, Chen CK, Soltis SM, Sachs G, Luecke H. Structure of the proton-gated urea channel from the gastric pathogen *Helicobacter pylori*. Nature. 2013; 493:255-258.

12. Cáceres-Delpiano J, Teneb J, Mansilla R, García A, Salas-Burgos A. Variations in periplasmic loop interactions determine the pH-dependent activity of the hexameric urea transporter UreI from *Helicobacter pylori*: a molecular dynamics study. BMC Struct Biol. 2015; 15.

13. Scott DR, Marcus EA, Weeks DL, Sachs G. Mechanisms of acid resistance due to the urease system of *Helicobacter pylori*. Gastroenterology. 2002; 123:187-195.

14. Keilberg D, Ottemann KM. *H. pylori* GPS: Modulating host metabolites for location sensing. Cell Host Microbe. 2015; 18:135-136.

15. Krajewska B. Ureases I. Functional, catalytic and kinetic properties: A review. J Mol Catal B Enzym. 2009; 59:9-21.

16. Mobley HLT: Urease. In *Helicobacter pylori*: Physiology and Genetics. Mobley HL, T., Mendz GL, Hazell SL, editors. Washington (DC): ASM Press; 2001: Chapter 16. .

17. Miederer SE, Grübel P. Profound increase of *Helicobacter pylori* urease activity in gastric antral mucosa at low pH. Dig Dis Sci. 1996; 41:944-949.

18. Fahey JW, Stephenson KK, Wade KL, Talalay P. Urease from *Helicobacter pylori* is inactivated by sulforaphane and other isothiocyanates. Biochem Biophys Res Commun. 2013; 435:1-7.

19. Sumner JB. The isolation and crystallization of the enzyme urease preliminary paper. J Biol Chem. 1926; 69:435-441.

20. Schauer DB: Enterohepatic *Helicobacter* species. In *Helicobacter pylori*: Physiology and Genetics. Mobley HLT, Mendz GL, Hazell SL, editors. Washington (DC): ASM Press; 2001: 43.

21. Bauerfeind P, Garner R, Dunn BE, Mobley HL. Synthesis and activity of *Helicobacter pylori* urease and catalase at low pH. Gut. 1997; 40:25-30.

22. Akada JK, Shirai M, Takeuchi H, Tsuda M, Nakazawa T. Identification of the urease operon in *Helicobacter pylori* and its control by mRNA decay in response to pH. Mol Microbiol. 2000; 36:1071-1084.

23. Park JU, Song JY, Kwon YC, Chung MJ, Jun JS, Park JW, Park SG, Hwang HR, Choi SH, Baik SC, et al. Effect of the urease accessory genes on activation of the *Helicobacter pylori* urease apoprotein. Mol Cells. 2005; 20:371-377.

24. Dunne C, Dolan B, Clyne M. Factors that mediate colonization of the human stomach by *Helicobacter pylori*. World J Gastroenterol. 2014; 20:5610-5624.

25. Wen Y, Feng J, Sachs G. *Helicobacter pylori* 5'ureB-sRNA, a *cis*-encoded antisense small RNA, negatively regulates ureAB expression by transcription termination. J Bacteriol. 2013; 195:444-452.

26. Ralston A. Operons and prokaryotic gene regulation. Nature Education 2008; 1:216.

27. Ge RG, Wang DX, Hao MC, Sun XS. Nickel trafficking system responsible for urease maturation in *Helicobacter pylori*. World J Gastroenterol. 2013; 19:8211-8218.

28. Stingl K, Altendorf K, Bakker EP. Acid survival of *Helicobacter pylori*: how does urease activity trigger cytoplasmic pH homeostasis? Trends Microbiol. 2002; 10:70-74.

29. Marcus EA, Scott DR: Gastric colonization by *H. pylori*. In *Helicobacter pylori*. Kim N, editors. Singapore: Springer Singapore; 2016: 23-34Part II.

30. Marcus EA, Moshfegh AP, Sachs G, Scott DR. The periplasmic α-carbonic anhydrase activity of *Helicobacter pylori* is essential for acid acclimation. J Bacteriol. 2005; 187:729-738.

31. Miller EF, Maier RJ. Ammonium metabolism enzymes aid *Helicobacter pylori* acid resistance. J Bacteriol. 2014; 196:3074-3081.

32. Rektorschek M, Buhmann A, Weeks D, Schwan D, Bensch KW, Eskandari S, Scott D, Sachs G, Melchers K. Acid resistance of *Helicobacter pylori* depends on the UreI membrane protein and an inner membrane proton barrier. Mol Microbiol. 2000; 36:141-152.

33. Skouloubris S, Thiberge JM, Labigne A, De Reuse H. The *Helicobacter pylori* UreI protein is not involved in urease activity but is essential for bacterial survival *in vivo*. Infect Immun. 1998; 66:4517-4521.

34. Missner A. Carbon dioxide transport through membranes. J Biol Chem. 2008; 283:25340-25347.

35. Kleiner D. The transport of NH_3_ and NH_4_^+^ across biological membranes. Biochim Biophys Acta. 1981; 639:41-52.

36. Athmann C, Zeng N, Kang T, Marcus EA, Scott DR, Rektorschek M, Buhmann A, Melchers K, Sachs G. Local pH elevation mediated by the intrabacterial urease of *Helicobacter pylori* cocultured with gastric cells. J Clin Invest. 2000; 106:339-347.

37. Lytton SD, Fischer W, Nagel W, Haas R, Beck FX. Production of ammonium by *Helicobacter pylori* mediates occludin processing and disruption of tight junctions in Caco-2 cells. Microbiology. 2005; 151:3267-3276.

38. Modak JK, Liu YC, Machuca MA, Supuran CT, Roujeinikova A. Structural basis for the inhibition of *Helicobacter pylori* α-carbonic anhydrase by sulfonamides. PLoS One. 2015; 10:e0127149.

39. Marcus EA, Sachs G, Wen Y, Scott DR. Phosphorylation-dependent and phosphorylation-independent regulation of *Helicobacter pylori* acid acclimation by the ArsRS two-component system. *Helicobacter*. 2016; 21:69-81.

40. Pflock M, Finsterer N, Joseph B, Mollenkopf H, Meyer TF, Beier D. Characterization of the ArsRS regulon of *Helicobacter pylori*, involved in acid adaptation. J Bacteriol. 2006; 188:3449-3462.

41. Compostella ME, Berto P, Vallese F, Zanotti G. Structure of α-carbonic anhydrase from the human pathogen *Helicobacter pylori*. Acta Crystallogr F Struct Biol Commun. 2015; 71:1005-1011.

42. Modak JK, Liu YC, Supuran CT, Roujeinikova A. Structure-activity relationship for sulfonamide Inhibition of *Helicobacter pylori* α-carbonic anhydrase. J Med Chem. 2016; 59:11098-11109.

43. Salama NR, Hartung ML, Muller A. Life in the human stomach: persistence strategies of the bacterial pathogen *Helicobacter pylori*. Nat Rev Microbiol. 2013; 11:385-399.

44. Celli JP, Turner BS, Afdhal NH, Keates S, Ghiran I, Kelly CP, Ewoldt RH, McKinley GH, So P, Erramilli S, Bansil R. *Helicobacter pylori* moves through mucus by reducing mucin viscoelasticity. Proc Natl Acad Sci U S A. 2009; 106:14321-14326.

45. Oyake J, Otaka M, Matsuhashi T, Jin M, Odashima M, Komatsu K, Wada I, Horikawa Y, Ohba R, Hatakeyama N, et al. Over-expression of 70-kDa heat shock protein confers protection against monochloramine-induced gastric mucosal cell injury. Life Sci. 2006; 79:300-305.

46. Caron TJ, Scott KE, Fox JG, Hagen SJ. Tight junction disruption: *Helicobacter pylori* and dysregulation of the gastric mucosal barrier. World J Gastroenterol. 2015; 21:11411-11427.

47. Tsujii M, Kawano S, Tsuji S, Nagano K, Ito T, Hayashi N, Fusamoto H, Kamada T, Tamura K. Ammonia: a possible promotor in *Helicobacter pylori*-related gastric carcinogenesis. Cancer Lett. 1992; 65:15-18.

48. Asaka M, Sepulveda AR, Sugiyama T, Graham DY: Gastric Cancer. In *Helicobacter pylori*: Physiology and Genetics. Mobley HLT, Mendz GL, Hazell SL, editors. Washington (DC): ASM Press; 2001:

49. Dunn BE. Pathogenic mechanisms of *Helicobacter pylori*. Gastroenterol Clin North Am. 1993; 22:43-57.

50. Aydemir S, Ozdemir BH, Gur G, Dogan I, Yilmaz U, Boyacioglu S. Effects of *Helicobacter pylori* infection on gastric epithelial cell kinetics in patients with chronic renal failure. World J Gastroenterol. 2005; 11:7183-7187.

51. Ling SS, Khoo LH, Hwang LA, Yeoh KG, Ho B. Instrumental Role of *Helicobacter pylori* γ-Glutamyl Transpeptidase in VacA-Dependent Vacuolation in Gastric Epithelial Cells. PLoS One. 2015; 10:e0131460.

52. Desai MA, Vadgama PM. Enhanced H^+^ diffusion by NH_4_^+^/HCO_3_^-^: implications for *Helicobacter-pylori*-associated peptic ulceration. Digestion. 1993; 54:32-39.

53. Dekigai H, Murakami M, Kita T. Mechanism of *Helicobacter pylori*-associated gastric mucosal injury. Dig Dis Sci. 1995; 40:1332-1339.

54. Kato S, Nishiwaki H, Konaka A, Takeuchi K. Mucosal ulcerogenic action of monochloramine in rat stomachs: Effects of polaprezinc and sucralfate. Dig Dis Sci. 1997; 42:2156-2163.

55. Sato K, Watanabe S, Yoshizawa T, Hirose M, Murai T, Sato N. Ammonia, hydrogen peroxide, and monochloramine retard gastric epithelial restoration in rabbit cultured cell model. Dig Dis Sci. 1999; 44:2429-2434.

56. Suzuki M, Miura S, Suematsu M, Fukumura D, Kurose I, Suzuki H, Kai A, Kudoh Y, Ohashi M, Tsuchiya M. *Helicobacter pylori*-associated ammonia production enhances neutrophil-dependent gastric mucosal cell injury. Am J Physiol. 1992; 263:G719-725.

57. Mobley HL. The role of *Helicobacter pylori* urease in the pathogenesis of gastritis and peptic ulceration. Aliment Pharmacol Ther. 1996; 10 Suppl 1:57-64.

58. Blusiewicz K, Rydzewska G, Rydzewski A. Gastric juice ammonia and urea concentrations and their relation to gastric mucosa injury in patients maintained on chronic hemodialysis. Rocz Akad Med Bialymst. 2005; 50:188-192.
