# Supplementary material for: Outer membrane phospholipase A’s roles in Helicobacter pylori acid adaptation
Source: Gut Pathog. 2017 Jun 12;9:36. doi: 10.1186/s13099-017-0184-y (PMC5469174; doi:10.1186/s13099-017-0184-y)
Supplement: Supplementary file 2 — Additional file 2. OMPLA multiple sequence alignment file. [file 13099_2017_184_MOESM2_ESM.pdf]

```
CONSENSUS                      PPQKAPKEHSWVDFAKKYLNYMDYLGTYFLPNYHSFTPIFQWYHPNINPYQRNEAKFQISFKVPLFRGILGTNGDLYFAYTQTSWWQIYNSEQSAPFRETNYEPELFYVYPTNYKLFGGNFREIGIGLNHQSNGRSGPLSRSWNRVYFRLGFEKGNLAIMVRPWYRIPEDDDN
AFR51838_1_pir_________________PPQKAPKEHSWVDFAKKYLNMMDYLGTYFLPFYHSFTPIFQWYHPNINPYQRNEFKFQISFRVPVFRHILWTKGTLYLAYTQTNWFQIYNDPQSAPMRMINFMPELIYVYPINFKPFggNFSEIWIGWQHISNGVGGAqvSAGQRPVFRLVWEKGGLKIMVAYWPYIPYDQSN
AFR51879_1_pir_________________PPQKAPKEHSWVDFAKKYLNMMDYLGTYFLPFYHSFTPIFQWYHPNINPYQRNEFKFQISFRVPVFRHILWTKGTLYLAYTQTNWFQIYNDPQSAPMRMINFMPELIYVYPINFKPFggNFSEIWIGWQHISNGVGGAqvSAGQRPVFRLVWEKGGLKIMVAYWPYIPYDQSN
WP_000843885_1_pir_____________PPQKAPKEHSWVDFAKKYLNMMDYLGTYFLPFYHSFTPIFQWYHPNINPYQRNEFKFQISFRVPVFRHILWTKGTLYLAYTQTNWFQIYNDPQSAPMRMINFMPELIYVYPINFKPFggNFSEIWIGWQHISNGVGGAqvSAGQRPVFRLVWEKGGLKIMVAYWPYIPYDQSN
WP_000843888_1_pir_____________PPQKAPKEHSWVDFAKKYLNMMDYLGTYFLPFYHSFTPIFQWYHPNINPYQRNEFKFQISFRVPVFRHILWTKGTLYLAYTQTNWFQIYNDPQSAPMRMINFMPELIYVYPINFKPFggNFSEIWIGWQHISNGVGGAqvSAGQRPVFRLVWEKGGLKIMVAYWPYIPYDQSN
WP_000843894_1_pir_____________PPQKAPKEHSWVDFAKKYLNMMDYLGTYFLPFYHSFTPIFQWYHPNINPYQRNEFKFQISFRVPVFRHILWTKGTLYLAYTQTNWFQIYNDPQSAPMRMINFMPELIYVYPINFKPFggNFSEIWIGWQHISNGVGGAqvSAGQRPVFRLVWEKGGLKIMVAYWPYIPYDQSN
WP_000843906_1_pir_____________PPQKAPKEHSWVDFAKKYLNMMDYLGTYFLPFYHSFTPIFQWYHPNINPYQRNEFKFQISFRVPVFRHILWTKGTLYLAYTQTNWFQIYNDPQSAPMRMINFMPELIYVYPINFKPFggNFSEIWIGWQHISNGVGGAqvSAGQRPVFRLVWEKGGLKIMVAYWPYIPYDQSN
WP_001918167_1_pir_____________PPQKAPKEHSWVDFAKKYLNMMDYLGTYFLPFYHSFTPIFQWYHPNINPYQRNEFKFQISFRVPVFRHILWTKGTLYLAYTQTNWFQIYNDPQSAPMRMINFMPELIYVYPINFKPFggNFSEIWIGWQHISNGVGGAqvSAGQRPVFRLVWEKGGLKIMVAYWPYIPYDQSN
WP_001920834_1_pir_____________PPQKAPKEHSWVDFAKKYLNMMDYLGTYFLPFYHSFTPIFQWYHPNINPYQRNEFKFQISFRVPVFRHILWTKGTLYLAYTQTNWFQIYNDPQSAPMRMINFMPELIYVYPINFKPFggNFSEIWIGWQHISNGVGGAqvSAGQRPVFRLVWEKGGLKIMVAYWPYIPYDQSN
WP_001933454_1_pir_____________PPQKAPKEHSWVDFAKKYLNMMDYLGTYFLPFYHSFTPIFQWYHPNINPYQRNEFKFQISFRVPVFRHILWTKGTLYLAYTQTNWFQIYNDPQSAPMRMINFMPELIYVYPINFKPFggNFSEIWIGWQHISNGVGGAqvSAGQRPVFRLVWEKGGLKIMVAYWPYIPYDQSN
WP_001952424_1_pir_____________PPQKAPKEHSWVDFAKKYLNMMDYLGTYFLPFYHSFTPIFQWYHPNINPYQRNEFKFQISFRVPVFRHILWTKGTLYLAYTQTNWFQIYNDPQSAPMRMINFMPELIYVYPINFKPFggNFSEIWIGWQHISNGVGGAqvSAGQRPVFRLVWEKGGLKIMVAYWPYIPYDQSN
WP_001967847_1_pir_____________PPQKAPKEHSWVDFAKKYLNMMDYLGTYFLPFYHSFTPIFQWYHPNINPYQRNEFKFQISFRVPVFRHILWTKGTLYLAYTQTNWFQIYNDPQSAPMRMINFMPELIYVYPINFKPFggNFSEIWIGWQHISNGVGGAqvSAGQRPVFRLVWEKGGLKIMVAYWPYIPYDQSN
WP_021175677_1_pir_____________PPQKAPKEHSWVDFAKKYLNMMDYLGTYFLPFYHSFTPIFQWYHPNINPYQRNEFKFQISFRVPVFRHILWTKGTLYLAYTQTNWFQIYNDPQSAPMRMINFMPELIYVYPINFKPFggNFSEIWIGWQHISNGVGGAqvSAGQRPVFRLVWEKGGLKIMVAYWPYIPYDQSN
WP_033595635_1_pir_____________PPQKAPKEHSWVDFAKKYLNMMDYLGTYFLPFYHSFTPIFQWYHPNINPYQRNEFKFQISFRVPVFRHILWTKGTLYLAYTQTNWFQIYNDPQSAPMRMINFMPELIYVYPINFKPFggNFSEIWIGWQHISNGVGGAqvSAGQRPVFRLVWEKGGLKIMVAYWPYIPYDQSN
WP_033747381_1_pir_____________PPQKAPKEHSWVDFAKKYLNMMDYLGTYFLPFYHSFTPIFQWYHPNINPYQRNEFKFQISFRVPVFRHILWTKGTLYLAYTQTNWFQIYNDPQSAPMRMINFMPELIYVYPINFKPFggNFSEIWIGWQHISNGVGGAqvSAGQRPVFRLVWEKGGLKIMVAYWPYIPYDQSN
WP_033744261_1_pir_____________PPQKAPKEHSWVDFAKKYLNMMDYLGTYFLPFYHSFTPIFQWYHPNINPYQRNEFKFQISFRVPVFRHILWTKGTLYLAYTQTNWFQIYNDPQSAPMRMINFMPELIYVYPINFKPFggNFSEIWIGWQHISNGVGGAqvSAGQRPVFRLVWEKGGLKIMVAYWPYIPYDQSN
AFR51861_1_pir_________________PPQKAPKEHSWVDFAKKYLNMMDYLGTYFLPFYHSFTPIFQWYHPNINPYQRNEFKFQISFRVPVFRHILWTKGTLYLAYTQTDWFQIYNDPQSAPMRMINFMPELIYVYPINFKPFggNFSEIWIGWQHISNGVGGAqvSAGQRPVFRLVWEKGGLKIMVAYWPYIPYDQSN
WP_033758177_1_pir_____________PPQKAPKEHSWVDFAKKYLNMMDYLGTYFLPFYHSFTPIFQWYHPNINPYQRNEFKFQISFRVPVFRHILWTKGTLYLAYTQTDWFQIYNDPQSAPMRMINFMPELIYVYPINFKPFggNFSEIWIGWQHISNGVGGAqvSAGQRPVFRLVWEKGGLKIMVAYWPYIPYDQSN
WP_039085261_1_pir_____________PPQKAPKEHSWVDFAKKYLNMMDYLGTYFLPFYHSFTPIFQWYHPNINPYQRNEFKFQISFRVPVFRHILWTKGTLYLAYTQTNWFQIYNNPQSAPMRMMNFMPELIYVYPINFKPFggNFSEIWIGWQHISNGVGGalVSAGQRPVFRLVWEKGGLKIMVAYWPYVPYDQSN
WP_039085261_1_pir_____________PPQKAPKEHSWVDFAKKYLNMMDYLGTYFLPFYHSFTPIFQWYHPNINPYQRNEFKFQISFRVPVFRHILWTKGTLYLAYTQTNWFQIYNNPQSAPMRMMNFMPELIYVYPINFKPFggNFSEIWIGWQHISNGVGGalVSAGQRPVFRLVWEKGGLKIMVAYWPYVPYDQSN
WP_000836606_1_pir_____________PPQKAPKEHSWVDFAKKYLNMMDYLGTYFLPFYHSFTPIFQWYHPNINPYQRNEFKFQISFRVPVFRHILWTKGTLYLAYTQTNWFQIYNNPQSAPMRMINFMPELIYVYPINFKPFggNFSEIWIGWQHISNGVGGAqvSAGQRPVFRLVWEKGGLKIMVAYWPYIPYDQSN
WP_000836611_1_pir_____________PPQKAPKEHSWVDFAKKYLNMMDYLGTYFLPFYHSFTPIFQWYHPNINPYQRNEFKFQISFRVPVFRHILWTKGTLYLAYTQTNWFQIYNNPQSAPMRMINFMPELIYVYPINFKPFggNFSEIWIGWQHISNGVGGAqvSAGQRPVFRLVWEKGGLKIMVAYWPYIPYDQSN
WP_033598247_1_pir_____________PPQKAPKEHSWVDFAKKYLNMMDYLGTYFLPFYHSFTPIFQWYHPNINPYQRNEFKFQISFRVPVFRHILWTKGTLYLAYTQTNWFQIYNNPQSAPMRMINFMPELIYVYPINFKPFggNFSEIWIGWQHISNGVGGAqvSAGQRPVFRLVWEKGGLKIMVAYWPYIPYDQSN
WP_033600075_1_pir_____________PPQKAPKEHSWVDFAKKYLNMMDYLGTYFLPFYHSFTPIFQWYHPNINPYQRNEFKFQISFRVPVFRHILWTKGTLYLAYTQTNWFQIYNNPQSAPMRMINFMPELIYVYPINFKPFggNFSEIWIGWQHISNGVGGAqvSAGQRPVFRLVWEKGGLKIMVAYWPYIPYDQSN
WP_033621121_1_pir_____________PPQKAPKEHSWVDFAKKYLNMMDYLGTYFLPFYHSFTPIFQWYHPNINPYQRNEFKFQISFRVPVFRHILWTKGTLYLAYTQTNWFQIYNNPQSAPMRMINFMPELIYVYPINFKPFggNFSEIWIGWQHISNGVGGAqvSAGQRPVFRLVWEKGGLKIMVAYWPYIPYDQSN
AFR51763_1_pir_________________PPQKAPKEHSWVDFAKKYLNMMDYLGTYFLPFYHSFTPIFQWYHPNINPYQRNEFKFQISFRVPVFRHILWTKGTLYLAYTQTNWFQIYNDPQSAPMRMINFMPELIYVYPINFKPFggNFSEIWIGWQHISNGVGGAqvSAGQRPVFRLVWEKGGLKIMVAYWPYVPYDQSN
AFR51763_1_pir_________________PPQKAPKEHSWVDFAKKYLNMMDYLGTYFLPFYHSFTPIFQWYHPNINPYQRNEFKFQISFRVPVFRHILWTKGTLYLAYTQTNWFQIYNDPQSAPMRMINFMPELIYVYPINFKPFggNFSEIWIGWQHISNGVGGAqvSAGQRPVFRLVWEKGGLKIMVAYWPYVPYDQSN
AFR51784_1_pir_________________PPQKAPKEHSWVDFAKKYLNMMDYLGTYFLPFYHSFTPIFQWYHPNINPYQRNEFKFQISFRVPVFRHILWTKGTLYLAYTQTNWFQIYNDPQSAPMRMINFMPELIYVYPINFKPFggNFSEIWIGWQHISNGVGGAqvSAGQRPVFRLVWEKGGLKIMVAYWPYVPYDQSN
WP_000836102_1_pir_____________PPQKAPKEHSWVDFAKKYLNMMDYLGTYFLPFYHSFTPIFQWYHPNINPYQRNEFKFQISFRVPVFRHILWTKGTLYLAYTQTNWFQIYNDPQSAPMRMINFMPELIYVYPINFKPFggNFSEIWIGWQHISNGVGGAqvSAGQRPVFRLVWEKGGLKIMVAYWPYVPYDQSN
WP_000843883_1_pir_____________PPQKAPKEHSWVDFAKKYLNMMDYLGTYFLPFYHSFTPIFQWYHPNINPYQRNEFKFQISFRVPVFRHILWTKGTLYLAYTQTNWFQIYNDPQSAPMRMINFMPELIYVYPINFKPFggNFSEIWIGWQHISNGVGGAqvSAGQRPVFRLVWEKGGLKIMVAYWPYVPYDQSN
WP_001923084_1_pir_____________PPQKAPKEHSWVDFAKKYLNMMDYLGTYFLPFYHSFTPIFQWYHPNINPYQRNEFKFQISFRVPVFRHILWTKGTLYLAYTQTNWFQIYNDPQSAPMRMINFMPELIYVYPINFKPFggNFSEIWIGWQHISNGVGGAqvSAGQRPVFRLVWEKGGLKIMVAYWPYVPYDQSN
WP_001933891_1_pir_____________PPQKAPKEHSWVDFAKKYLNMMDYLGTYFLPFYHSFTPIFQWYHPNINPYQRNEFKFQISFRVPVFRHILWTKGTLYLAYTQTNWFQIYNDPQSAPMRMINFMPELIYVYPINFKPFggNFSEIWIGWQHISNGVGGAqvSAGQRPVFRLVWEKGGLKIMVAYWPYVPYDQSN
WP_001942860_1_pir_____________PPQKAPKEHSWVDFAKKYLNMMDYLGTYFLPFYHSFTPIFQWYHPNINPYQRNEFKFQISFRVPVFRHILWTKGTLYLAYTQTNWFQIYNDPQSAPMRMINFMPELIYVYPINFKPFggNFSEIWIGWQHISNGVGGAqvSAGQRPVFRLVWEKGGLKIMVAYWPYVPYDQSN
WP_001947754_1_pir_____________PPQKAPKEHSWVDFAKKYLNMMDYLGTYFLPFYHSFTPIFQWYHPNINPYQRNEFKFQISFRVPVFRHILWTKGTLYLAYTQTNWFQIYNDPQSAPMRMINFMPELIYVYPINFKPFggNFSEIWIGWQHISNGVGGAqvSAGQRPVFRLVWEKGGLKIMVAYWPYVPYDQSN
WP_001980853_1_pir_____________PPQKAPKEHSWVDFAKKYLNMMDYLGTYFLPFYHSFTPIFQWYHPNINPYQRNEFKFQISFRVPVFRHILWTKGTLYLAYTQTNWFQIYNDPQSAPMRMINFMPELIYVYPINFKPFggNFSEIWIGWQHISNGVGGAqvSAGQRPVFRLVWEKGGLKIMVAYWPYVPYDQSN
WP_002207234_1_pir_____________PPQKAPKEHSWVDFAKKYLNMMDYLGTYFLPFYHSFTPIFQWYHPNINPYQRNEFKFQISFRVPVFRHILWTKGTLYLAYTQTNWFQIYNDPQSAPMRMINFMPELIYVYPINFKPFggNFSEIWIGWQHISNGVGGAqvSAGQRPVFRLVWEKGGLKIMVAYWPYVPYDQSN
WP_033756333_1_pir_____________PPQKAPKEHSWVDFAKKYLNMMDYLGTYFLPFYHSFTPIFQWYHPNINPYQRNEFKFQISFRVPVFRHILWTKGTLYLAYTQTNWFQIYNDPQSAPMRMINFMPELIYVYPINFKPFggNFSEIWIGWQHISNGVGGAqvSAGQRPVFRLVWEKGGLKIMVAYWPYVPYDQSN
WP_033761380_1_pir_____________PPQKAPKEHSWVDFAKKYLNMMDYLGTYFLPFYHSFTPIFQWYHPNINPYQRNEFKFQISFRVPVFRHILWTKGTLYLAYTQTNWFQIYNDPQSAPMRMINFMPELIYVYPINFKPFggNFSEIWIGWQHISNGVGGAqvSAGQRPVFRLVWEKGGLKIMVAYWPYVPYDQSN
WP_033765523_1_pir_____________PPQKAPKEHSWVDFAKKYLNMMDYLGTYFLPFYHSFTPIFQWYHPNINPYQRNEFKFQISFRVPVFRHILWTKGTLYLAYTQTNWFQIYNDPQSAPMRMINFMPELIYVYPINFKPFggNFSEIWIGWQHISNGVGGAqvSAGQRPVFRLVWEKGGLKIMVAYWPYVPYDQSN
WP_033769204_1_pir_____________PPQKAPKEHSWVDFAKKYLNMMDYLGTYFLPFYHSFTPIFQWYHPNINPYQRNEFKFQISFRVPVFRHILWTKGTLYLAYTQTNWFQIYNDPQSAPMRMINFMPELIYVYPINFKPFggNFSEIWIGWQHISNGVGGAqvSAGQRPVFRLVWEKGGLKIMVAYWPYVPYDQSN
WP_038417456_1_pir_____________PPQKAPKEHSWVDFAKKYLNMMDYLGTYFLPFYHSFTPIFQWYHPNINPYQRNEFKFQISFRVPVFRHILWTKGTLYLAYTQTNWFQIYNDPQSAPMRMINFMPELIYVYPINFKPFggNFSEIWIGWQHISNGVGGAqvSAGQRPVFRLVWEKGGLKIMVAYWPYVPYDQSN
WP_053576369_1_pir_____________PPQKAPKEHSWVDFAKKYLNMMDYLGTYFLPFYHSFTPIFQWYHPNINPYQRNEFKFQISFRVPVFRHILWTKGTLYLAYTQTNWFQIYNDPQSAPMRMINFMPELIYVYPINFKPFggNFSEIWIGWQHISNGVGGAqvSAGQRPVFRLVWEKGGLKIMVAYWPYVPYDQSN
AFR51758_1_pir_________________PPQKAPKEHSWVDFAKKYLNMMDYLGTYFLPFYHSFTPIFQWYHPNINPYQRNEFKFQISFRVPVFRHILWTKGTLYLAYTQTDWFQIYNNPQSAPMRMINFMPELIYVYPINFKPFggNFSEIWIGWQHISNGVGGAqvSAGQRPVFRLVWEKGGLKIMVAYWPYIPYDQSN
AFR51846_1_pir_________________PPQKAPKEHSWVDFAKKYLNMMDYLGTYFLPFYHSFTPIFQWYHPNINPYQRNEFKFQISFRVPVFRHILWTKGTLYLAYTQTDWFQIYNNPQSAPMRMINFMPELIYVYPINFKPFggNFSEIWIGWQHISNGVGGAqvSAGQRPVFRLVWEKGGLKIMVAYWPYIPYDQSN
WP_000836607_1_pir_____________PPQKAPKEHSWVDFAKKYLNMMDYLGTYFLPFYHSFTPIFQWYHPNINPYQRNEFKFQISFRVPVFRHILWTKGTLYLAYTQTNWFQIYNDPQSAPMRMINFMPELIYVYPINFKPFggNFSEIWIGWQHISNGVGGAqvSAGQRPVFRLVWEKGGLKIMVAYWPYVPYDQSN
WP_000836617_1_pir_____________PPQKAPKEHSWVDFAKKYLNMMDYLGTYFLPFYHSFTPIFQWYHPNINPYQRNEFKFQISFRVPVFRHILWTKGTLYLAYTQTNWFQIYNDPQSAPMRMINFMPELIYVYPINFKPFggNFSEIWIGWQHISNGVGGAqvSAGQRPVFRLVWEKGGLKIMVAYWPYVPYDQSN
WP_000836607_1_pir_____________PPQKAPKEHSWVDFAKKYLNMMDYLGTYFLPFYHSFTPIFQWYHPNINPYQRNEFKFQISFRVPVFRHILWTKGTLYLAYTQTNWFQIYNDPQSAPMRMINFMPELIYVYPINFKPFggNFSEIWIGWQHISNGVGGAqvSAGQRPVFRLVWEKGGLKIMVAYWPYVPYDQSN
WP_000836604_1_pir_____________PPQKAPKEHSWVDFAKKYLNMMDYLGTYFLPFYHSFTPIFQWYHPNINPYQRNEFKFQISFRVPVFRHILWTKGTLYLAYTQTDWFQIYNDPQSAPMRMMNFMPELIYVYPINFKPFggNFSEIWIGWQHISNGVGGAqvSAGQRPVFRLVWEKGGLKIMVAYWPYIPYDQSN
WP_000836602_1_pir_____________PPQKAPKEHSWVDFAKKYLNMMDYLGTYFLPFYHSFTPIFQWYHPNINPYQRNEFKFQISFRVPVFRHILWTKGTLYLAYTQTDWFQIYNDPQSAPMRMINFMPELIYVYPINFKPFggNFSEIWIGWQHISNGVGGAqvSAGQRPVFRLVWEKGGLKIMVAYWPYVPYDQSN
WP_001916655_1_pir_____________PPQKAPKEHSWVDFAKKYLNMMDYLGTYFLPFYHSFTPIFQWYHPNINPYQRNEFKFQISFRVPVFRHILWTKGTLYLAYTQTDWFQIYNDPQSAPMRMINFMPELIYVYPINFKPFggNFSEIWIGWQHISNGVGGAqvSAGQRPVFRLVWEKGGLKIMVAYWPYVPYDQSN
WP_000836602_1_pir_____________PPQKAPKEHSWVDFAKKYLNMMDYLGTYFLPFYHSFTPIFQWYHPNINPYQRNEFKFQISFRVPVFRHILWTKGTLYLAYTQTDWFQIYNDPQSAPMRMINFMPELIYVYPINFKPFggNFSEIWIGWQHISNGVGGAqvSAGQRPVFRLVWEKGGLKIMVAYWPYVPYDQSN
WP_000843904_1_pir_____________APQKAPKEHSWVDFAKKYLNMMDYLGTYFLPFYHSFTPIFQWYHPNINPYQRNEFKFQISFRVPVFRHILWTKGTLYLAYTQTNWFQIYNNPQSAPMRMINFMPELIYVYPINFKPFggNFSEIWIGWQHISNGVGGAqvSAGQRPVFRLVWEKGGLKIMVAYWPYIPYDQSN
WP_033777655_1_pir_____________APQKAPKEHSWVDFAKKYLNMMDYLGTYFLPFYHSFTPIFQWYHPNINPYQRNEFKFQISFRVPVFRHILWTKGTLYLAYTQTNWFQIYNNPQSAPMRMINFMPELIYVYPINFKPFggNFSEIWIGWQHISNGVGGAqvSAGQRPVFRLVWEKGGLKIMVAYWPYIPYDQSN
AFR51722_1_pir_________________PPQKAPKEHSWVDFAKKYLNMMDYLGTYFLPFYHSFTPIFQWYHPNINPYQRNEFKFQISFRVPVFRHILWTKGTLYLAYTQTNWFQIYNNPQSAPMRMMNFMPELIYVYPINFKPFggNFSEIWIGWQHISNGVGGAqvSAGQRPVFRLVWEKGGLKIMVAYWPYIPYDQSN
WP_001975437_1_pir_____________APQKAPKEHSWVDFAKKYLNMMDYLGTYFLPFYHSFTPIFQWYHPNINPYQRNEFKFQISFRVPVFRHILWTKGTLYLAYTQTNWFQIYNDPQSAPMRMINFMPELIYVYPINFKPFggNFSEIWIGWQHISNGVGGAqvSAGQRPVFRLVWEKGGLKIMVAYWPYVPYDQSN
WP_001946606_1_pir_____________PPQKAPKEHSWVDFAKKYLNMMDYLGTYFLPFYHSFTPIFQWYHPNINPYQRNEFKFQISFRVPVFRHILWTKGTLYLAYTQTNWFQIYNNPQSAPMRMINFMPELIYVYPINFKPFggNFSEIWIGWQHISNGVGGAqvSAGQRPVFRLVWEKGGLKIMVAYWPYVPYDQSN
WP_017280560_1_pir_____________PPQKAPKEHSWVDFAKKYLNMMDYLGTYFLPFYHSFTPIFQWYHPNINPYQRNEFKFQISFRVPVFRHILWTKGTLYLAYTQTNWFQIYNNPQSAPMRMINFMPELIYVYPINFKPFggNFSEIWIGWQHISNGVGGAqvSAGQRPVFRLVWEKGGLKIMVAYWPYVPYDQSN
WP_000836612_1_pir_____________PPQKAPKEHSWVDFAKKYLNMMDYLGTYFLPFYHSFTPIFQWYHPNINPYQRNEFKFQISFRVPVFRHILWTKGTLYLAYTQTNWFQIYNDPQSAPMRMINFMPELIYVYPINFKPFggNFSEIWIGWQHISNGVGGAqvNAGQRPVFRLVWEKGGLKIMVAYWPYVPYDQSN
WP_000843891_1_pir_____________PPQKAPKEHSWVDFAKKYLNMMDYLGTYFLPFYHSFTPIFQWYHPNINPYQRNEFKFQISFRVPVFRHILWTKGTLYLAYTQTNWFQIYNDPQSAPMRMINFMPELIYVYPINFKPFggNFSEIWIGWQHISNGVGGAqvNAGQRPVFRLVWEKGGLKIMVAYWPYVPYDQSN
AFR51869_1_pir_________________PPQKAPKEHSWVDFAKKYLNMMDYLGTYFLPFYHSFTPIFQWYHPNINPYQRNEFKFQISFRVPVFRHILWTKGTLYLAYTQTDWFQIYNDPQSAPMRMINFMPELIYVYPINFKPFggNFSEIWIGWQHISNGVGGAqvSAGQRPVFRLVWEKGGLKIMVAYWPYIPYDQSN
AFR51770_1_pir_________________PPQKAPKEHSWVDFAKKYLNMMDYLGTYFLPFYHSFTPIFQWYHPNINPYQRNEFKFQISFRVPVFRHILWTKGTLYLAYTQTNWFQIYNDPQSAPMRMMNFMPELIYVYPINFKPFggNFSEIWIGWQHISNGVGGAqvSAGQRPVFRLVWEKGGLKIMVAYWPYVPYDQSN
AFR51805_1_pir_________________PPQKAPKEHSWVDFAKKYLNMMDYLGTYFLPFYHSFTPIFQWYHPNINPYQRNEFKFQISFRVPVFRHILWTKGTLYLAYTQTNWFQIYNDPQSAPMRMMNFMPELIYVYPINFKPFggNFSEIWIGWQHISNGVGGAqvSAGQRPVFRLVWEKGGLKIMVAYWPYVPYDQSN
AFR51807_1_pir_________________PPQKAPKEHSWVDFAKKYLNMMDYLGTYFLPFYHSFTPIFQWYHPNINPYQRNEFKFQISFRVPVFRHILWTKGTLYLAYTQTNWFQIYNDPQSAPMRMMNFMPELIYVYPINFKPFggNFSEIWIGWQHISNGVGGAqvSAGQRPVFRLVWEKGGLKIMVAYWPYVPYDQSN
AFR51812_1_pir_________________PPQKAPKEHSWVDFAKKYLNMMDYLGTYFLPFYHSFTPIFQWYHPNINPYQRNEFKFQISFRVPVFRHILWTKGTLYLAYTQTNWFQIYNDPQSAPMRMMNFMPELIYVYPINFKPFggNFSEIWIGWQHISNGVGGAqvSAGQRPVFRLVWEKGGLKIMVAYWPYVPYDQSN
AFR51844_1_pir_________________PPQKAPKEHSWVDFAKKYLNMMDYLGTYFLPFYHSFTPIFQWYHPNINPYQRNEFKFQISFRVPVFRHILWTKGTLYLAYTQTNWFQIYNDPQSAPMRMMNFMPELIYVYPINFKPFggNFSEIWIGWQHISNGVGGAqvSAGQRPVFRLVWEKGGLKIMVAYWPYVPYDQSN
AFR51859_1_pir_________________PPQKAPKEHSWVDFAKKYLNMMDYLGTYFLPFYHSFTPIFQWYHPNINPYQRNEFKFQISFRVPVFRHILWTKGTLYLAYTQTNWFQIYNDPQSAPMRMMNFMPELIYVYPINFKPFggNFSEIWIGWQHISNGVGGAqvSAGQRPVFRLVWEKGGLKIMVAYWPYVPYDQSN
WP_000836089_1_pir_____________PPQKAPKEHSWVDFAKKYLNMMDYLGTYFLPFYHSFTPIFQWYHPNINPYQRNEFKFQISFRVPVFRHILWTKGTLYLAYTQTNWFQIYNDPQSAPMRMMNFMPELIYVYPINFKPFggNFSEIWIGWQHISNGVGGAqvSAGQRPVFRLVWEKGGLKIMVAYWPYVPYDQSN
WP_000836610_1_pir_____________PPQKAPKEHSWVDFAKKYLNMMDYLGTYFLPFYHSFTPIFQWYHPNINPYQRNEFKFQISFRVPVFRHILWTKGTLYLAYTQTNWFQIYNDPQSAPMRMMNFMPELIYVYPINFKPFggNFSEIWIGWQHISNGVGGAqvSAGQRPVFRLVWEKGGLKIMVAYWPYVPYDQSN
WP_001922279_1_pir_____________PPQKAPKEHSWVDFAKKYLNMMDYLGTYFLPFYHSFTPIFQWYHPNINPYQRNEFKFQISFRVPVFRHILWTKGTLYLAYTQTNWFQIYNDPQSAPMRMMNFMPELIYVYPINFKPFggNFSEIWIGWQHISNGVGGAqvSAGQRPVFRLVWEKGGLKIMVAYWPYVPYDQSN
WP_022576797_1_pir_____________PPQKAPKEHSWVDFAKKYLNMMDYLGTYFLPFYHSFTPIFQWYHPNINPYQRNEFKFQISFRVPVFRHILWTKGTLYLAYTQTNWFQIYNDPQSAPMRMMNFMPELIYVYPINFKPFggNFSEIWIGWQHISNGVGGAqvSAGQRPVFRLVWEKGGLKIMVAYWPYVPYDQSN
WP_023592070_1_pir_____________PPQKAPKEHSWVDFAKKYLNMMDYLGTYFLPFYHSFTPIFQWYHPNINPYQRNEFKFQISFRVPVFRHILWTKGTLYLAYTQTNWFQIYNDPQSAPMRMMNFMPELIYVYPINFKPFggNFSEIWIGWQHISNGVGGAqvSAGQRPVFRLVWEKGGLKIMVAYWPYVPYDQSN
```

```
WP_033584817_1_pir_____________PPQKAPKEHSWVDFAKKYLNMMDYLGTYFLPFYHSFTPIFQWYHPNINPYQRNEFKFQISFRVPVFRHILWTKGTLYLAYTQTNWFQIYNDPQSAPMRMMNFMPELIYVYPINFKPFggNFSEIWIGWQHISNGVGGAqvSAGQRPVFRLVWEKGGLKIMVAYWPYVPYDQSNF
WP_033584817_1_pir_____________PPQKAPKEHSWVDFAKKYLNMMDYLGTYFLPFYHSFTPIFQWYHPNINPYQRNEFKFQISFRVPVFRHILWTKGTLYLAYTQTNWFQIYNDPQSAPMRMMNFMPELIYVYPINFKPFggNFSEIWIGWQHISNGVGGAqvSAGQRPVFRLVWEKGGLKIMVAYWPYVPYDQSNF
WP_015085666_1_pir_____________PPQKAPKEHSWVDFAKKYLNMMDYLGTYFLPFYHSFTPIFQWYHPNINPYQRNEFKFQISFRVPVFRHILWTKGTLYLAYTQTNWFQIYNDPQSAPMRMINFMPELIYVYPINFEPFggKFSEIWIGWQHISNGVGGAqvSAGQRPVFRLVWEKGGLKIMVAYWPYVPYDQSNF
WP_015085666_1_pir_____________PPQKAPKEHSWVDFAKKYLNMMDYLGTYFLPFYHSFTPIFQWYHPNINPYQRNEFKFQISFRVPVFRHILWTKGTLYLAYTQTNWFQIYNDPQSAPMRMINFMPELIYVYPINFEPFggKFSEIWIGWQHISNGVGGAqvSAGQRPVFRLVWEKGGLKIMVAYWPYVPYDQSNF
WP_042636641_1_pir_____________APQKAPKEHSWVDFAKKYLNMMDYLGTYFLPFYHSFTPIFQWYHPNINPYQRNEFKFQISFRVPVFRHILWTKGTLYLAYTQTNWFQIYNDPQSAPMRMINFMPELIYVYPINFKPFggNFSEIWIGWQHISNGVGGAqvSAGQRPVFRLVWEKGGLKIMVAYWPYIPYDQSNF
WP_000836146_1_pir_____________PPQKAPKEHSWVDFAKKYLNMMDYLGTYFLPFYHSFTPIFQWYHPNINPYQRNEFKFQISFRVPVFRHILWTKGTLYLAYTQTNWFQIYNNPQSAPMRMINFMPELIYVYPINFKPFggNFSEIWIGWQHISNGVGGAqvSAGQRPVFRLVWEKGGLKIMVAYWPYVPYDQSNF
WP_000843895_1_pir_____________PPQKAPKEHSWVDFAKKYLNMMDYLGTYFLPFYHSFTPIFQWYHPNINPYQRNEFKFQISFRVPVFRHILWTKGTLYLAYTQTNWFQIYNNPQSAPMRMINFMPELIYVYPINFKPFggNFSEIWIGWQHISNGVGGAqvSAGQRPVFRLVWEKGGLKIMVAYWPYIPYDQSNF
WP_000843896_1_pir_____________PPQKAPKEHSWVDFAKKYLNMMDYLGTYFLPFYHSFTPIFQWYHPNINPYQRNEFKFQISFRVPVFRHILWTKGTLYLAYTQTNWFQIYNNPQSAPMRMINFMPELIYVYPINFKPFggNFSEIWIGWQHISNGVGGAqvSAGQRPVFRLVWEKGGLKIMVAYWPYIPYDQSNF
WP_033593640_1_pir_____________PPQKAPKEHSWVDFAKKYLNMMDYLGTYFLPFYHSFTPIFQWYHPNINPYQRNEFKFQISFRVPVFRHILWTKGTLYLAYTQTNWFQIYNNPQSAPMRMINFMPELIYVYPINFKPFggNFSEIWIGWQHISNGVGGAqvSAGQRPVFRLVWEKGGLKIMVAYWPYIPYDQSNF
WP_033621859_1_pir_____________PPQKAPKEHSWVDFAKKYLNMMDYLGTYFLPFYHSFTPIFQWYHPNINPYQRNEFKFQISFRVPVFRHILWTKGTLYLAYTQTNWFQIYNNPQSAPMRMINFMPELIYVYPINFKPFggNFSEIWIGWQHISNGVGGAqvSAGQRPVFRLVWEKGGLKIMVAYWPYIPYDQSNF
WP_033746958_1_pir_____________PPQKAPKEHSWVDFAKKYLNMMDYLGTYFLPFYHSFTPIFQWYHPNINPYQRNEFKFQISFRVPVFRHILWTKGTLYLAYTQTNWFQIYNNPQSAPMRMINFMPELIYVYPINFKPFggNFSEIWIGWQHISNGVGGAqvSAGQRPVFRLVWEKGGLKIMVAYWPYIPYDQSNF
WP_001928867_1_pir_____________PPQKAPKEHSWVDFAKKYLNMMDYLGTYFLPFYHSFTPIFQWYHPNINPYQRNEFKFQISFRVPVFRHILWTKGTLYLAYTQTNWFQIYNDPQSAPMRMINFMPELIYVYPINFKPFggNFSEIWIGWQHISNGVGGAqvSAGQRPVFRLVWEKGGLKIMVAYWPYVPYDQFNF
AFR51792_1_pir_________________PPQKAPKEHSWVDFAKKYLNMMDYLGTYFLPFYHSFTPIFQWYHPNINPYQRNEFKFQISFRVPVFRHILWTKGTLYLAYTQTDWFQIYNNPQSAPMRMINFMPELIYVYPINFKPFggNFSEIWIGWQHISNGVGGAqvSAGQRPVFRLVWEKGGLKIMVAYWPYVPYDQSNF
AFR51798_1_pir_________________PPQKAPKEHSWVDFAKKYLNMMDYLGTYFLPFYHSFTPIFQWYHPNINPYQRNEFKFQISFRVPVFRHILWTKGTLYLAYTQTDWFQIYNNPQSAPMRMINFMPELIYVYPINFKPFggNFSEIWIGWQHISNGVGGAqvSAGQRPVFRLVWEKGGLKIMVAYWPYVPYDQSNF
AFR51934_1_pir_________________PPQKAPKEHSWVDFAKKYLNMMDYLGTYFLPFYHSFTPIFQWYHPNINPYQRNEFKFQISFRVPVFRHILWTKGTLYLAYTQTDWFQIYNNPQSAPMRMINFMPELIYVYPINFKPFggNFSEIWIGWQHISNGVGGAqvSAGQRPVFRLVWEKGGLKIMVAYWPYVPYDQSNF
WP_000836118_1_pir_____________PPQKAPKEHSWVDFAKKYLNMMDYLGTYFLPFYHSFTPIFQWYHPNINPYQRNEFKFQISFRVPVFRHILWTKGTLYLAYTQTDWFQIYNNPQSAPMRMINFMPELIYVYPINFKPFggNFSEIWIGWQHISNGVGGAqvSAGQRPVFRLVWEKGGLKIMVAYWPYVPYDQSNF
WP_021310308_1_pir_____________PPQKAPKEHSWVDFAKKYLNMMDYLGTYFLPFYHSFTPIFQWYHPNINPYQRNEFKFQISFRVPVFRHILWTKGTLYLAYTQTDWFQIYNNPQSAPMRMINFMPELIYVYPINFKPFggNFSEIWIGWQHISNGVGGAqvSAGQRPVFRLVWEKGGLKIMVAYWPYVPYDQSNF
WP_050844251_1_pir_____________PPQKAPKEHSWVDFAKKYLNMMDYLGTYFLPFYHSFTPIFQWYHPNINPYQRNEFKFQISFRVPVFRHILWTKGTLYLAYTQTDWFQIYNNPQSAPMRMINFMPELIYVYPINFKPFggNFSEIWIGWQHISNGVGGAqvSAGQRPVFRLVWEKGGLKIMVAYWPYVPYDQSNF
WP_060758518_1_pir_____________PPQKAPKEHSWVDFAKKYLNMMDYLGTYFLPFYHSFTPIFQWYHPNINPYQRNEFKFQISFRVPVFRHILWTKGTLYLAYTQTDWFQIYNNPQSAPMRMINFMPELIYVYPINFKPFggNFSEIWIGWQHISNGVGGAqvSAGQRPVFRLVWEKGGLKIMVAYWPYVPYDQSNF
WP_000836144_1_pir_____________PPQKAPKEHSWVDFAKKYLNMMDYLGTYFLPFYHSFTPIFQWYHPNINPYQRNEFKFQISFRVPVFRHILWTKGTLYLAYTQTNWFQIYNNPQSAPMRMINFMPELIYVYPINFKPFggNFSEIWIGWQHISNGVGGTqvSAGQRPVFRLVWEKGGLKIMVAYWPYIPYDQSNF
AFR51718_1_pir_________________PPQKAPKEHSWVDFAKKYLNMMDYLGTYFLPFYHSFTPIFQWYHPNINPYQRNEFKFQISFRVPVFRHILWTKGTLYLAYTQTDWFQIYNDPQSAPMRMMNFMPELIYVYPINFKPFggNFSEIWIGWQHISNGVGGAqvSAGQRPVFRLVWEKGGLKIMVAYWPYVPYDQSNF
AFR51753_1_pir_________________PPQKAPKEHSWVDFAKKYLNMMDYLGTYFLPFYHSFTPIFQWYHPNINPYQRNEFKFQISFRVPVFRHILWTKGTLYLAYTQTDWFQIYNDPQSAPMRMMNFMPELIYVYPINFKPFggNFSEIWIGWQHISNGVGGAqvSAGQRPVFRLVWEKGGLKIMVAYWPYVPYDQSNF
AFR51811_1_pir_________________PPQKAPKEHSWVDFAKKYLNMMDYLGTYFLPFYHSFTPIFQWYHPNINPYQRNEFKFQISFRVPVFRHILWTKGTLYLAYTQTDWFQIYNDPQSAPMRMMNFMPELIYVYPINFKPFggNFSEIWIGWQHISNGVGGAqvSAGQRPVFRLVWEKGGLKIMVAYWPYVPYDQSNF
WP_000836603_1_pir_____________PPQKAPKEHSWVDFAKKYLNMMDYLGTYFLPFYHSFTPIFQWYHPNINPYQRNEFKFQISFRVPVFRHILWTKGTLYLAYTQTDWFQIYNDPQSAPMRMMNFMPELIYVYPINFKPFggNFSEIWIGWQHISNGVGGAqvSAGQRPVFRLVWEKGGLKIMVAYWPYVPYDQSNF
WP_000836605_1_pir_____________PPQKAPKEHSWVDFAKKYLNMMDYLGTYFLPFYHSFTPIFQWYHPNINPYQRNEFKFQISFRVPVFRHILWTKGTLYLAYTQTDWFQIYNDPQSAPMRMMNFMPELIYVYPINFKPFggNFSEIWIGWQHISNGVGGAqvSAGQRPVFRLVWEKGGLKIMVAYWPYVPYDQSNF
WP_033741650_1_pir_____________PPQKAPKEHSWVDFAKKYLNMMDYLGTYFLPFYHSFTPIFQWYHPNINPYQRNEFKFQISFRVPVFRHILWTKGTLYLAYTQTDWFQIYNDPQSAPMRMMNFMPELIYVYPINFKPFggNFSEIWIGWQHISNGVGGAqvSAGQRPVFRLVWEKGGLKIMVAYWPYVPYDQSNF
WP_050841669_1_pir_____________PPQKAPKEHSWVDFAKKYLNMMDYLGTYFLPFYHSFTPIFQWYHPNINPYQRNEFKFQISFRVPVFRHILWTKGTLYLAYTQTDWFQIYNDPQSAPMRMMNFMPELIYVYPINFKPFggNFSEIWIGWQHISNGVGGAqvSAGQRPVFRLVWEKGGLKIMVAYWPYVPYDQSNF
WP_060474740_1_pir_____________PPQKAPKEHSWVDFAKKYLNMMDYLGTYFLPFYHSFTPIFQWYHPNINPYQRNEFKFQISFRVPVFRHILWTKGTLYLAYTQTDWFQIYNDPQSAPMRMMNFMPELIYVYPINFKPFggNFSEIWIGWQHISNGVGGAqvSAGQRPVFRLVWEKGGLKIMVAYWPYVPYDQSNF
AFR51723_1_pir_________________PPQKAPKEHSWVDFAKKYLNMMDYLGTYFLPFYHSFTPIFQWYHPNINPYQRNEFKFQISFRVPVFRHILWTKGTLYLAYTQTNWFQIYNDPQSAPMRMINFMPELIYVYPINFKPFggNFSEIWIGWQHISNGVGGAqvSAGQRPVFRLVWEKGGLKIMVAYWPYVPYDQSNF
AFR51920_1_pir_________________PPQKAPKEHSWVDFAKKYLNMMDYLGTYFLPFYHSFTPIFQWYHPNINPYQRNEFKFQISFRVPVFRHILWTKGTLYLAYTQTNWFQIYNDPQSAPMRMINFMPELIYVYPINFKPFggNFSEIWIGWQHISNGVGGAqvSAGQRPVFRLVWEKGGLKIMVAYWPYVPYDQSNF
WP_000836122_1_pir_____________PPQKAPKEHSWVDFAKKYLNMMDYLGTYFLPFYHSFTPIFQWYHPNINPYQRNEFKFQISFRVPVFRHILWTKGTLYLAYTQTNWFQIYNDPQSAPMRMINFMPELIYVYPINFKPFggNFSEIWIGWQHISNGVGGAqvSAGQRPVFRLVWEKGGLKIMVAYWPYVPYDQSNF
WP_000836148_1_pir_____________PPQKAPKEHSWVDFAKKYLNMMDYLGTYFLPFYHSFTPIFQWYHPNINPYQRNEFKFQISFRVPVFRHILWTKGTLYLAYTQTNWFQIYNDPQSAPMRMINFMPELIYVYPINFKPFggNFSEIWIGWQHISNGVGGAqvSAGQRPVFRLVWEKGGLKIMVAYWPYVPYDQSNF
WP_000843886_1_pir_____________PPQKAPKEHSWVDFAKKYLNMMDYLGTYFLPFYHSFTPIFQWYHPNINPYQRNEFKFQISFRVPVFRHILWTKGTLYLAYTQTNWFQIYNDPQSAPMRMINFMPELIYVYPINFKPFggNFSEIWIGWQHISNGVGGAqvSAGQRPVFRLVWEKGGLKIMVAYWPYVPYDQSNF
WP_000843887_1_pir_____________PPQKAPKEHSWVDFAKKYLNMMDYLGTYFLPFYHSFTPIFQWYHPNINPYQRNEFKFQISFRVPVFRHILWTKGTLYLAYTQTNWFQIYNDPQSAPMRMINFMPELIYVYPINFKPFggNFSEIWIGWQHISNGVGGAqvSAGQRPVFRLVWEKGGLKIMVAYWPYVPYDQSNF
WP_000843889_1_pir_____________PPQKAPKEHSWVDFAKKYLNMMDYLGTYFLPFYHSFTPIFQWYHPNINPYQRNEFKFQISFRVPVFRHILWTKGTLYLAYTQTNWFQIYNDPQSAPMRMINFMPELIYVYPINFKPFggNFSEIWIGWQHISNGVGGAqvSAGQRPVFRLVWEKGGLKIMVAYWPYVPYDQSNF
WP_000843890_1_pir_____________PPQKAPKEHSWVDFAKKYLNMMDYLGTYFLPFYHSFTPIFQWYHPNINPYQRNEFKFQISFRVPVFRHILWTKGTLYLAYTQTNWFQIYNDPQSAPMRMINFMPELIYVYPINFKPFggNFSEIWIGWQHISNGVGGAqvSAGQRPVFRLVWEKGGLKIMVAYWPYVPYDQSNF
WP_000843900_1_pir_____________PPQKAPKEHSWVDFAKKYLNMMDYLGTYFLPFYHSFTPIFQWYHPNINPYQRNEFKFQISFRVPVFRHILWTKGTLYLAYTQTNWFQIYNDPQSAPMRMINFMPELIYVYPINFKPFggNFSEIWIGWQHISNGVGGAqvSAGQRPVFRLVWEKGGLKIMVAYWPYVPYDQSNF
WP_000843902_1_pir_____________PPQKAPKEHSWVDFAKKYLNMMDYLGTYFLPFYHSFTPIFQWYHPNINPYQRNEFKFQISFRVPVFRHILWTKGTLYLAYTQTNWFQIYNDPQSAPMRMINFMPELIYVYPINFKPFggNFSEIWIGWQHISNGVGGAqvSAGQRPVFRLVWEKGGLKIMVAYWPYVPYDQSNF
WP_001931129_1_pir_____________PPQKAPKEHSWVDFAKKYLNMMDYLGTYFLPFYHSFTPIFQWYHPNINPYQRNEFKFQISFRVPVFRHILWTKGTLYLAYTQTNWFQIYNDPQSAPMRMINFMPELIYVYPINFKPFggNFSEIWIGWQHISNGVGGAqvSAGQRPVFRLVWEKGGLKIMVAYWPYVPYDQSNF
WP_001936560_1_pir_____________PPQKAPKEHSWVDFAKKYLNMMDYLGTYFLPFYHSFTPIFQWYHPNINPYQRNEFKFQISFRVPVFRHILWTKGTLYLAYTQTNWFQIYNDPQSAPMRMINFMPELIYVYPINFKPFggNFSEIWIGWQHISNGVGGAqvSAGQRPVFRLVWEKGGLKIMVAYWPYVPYDQSNF
WP_001939617_1_pir_____________PPQKAPKEHSWVDFAKKYLNMMDYLGTYFLPFYHSFTPIFQWYHPNINPYQRNEFKFQISFRVPVFRHILWTKGTLYLAYTQTNWFQIYNDPQSAPMRMINFMPELIYVYPINFKPFggNFSEIWIGWQHISNGVGGAqvSAGQRPVFRLVWEKGGLKIMVAYWPYVPYDQSNF
WP_001940719_1_pir_____________PPQKAPKEHSWVDFAKKYLNMMDYLGTYFLPFYHSFTPIFQWYHPNINPYQRNEFKFQISFRVPVFRHILWTKGTLYLAYTQTNWFQIYNDPQSAPMRMINFMPELIYVYPINFKPFggNFSEIWIGWQHISNGVGGAqvSAGQRPVFRLVWEKGGLKIMVAYWPYVPYDQSNF
WP_001944576_1_pir_____________PPQKAPKEHSWVDFAKKYLNMMDYLGTYFLPFYHSFTPIFQWYHPNINPYQRNEFKFQISFRVPVFRHILWTKGTLYLAYTQTNWFQIYNDPQSAPMRMINFMPELIYVYPINFKPFggNFSEIWIGWQHISNGVGGAqvSAGQRPVFRLVWEKGGLKIMVAYWPYVPYDQSNF
WP_001948558_1_pir_____________PPQKAPKEHSWVDFAKKYLNMMDYLGTYFLPFYHSFTPIFQWYHPNINPYQRNEFKFQISFRVPVFRHILWTKGTLYLAYTQTNWFQIYNDPQSAPMRMINFMPELIYVYPINFKPFggNFSEIWIGWQHISNGVGGAqvSAGQRPVFRLVWEKGGLKIMVAYWPYVPYDQSNF
WP_001958394_1_pir_____________PPQKAPKEHSWVDFAKKYLNMMDYLGTYFLPFYHSFTPIFQWYHPNINPYQRNEFKFQISFRVPVFRHILWTKGTLYLAYTQTNWFQIYNDPQSAPMRMINFMPELIYVYPINFKPFggNFSEIWIGWQHISNGVGGAqvSAGQRPVFRLVWEKGGLKIMVAYWPYVPYDQSNF
WP_033745716_1_pir_____________PPQKAPKEHSWVDFAKKYLNMMDYLGTYFLPFYHSFTPIFQWYHPNINPYQRNEFKFQISFRVPVFRHILWTKGTLYLAYTQTNWFQIYNDPQSAPMRMINFMPELIYVYPINFKPFggNFSEIWIGWQHISNGVGGAqvSAGQRPVFRLVWEKGGLKIMVAYWPYVPYDQSNF
WP_033768136_1_pir_____________PPQKAPKEHSWVDFAKKYLNMMDYLGTYFLPFYHSFTPIFQWYHPNINPYQRNEFKFQISFRVPVFRHILWTKGTLYLAYTQTNWFQIYNDPQSAPMRMINFMPELIYVYPINFKPFggNFSEIWIGWQHISNGVGGAqvSAGQRPVFRLVWEKGGLKIMVAYWPYVPYDQSNF
WP_060473285_1_pir_____________PPQKAPKEHSWVDFAKKYLNMMDYLGTYFLPFYHSFTPIFQWYHPNINPYQRNEFKFQISFRVPVFRHILWTKGTLYLAYTQTNWFQIYNDPQSAPMRMINFMPELIYVYPINFKPFggNFSEIWIGWQHISNGVGGAqvSAGQRPVFRLVWEKGGLKIMVAYWPYVPYDQSNF
WP_064780878_1_pir_____________PPQKAPKEHSWVDFAKKYLNMMDYLGTYFLPFYHSFTPIFQWYHPNINPYQRNEFKFQISFRVPVFRHILWTKGTLYLAYTQTNWFQIYNDPQSAPMRMINFMPELIYVYPINFKPFggNFSEIWIGWQHISNGVGGAqvSAGQRPVFRLVWEKGGLKIMVAYWPYVPYDQSNF
WP_064792534_1_pir_____________PPQKAPKEHSWVDFAKKYLNMMDYLGTYFLPFYHSFTPIFQWYHPNINPYQRNEFKFQISFRVPVFRHILWTKGTLYLAYTQTNWFQIYNDPQSAPMRMINFMPELIYVYPINFKPFggNFSEIWIGWQHISNGVGGAqvSAGQRPVFRLVWEKGGLKIMVAYWPYVPYDQSNF
EJB86456_1_pir_________________PPQKAPKEHSWVDFAKKYLNMMDYLGTYFLPFYHSFTPIFQWYHPNINPYQRNEFKFQISFRVPVFRHILWTKGTLYLAYTQTNWFQIYNDPQSAPMRMMNFMPELIYVYPINFKPFggNFSEIWIGWQHISNGVGGTqvSAGQRPVFRLVWEKGGLKIMVAYWPYIPYDQSNF
WP_000836090_1_pir_____________PPQKAPKEHSWVDFAKKYLNMMDYLGTYFLPFYHSFTPIFQWYHPNINPYQRNEFKFQISFRVPVFRHILWTKGTLYLAYTQTNWFQIYNDPQSAPMRMMNFMPELIYVYPINFKPFggNFSEIWIGWQHISNGVGGTqvSAGQRPVFRLVWEKGGLKIMVAYWPYIPYDQSNF
EJB86456_1_pir_________________PPQKAPKEHSWVDFAKKYLNMMDYLGTYFLPFYHSFTPIFQWYHPNINPYQRNEFKFQISFRVPVFRHILWTKGTLYLAYTQTNWFQIYNDPQSAPMRMMNFMPELIYVYPINFKPFggNFSEIWIGWQHISNGVGGTqvSAGQRPVFRLVWEKGGLKIMVAYWPYIPYDQSNF
WP_000836609_1_pir_____________PPQKAPKEHSWVDFAKKYLNMMDYLGTYFLPFYHSFTPIFQWYHPNINPYQRNEFKFQISFRVPVFRHILWTKGTLYLAYTQTNWFQIYNDPQSAPMRMMNFMPELIYVYPINFKPFggNFSEIWIGWQHISNGVGGAqvSAGQRPVFRLVWEKGDLKIMVAYWPYVPYDQSNF
WP_039086986_1_pir_____________APQKAPKEHSWVDFAKKYLNMMDYLGTYFLPFYHSFTPIFQWYHPNINPYQRNEFKFQISFRVPVFRHILWTKGTLYLAYTQTNWFQIYNDPQSAPMRMMNFMPELIYVYPINFKPFggNFSEIWIGWQHISNGVGGAqvSAGQRPVFRLVWEKGGLKIMVAYWPYVPYDQSNF
AFR51935_1_pir_________________PPQKAPKEHSWVDFAKKYLNMMDYLGTYFLPFYHSFTPIFQWYHPNINPYQRNEFKFQISFRVPVFRHILWTKGTLYLAYTQTNWFQIYNDPQSAPMRMMNFMPELIYVYPIDFKPFggNFSEIWIGWQHISNGVGGAqvSAGQRPVFRLVWEKGGLKIMVAYWPYVPYDQSNF
WP_025276062_1_pir_____________PPQKAPKEHSWVDFAKKYLNMMDYLGTYFLPFYHSFTPIFQWYHPNINPYQRNEFKFQISFRVPVFRHILWTKGTLYLAYTQTNWFQIYNNPQSAPMRMMNFMPELIYVYPINFKPFggNFSEIWIGWQHISNGVGGAqvSAGQRPVFRLVWEKGGLKIMVAYWPYVPYDQSNF
WP_000836103_1_pir_____________PPQKAPKEHSWVDFAKKYLNMMDYLGTYFLPFYHSFTPIFQWYHPNINPYQRNEFKFQISFRVPVFRHILWTKGTLYLAYTQTNWFQIYNNPQSAPMRMINFMPELIYVYPINFKPFggNFSEIWIGWQHISNGVGGAqvSAVQRPVFRLVWEKGGLKIMVAYWPYVPYDQSNF
WP_000843905_1_pir_____________SPQKAPKEHSWVDFAKKYLNMMDYLGTYFLPFYHSFTPIFQWYHPNINPYQRNEFKFQISFRVPVFRHILWTKGTLYLAYTQTNWFQIYNDPQSAPMRMINFMPELIYVYPINFKPFggNFSEIWIGWQHISNGVGGAqvSAGQRPVFRLVWEKGGLKIMVAYWPYIPYDQSNF
AFR51825_1_pir_________________PPQKAPKEHSWVDFAKKYLNMMDYLGTYFLPFYHSFTPIFQWYHPNINPYQRNEFKFQISFRVPVFRHILWTKGTLYLAYTQTDWFQIYNDPQSAPMRMINFMPELIYVYPINFKPFggNFSEIWIGWQHISNGVGGAqvSAGQRPVFRLVWEKGGLKIMVAYWPYVPYDQSNF
AFR51893_1_pir_________________PPQKAPKEHSWVDFAKKYLNMMDYLGTYFLPFYHSFTPIFQWYHPNINPYQRNEFKFQISFRVPVFRHILWTKGTLYLAYTQTDWFQIYNDPQSAPMRMINFMPELIYVYPINFKPFggNFSEIWIGWQHISNGVGGAqvSAGQRPVFRLVWEKGGLKIMVAYWPYVPYDQSNF
AFR51894_1_pir_________________PPQKAPKEHSWVDFAKKYLNMMDYLGTYFLPFYHSFTPIFQWYHPNINPYQRNEFKFQISFRVPVFRHILWTKGTLYLAYTQTDWFQIYNDPQSAPMRMINFMPELIYVYPINFKPFggNFSEIWIGWQHISNGVGGAqvSAGQRPVFRLVWEKGGLKIMVAYWPYVPYDQSNF
WP_000836106_1_pir_____________PPQKAPKEHSWVDFAKKYLNMMDYLGTYFLPFYHSFTPIFQWYHPNINPYQRNEFKFQISFRVPVFRHILWTKGTLYLAYTQTDWFQIYNDPQSAPMRMINFMPELIYVYPINFKPFggNFSEIWIGWQHISNGVGGAqvSAGQRPVFRLVWEKGGLKIMVAYWPYVPYDQSNF
WP_000836601_1_pir_____________PPQKAPKEHSWVDFAKKYLNMMDYLGTYFLPFYHSFTPIFQWYHPNINPYQRNEFKFQISFRVPVFRHILWTKGTLYLAYTQTDWFQIYNDPQSAPMRMINFMPELIYVYPINFKPFggNFSEIWIGWQHISNGVGGAqvSAGQRPVFRLVWEKGGLKIMVAYWPYVPYDQSNF
WP_000840064_1_pir_____________APQKAPKEHSWVDFAKKYLNMMDYLGTYFLPFYHSFTPIFQWYHPNINPYQRNEFKFQISFRVPVFRHILWTKGTLYLAYTQTNWFQIYNDPQSAPMRMINFMPELIYVYPINFKPFggNFSEIWIGWQHISNGVGGAqvSAGQRPVFRLVWEKGGLKIMVAYWPYVPYDQSNF
WP_001936001_1_pir_____________APQKAPKEHSWVDFAKKYLNMMDYLGTYFLPFYHSFTPIFQWYHPNINPYQRNEFKFQISFRVPVFRHILWTKGTLYLAYTQTNWFQIYNDPQSAPMRMINFMPELIYVYPINFKPFggNFSEIWIGWQHISNGVGGAqvSAGQRPVFRLVWEKGGLKIMVAYWPYVPYDQSNF
WP_001970860_1_pir_____________APQKAPKEHSWVDFAKKYLNMMDYLGTYFLPFYHSFTPIFQWYHPNINPYQRNEFKFQISFRVPVFRHILWTKGTLYLAYTQTNWFQIYNDPQSAPMRMINFMPELIYVYPINFKPFggNFSEIWIGWQHISNGVGGAqvSAGQRPVFRLVWEKGGLKIMVAYWPYVPYDQSNF
WP_020972154_1_pir_____________APQKAPKEHSWVDFAKKYLNMMDYLGTYFLPFYHSFTPIFQWYHPNINPYQRNEFKFQISFRVPVFRHILWTKGTLYLAYTQTNWFQIYNDPQSAPMRMINFMPELIYVYPINFKPFggNFSEIWIGWQHISNGVGGAqvSAGQRPVFRLVWEKGGLKIMVAYWPYVPYDQSNF
WP_021436290_1_pir_____________APQKAPKEHSWVDFAKKYLNMMDYLGTYFLPFYHSFTPIFQWYHPNINPYQRNEFKFQISFRVPVFRHILWTKGTLYLAYTQTNWFQIYNDPQSAPMRMINFMPELIYVYPINFKPFggNFSEIWIGWQHISNGVGGAqvSAGQRPVFRLVWEKGGLKIMVAYWPYVPYDQSNF
WP_033603828_1_pir_____________APQKAPKEHSWVDFAKKYLNMMDYLGTYFLPFYHSFTPIFQWYHPNINPYQRNEFKFQISFRVPVFRHILWTKGTLYLAYTQTNWFQIYNDPQSAPMRMINFMPELIYVYPINFKPFggNFSEIWIGWQHISNGVGGAqvSAGQRPVFRLVWEKGGLKIMVAYWPYVPYDQSNF
WP_033619070_1_pir_____________APQKAPKEHSWVDFAKKYLNMMDYLGTYFLPFYHSFTPIFQWYHPNINPYQRNEFKFQISFRVPVFRHILWTKGTLYLAYTQTNWFQIYNDPQSAPMRMINFMPELIYVYPINFKPFggNFSEIWIGWQHISNGVGGAqvSAGQRPVFRLVWEKGGLKIMVAYWPYVPYDQSNF
WP_033739102_1_pir_____________APQKAPKEHSWVDFAKKYLNMMDYLGTYFLPFYHSFTPIFQWYHPNINPYQRNEFKFQISFRVPVFRHILWTKGTLYLAYTQTNWFQIYNDPQSAPMRMINFMPELIYVYPINFKPFggNFSEIWIGWQHISNGVGGAqvSAGQRPVFRLVWEKGGLKIMVAYWPYVPYDQSNF
WP_033750925_1_pir_____________APQKAPKEHSWVDFAKKYLNMMDYLGTYFLPFYHSFTPIFQWYHPNINPYQRNEFKFQISFRVPVFRHILWTKGTLYLAYTQTNWFQIYNDPQSAPMRMINFMPELIYVYPINFKPFggNFSEIWIGWQHISNGVGGAqvSAGQRPVFRLVWEKGGLKIMVAYWPYVPYDQSNF
```

```
WP_033751964_1_pir  APQKAPKEHSWVDFAKKYLNMMDYLGTYFLPFYHSFTPIFQWYHPNINPYQRNEFKFQISFRVPVFRHILWTKGTLYLAYTQTNWFQIYNDPQSAPMRMINFMPELIYVYPINFKPFggNFSEIWIGWQHISNGVGGAqvSAGQRPVFRLVWEKGGLKIMVAYWPYVPYDQSNF
WP_033764301_1_pir  APQKAPKEHSWVDFAKKYLNMMDYLGTYFLPFYHSFTPIFQWYHPNINPYQRNEFKFQISFRVPVFRHILWTKGTLYLAYTQTNWFQIYNDPQSAPMRMINFMPELIYVYPINFKPFggNFSEIWIGWQHISNGVGGAqvSAGQRPVFRLVWEKGGLKIMVAYWPYVPYDQSNF
WP_033771315_1_pir  APQKAPKEHSWVDFAKKYLNMMDYLGTYFLPFYHSFTPIFQWYHPNINPYQRNEFKFQISFRVPVFRHILWTKGTLYLAYTQTNWFQIYNDPQSAPMRMINFMPELIYVYPINFKPFggNFSEIWIGWQHISNGVGGAqvSAGQRPVFRLVWEKGGLKIMVAYWPYVPYDQSNF
WP_064434433_1_pir  APQKAPKEHSWVDFAKKYLNMMDYLGTYFLPFYHSFTPIFQWYHPNINPYQRNEFKFQISFRVPVFRHILWTKGTLYLAYTQTNWFQIYNDPQSAPMRMINFMPELIYVYPINFKPFggNFSEIWIGWQHISNGVGGAqvSAGQRPVFRLVWEKGGLKIMVAYWPYVPYDQSNF
WP_000836094_1_pir  PPQKAPKEHSWVDFAKKYLNMMDYLGTYFLPFYHSFTPIFQWYHPNINPYQRNEFKFQISFRVPVFRHILWTKGTLYLAYTQTDWFQIYNNPQSAPMRMINFMPELIYVYPINFKPFggNFSEIWIGWQHISNGVGGAqvNAGQRPVFRLVWEKGGLKIMVAYWPYVPYDQSNF
AFR51767_1_pir      PPQKAPKEHSWVDFAKKYLNMMDYLGTYFLPFYHSFTPIFQWYHPNINPYQRNEFKFQISFRVPVFRHILWTKGTLYLAYTQTDWFQIYNNPQSAPMRMMNFMPELIYVYPINFKPFggNFSEIWIGWQHISNGVGGAqvSAGQRPVFRLVWEKGGLKIMVAYWPYVPYDQSNF
WP_001927383_1_pir  PPQKAPKEHSWVDFAKKYLNMMDYLGTYFLPFYHSFTPIFQWYHPNINPYQRNEFKFQISFRVPVFRHILWTKGTLYLAYTQTNWFQIYNNPQSAPMRMINFMPELIYVYPINFKPFggNFSEIWIGWQHISNGVGGAqvSAGQRPVFRLVWEKGGLKIMVAYWPYVPYDQSNF
WP_001979369_1_pir  PPQKAPKEHSWVDFAKKYLNMMDYLGTYFLPFYHSFTPIFQWYHPNINPYQRNEFKFQISFRVPVFRHILWTKGTLYLAYTQTNWFQIYNNPQSAPMRMINFMPELIYVYPINFKPFggNFSEIWIGWQHISNGVGGAqvSAGQRPVFRLVWEKGGLKIMVAYWPYVPYDQSNF
WP_026938708_1_pir  PPQKAPKEHSWVDFAKKYLNMMDYLGTYFLPFYHSFTPIFQWYHPNINPYQRNEFKFQISFRVPVFRHILWTKGTLYLAYTQTNWFQIYNNPQSAPMRMINFMPELIYVYPINFKPFggNFSEIWIGWQHISNGVGGAqvSAGQRPVFRLVWEKGGLKIMVAYWPYVPYDQSNF
AFR51721_1_pir      PPQKAPKEHSWVDFAKKYLNMMDYLGTYFLPFYHSFTPIFQWYHPNINPYQRNEFKFQISFRVPVFRHILWTKGTLYLAYTQTNWFQIYNDPQSAPMRMMNFMPELIYVYPINFKPFggNFSEIWIGWQHISNGVGGAqvSAGQRPVFRLVWEKGGLKIMVAYWPYVPYDQSNF
AFR51725_1_pir      PPQKAPKEHSWVDFAKKYLNMMDYLGTYFLPFYHSFTPIFQWYHPNINPYQRNEFKFQISFRVPVFRHILWTKGTLYLAYTQTNWFQIYNDPQSAPMRMMNFMPELIYVYPINFKPFggNFSEIWIGWQHISNGVGGAqvSAGQRPVFRLVWEKGGLKIMVAYWPYVPYDQSNF
AFR51726_1_pir      PPQKAPKEHSWVDFAKKYLNMMDYLGTYFLPFYHSFTPIFQWYHPNINPYQRNEFKFQISFRVPVFRHILWTKGTLYLAYTQTNWFQIYNDPQSAPMRMMNFMPELIYVYPINFKPFggNFSEIWIGWQHISNGVGGAqvSAGQRPVFRLVWEKGGLKIMVAYWPYVPYDQSNF
AFR51730_1_pir      PPQKAPKEHSWVDFAKKYLNMMDYLGTYFLPFYHSFTPIFQWYHPNINPYQRNEFKFQISFRVPVFRHILWTKGTLYLAYTQTNWFQIYNDPQSAPMRMMNFMPELIYVYPINFKPFggNFSEIWIGWQHISNGVGGAqvSAGQRPVFRLVWEKGGLKIMVAYWPYVPYDQSNF
AFR51769_1_pir      PPQKAPKEHSWVDFAKKYLNMMDYLGTYFLPFYHSFTPIFQWYHPNINPYQRNEFKFQISFRVPVFRHILWTKGTLYLAYTQTNWFQIYNDPQSAPMRMMNFMPELIYVYPINFKPFggNFSEIWIGWQHISNGVGGAqvSAGQRPVFRLVWEKGGLKIMVAYWPYVPYDQSNF
AFR51790_1_pir      PPQKAPKEHSWVDFAKKYLNMMDYLGTYFLPFYHSFTPIFQWYHPNINPYQRNEFKFQISFRVPVFRHILWTKGTLYLAYTQTNWFQIYNDPQSAPMRMMNFMPELIYVYPINFKPFggNFSEIWIGWQHISNGVGGAqvSAGQRPVFRLVWEKGGLKIMVAYWPYVPYDQSNF
AFR51794_1_pir      PPQKAPKEHSWVDFAKKYLNMMDYLGTYFLPFYHSFTPIFQWYHPNINPYQRNEFKFQISFRVPVFRHILWTKGTLYLAYTQTNWFQIYNDPQSAPMRMMNFMPELIYVYPINFKPFggNFSEIWIGWQHISNGVGGAqvSAGQRPVFRLVWEKGGLKIMVAYWPYVPYDQSNF
AFR51801_1_pir      PPQKAPKEHSWVDFAKKYLNMMDYLGTYFLPFYHSFTPIFQWYHPNINPYQRNEFKFQISFRVPVFRHILWTKGTLYLAYTQTNWFQIYNDPQSAPMRMMNFMPELIYVYPINFKPFggNFSEIWIGWQHISNGVGGAqvSAGQRPVFRLVWEKGGLKIMVAYWPYVPYDQSNF
AFR51809_1_pir      PPQKAPKEHSWVDFAKKYLNMMDYLGTYFLPFYHSFTPIFQWYHPNINPYQRNEFKFQISFRVPVFRHILWTKGTLYLAYTQTNWFQIYNDPQSAPMRMMNFMPELIYVYPINFKPFggNFSEIWIGWQHISNGVGGAqvSAGQRPVFRLVWEKGGLKIMVAYWPYVPYDQSNF
AFR51813_1_pir      PPQKAPKEHSWVDFAKKYLNMMDYLGTYFLPFYHSFTPIFQWYHPNINPYQRNEFKFQISFRVPVFRHILWTKGTLYLAYTQTNWFQIYNDPQSAPMRMMNFMPELIYVYPINFKPFggNFSEIWIGWQHISNGVGGAqvSAGQRPVFRLVWEKGGLKIMVAYWPYVPYDQSNF
AFR51841_1_pir      PPQKAPKEHSWVDFAKKYLNMMDYLGTYFLPFYHSFTPIFQWYHPNINPYQRNEFKFQISFRVPVFRHILWTKGTLYLAYTQTNWFQIYNDPQSAPMRMMNFMPELIYVYPINFKPFggNFSEIWIGWQHISNGVGGAqvSAGQRPVFRLVWEKGGLKIMVAYWPYVPYDQSNF
AFR51851_1_pir      PPQKAPKEHSWVDFAKKYLNMMDYLGTYFLPFYHSFTPIFQWYHPNINPYQRNEFKFQISFRVPVFRHILWTKGTLYLAYTQTNWFQIYNDPQSAPMRMMNFMPELIYVYPINFKPFggNFSEIWIGWQHISNGVGGAqvSAGQRPVFRLVWEKGGLKIMVAYWPYVPYDQSNF
AFR51852_1_pir      PPQKAPKEHSWVDFAKKYLNMMDYLGTYFLPFYHSFTPIFQWYHPNINPYQRNEFKFQISFRVPVFRHILWTKGTLYLAYTQTNWFQIYNDPQSAPMRMMNFMPELIYVYPINFKPFggNFSEIWIGWQHISNGVGGAqvSAGQRPVFRLVWEKGGLKIMVAYWPYVPYDQSNF
AFR51853_1_pir      PPQKAPKEHSWVDFAKKYLNMMDYLGTYFLPFYHSFTPIFQWYHPNINPYQRNEFKFQISFRVPVFRHILWTKGTLYLAYTQTNWFQIYNDPQSAPMRMMNFMPELIYVYPINFKPFggNFSEIWIGWQHISNGVGGAqvSAGQRPVFRLVWEKGGLKIMVAYWPYVPYDQSNF
AFR51864_1_pir      PPQKAPKEHSWVDFAKKYLNMMDYLGTYFLPFYHSFTPIFQWYHPNINPYQRNEFKFQISFRVPVFRHILWTKGTLYLAYTQTNWFQIYNDPQSAPMRMMNFMPELIYVYPINFKPFggNFSEIWIGWQHISNGVGGAqvSAGQRPVFRLVWEKGGLKIMVAYWPYVPYDQSNF
AFR51871_1_pir      PPQKAPKEHSWVDFAKKYLNMMDYLGTYFLPFYHSFTPIFQWYHPNINPYQRNEFKFQISFRVPVFRHILWTKGTLYLAYTQTNWFQIYNDPQSAPMRMMNFMPELIYVYPINFKPFggNFSEIWIGWQHISNGVGGAqvSAGQRPVFRLVWEKGGLKIMVAYWPYVPYDQSNF
AFR51927_1_pir      PPQKAPKEHSWVDFAKKYLNMMDYLGTYFLPFYHSFTPIFQWYHPNINPYQRNEFKFQISFRVPVFRHILWTKGTLYLAYTQTNWFQIYNDPQSAPMRMMNFMPELIYVYPINFKPFggNFSEIWIGWQHISNGVGGAqvSAGQRPVFRLVWEKGGLKIMVAYWPYVPYDQSNF
WP_000836126_1_pir  PPQKAPKEHSWVDFAKKYLNMMDYLGTYFLPFYHSFTPIFQWYHPNINPYQRNEFKFQISFRVPVFRHILWTKGTLYLAYTQTNWFQIYNDPQSAPMRMMNFMPELIYVYPINFKPFggNFSEIWIGWQHISNGVGGAqvSAGQRPVFRLVWEKGGLKIMVAYWPYVPYDQSNF
WP_000836139_1_pir  PPQKAPKEHSWVDFAKKYLNMMDYLGTYFLPFYHSFTPIFQWYHPNINPYQRNEFKFQISFRVPVFRHILWTKGTLYLAYTQTNWFQIYNDPQSAPMRMMNFMPELIYVYPINFKPFggNFSEIWIGWQHISNGVGGAqvSAGQRPVFRLVWEKGGLKIMVAYWPYVPYDQSNF
WP_000836141_1_pir  PPQKAPKEHSWVDFAKKYLNMMDYLGTYFLPFYHSFTPIFQWYHPNINPYQRNEFKFQISFRVPVFRHILWTKGTLYLAYTQTNWFQIYNDPQSAPMRMMNFMPELIYVYPINFKPFggNFSEIWIGWQHISNGVGGAqvSAGQRPVFRLVWEKGGLKIMVAYWPYVPYDQSNF
WP_024751357_1_pir  PPQKAPKEHSWVDFAKKYLNMMDYLGTYFLPFYHSFTPIFQWYHPNINPYQRNEFKFQISFRVPVFRHILWTKGTLYLAYTQTNWFQIYNDPQSAPMRMMNFMPELIYVYPINFKPFggNFSEIWIGWQHISNGVGGAqvSAGQRPVFRLVWEKGGLKIMVAYWPYVPYDQSNF
WP_039083150_1_pir  PPQKAPKEHSWVDFAKKYLNMMDYLGTYFLPFYHSFTPIFQWYHPNINPYQRNEFKFQISFRVPVFRHILWTKGTLYLAYTQTNWFQIYNDPQSAPMRMMNFMPELIYVYPINFKPFggNFSEIWIGWQHISNGVGGAqvSAGQRPVFRLVWEKGGLKIMVAYWPYVPYDQSNF
WP_039091217_1_pir  PPQKAPKEHSWVDFAKKYLNMMDYLGTYFLPFYHSFTPIFQWYHPNINPYQRNEFKFQISFRVPVFRHILWTKGTLYLAYTQTNWFQIYNDPQSAPMRMMNFMPELIYVYPINFKPFggNFSEIWIGWQHISNGVGGAqvSAGQRPVFRLVWEKGGLKIMVAYWPYVPYDQSNF
WP_040156663_1_pir  PPQKAPKEHSWVDFAKKYLNMMDYLGTYFLPFYHSFTPIFQWYHPNINPYQRNEFKFQISFRVPVFRHILWTKGTLYLAYTQTNWFQIYNDPQSAPMRMMNFMPELIYVYPINFKPFggNFSEIWIGWQHISNGVGGAqvSAGQRPVFRLVWEKGGLKIMVAYWPYVPYDQSNF
AFR51725_1_pir      PPQKAPKEHSWVDFAKKYLNMMDYLGTYFLPFYHSFTPIFQWYHPNINPYQRNEFKFQISFRVPVFRHILWTKGTLYLAYTQTNWFQIYNDPQSAPMRMMNFMPELIYVYPINFKPFggNFSEIWIGWQHISNGVGGAqvSAGQRPVFRLVWEKGGLKIMVAYWPYVPYDQSNF
WP_053575307_1_pir  PPQKAPKEHSWVDFAKKYLNMMDYLGTYFLPFYHSFTPIFQWYHPNINPYQRNEFKFQISFRVPVFRHILWTKGTLYLAYTQTNWFQIYNDPQSAPMRMMNFMPELIYVYPINFKPFggNFSEIWIGWQHISNGVGGAqvSAGQRPVFRLVWEKGGLKIMVAYWPYVPYDQSNF
WP_064820651_1_pir  PPQKAPKEHSWVDFAKKYLNMMDYLGTYFLPFYHSFTPIFQWYHPNINPYQRNEFKFQISFRVPVFRHILWTKGTLYLAYTQTNWFQIYNDPQSAPMRMMNFMPELIYVYPINFKPFggNFSEIWIGWQHISNGVGGAqvSAGQRPVFRLVWEKGGLKIMVAYWPYVPYDQSNF
WP_000843892_1_pir  PPQKAPKEHSWVDFAKKYLNMMDYLGTYFLPFYHSFTPIFQWYHPNINPYQRNEFKFQISFRVPVFRHILWTKGTLYLAYTQTNWFQIYNDPQSAPMRMINFMPELIYVYPINFKPFggNFSEIWIGWQHISNGVGGAqvSAGQRPVFRLVWEKGDLKIMVAYWPYIPYDQSNF
WP_033760928_1_pir  APQKAPKEHSWVDFAKKYLNMMDYLGTYFLPFYHSFTPIFQWYHPNINPYQRNEFKFQISFRVPVFRHILWTKGTLYLAYTQTNWFQIYNDPQSAPMRMINFMPELIYVYPINFKPFggNFSEIWIGWQHISNGVGGAqvSAGQRPVFRLVWEKGGLKIMVAYWPYVPYDQSNF
WP_050829765_1_pir  PPQKAPKEHSWVDFAKKYLNMMDYLGTYFLPFYHSFTPIFQWYHPNINPYQRNEFKFQISFRVPVFRHILWTKGTLYLAYTQTDWFQIYNNPQSAPMRMINFMPELIYVYPINFKPFggNFSEIWIGWQHISNGVGGAqvSAGQRPVFRLVWEKGGLKIMVAYWPYVPYDQSNF
WP_042966278_1_pir  PPQKAPKEHSWVDFAKKYLNMMDYLGTYFLPFYHSFTPIFQWYHPNINPYQRNEFKFQISFRVPVFRHILWTKGTLYLAYTQTDWFQIYNDPQSAPMRMMNFMPELIYVYPINFKPFggNFSEIWIGWQHISNGVGGAqvSAGQRPVFRLVWEKGGLKIMVAYWPYVPYDQSNF
AFR51731_1_pir      PPQKAPKEHSWVDFAKKYLNMMDYLGTYFLPFYHSFTPIFQWYHPNINPYQRNEFKFQISFRVPVFRHILWTKGTLYLAYTQTDWFQIYNNPQSAPMRMINFMPELIYVYPINFKPFggNFSEIWIGWQHISNGVGGAqvSAGQRPVFRLVWEKGGLKIMVAYWPYVPYDQSNF
AFR51739_1_pir      PPQKAPKEHSWVDFAKKYLNMMDYLGTYFLPFYHSFTPIFQWYHPNINPYQRNEFKFQISFRVPVFRHILWTKGTLYLAYTQTDWFQIYNNPQSAPMRMINFMPELIYVYPINFKPFggNFSEIWIGWQHISNGVGGAqvSAGQRPVFRLVWEKGGLKIMVAYWPYVPYDQSNF
AFR51744_1_pir      PPQKAPKEHSWVDFAKKYLNMMDYLGTYFLPFYHSFTPIFQWYHPNINPYQRNEFKFQISFRVPVFRHILWTKGTLYLAYTQTDWFQIYNNPQSAPMRMINFMPELIYVYPINFKPFggNFSEIWIGWQHISNGVGGAqvSAGQRPVFRLVWEKGGLKIMVAYWPYVPYDQSNF
AFR51762_1_pir      PPQKAPKEHSWVDFAKKYLNMMDYLGTYFLPFYHSFTPIFQWYHPNINPYQRNEFKFQISFRVPVFRHILWTKGTLYLAYTQTDWFQIYNNPQSAPMRMINFMPELIYVYPINFKPFggNFSEIWIGWQHISNGVGGAqvSAGQRPVFRLVWEKGGLKIMVAYWPYVPYDQSNF
AFR51797_1_pir      PPQKAPKEHSWVDFAKKYLNMMDYLGTYFLPFYHSFTPIFQWYHPNINPYQRNEFKFQISFRVPVFRHILWTKGTLYLAYTQTDWFQIYNNPQSAPMRMINFMPELIYVYPINFKPFggNFSEIWIGWQHISNGVGGAqvSAGQRPVFRLVWEKGGLKIMVAYWPYVPYDQSNF
AFR51832_1_pir      PPQKAPKEHSWVDFAKKYLNMMDYLGTYFLPFYHSFTPIFQWYHPNINPYQRNEFKFQISFRVPVFRHILWTKGTLYLAYTQTDWFQIYNNPQSAPMRMINFMPELIYVYPINFKPFggNFSEIWIGWQHISNGVGGAqvSAGQRPVFRLVWEKGGLKIMVAYWPYVPYDQSNF
AFR51862_1_pir      PPQKAPKEHSWVDFAKKYLNMMDYLGTYFLPFYHSFTPIFQWYHPNINPYQRNEFKFQISFRVPVFRHILWTKGTLYLAYTQTDWFQIYNNPQSAPMRMINFMPELIYVYPINFKPFggNFSEIWIGWQHISNGVGGAqvSAGQRPVFRLVWEKGGLKIMVAYWPYVPYDQSNF
AFR51886_1_pir      PPQKAPKEHSWVDFAKKYLNMMDYLGTYFLPFYHSFTPIFQWYHPNINPYQRNEFKFQISFRVPVFRHILWTKGTLYLAYTQTDWFQIYNNPQSAPMRMINFMPELIYVYPINFKPFggNFSEIWIGWQHISNGVGGAqvSAGQRPVFRLVWEKGGLKIMVAYWPYVPYDQSNF
AFR51900_1_pir      PPQKAPKEHSWVDFAKKYLNMMDYLGTYFLPFYHSFTPIFQWYHPNINPYQRNEFKFQISFRVPVFRHILWTKGTLYLAYTQTDWFQIYNNPQSAPMRMINFMPELIYVYPINFKPFggNFSEIWIGWQHISNGVGGAqvSAGQRPVFRLVWEKGGLKIMVAYWPYVPYDQSNF
AFR51903_1_pir      PPQKAPKEHSWVDFAKKYLNMMDYLGTYFLPFYHSFTPIFQWYHPNINPYQRNEFKFQISFRVPVFRHILWTKGTLYLAYTQTDWFQIYNNPQSAPMRMINFMPELIYVYPINFKPFggNFSEIWIGWQHISNGVGGAqvSAGQRPVFRLVWEKGGLKIMVAYWPYVPYDQSNF
WP_000836109_1_pir  PPQKAPKEHSWVDFAKKYLNMMDYLGTYFLPFYHSFTPIFQWYHPNINPYQRNEFKFQISFRVPVFRHILWTKGTLYLAYTQTDWFQIYNNPQSAPMRMINFMPELIYVYPINFKPFggNFSEIWIGWQHISNGVGGAqvSAGQRPVFRLVWEKGGLKIMVAYWPYVPYDQSNF
WP_000836117_1_pir  PPQKAPKEHSWVDFAKKYLNMMDYLGTYFLPFYHSFTPIFQWYHPNINPYQRNEFKFQISFRVPVFRHILWTKGTLYLAYTQTDWFQIYNNPQSAPMRMINFMPELIYVYPINFKPFggNFSEIWIGWQHISNGVGGAqvSAGQRPVFRLVWEKGGLKIMVAYWPYVPYDQSNF
WP_000843893_1_pir  PPQKAPKEHSWVDFAKKYLNMMDYLGTYFLPFYHSFTPIFQWYHPNINPYQRNEFKFQISFRVPVFRHILWTKGTLYLAYTQTDWFQIYNNPQSAPMRMINFMPELIYVYPINFKPFggNFSEIWIGWQHISNGVGGAqvSAGQRPVFRLVWEKGGLKIMVAYWPYVPYDQSNF
WP_024112196_1_pir  PPQKAPKEHSWVDFAKKYLNMMDYLGTYFLPFYHSFTPIFQWYHPNINPYQRNEFKFQISFRVPVFRHILWTKGTLYLAYTQTDWFQIYNNPQSAPMRMINFMPELIYVYPINFKPFggNFSEIWIGWQHISNGVGGAqvSAGQRPVFRLVWEKGGLKIMVAYWPYVPYDQSNF
WP_024118047_1_pir  PPQKAPKEHSWVDFAKKYLNMMDYLGTYFLPFYHSFTPIFQWYHPNINPYQRNEFKFQISFRVPVFRHILWTKGTLYLAYTQTDWFQIYNNPQSAPMRMINFMPELIYVYPINFKPFggNFSEIWIGWQHISNGVGGAqvSAGQRPVFRLVWEKGGLKIMVAYWPYVPYDQSNF
HPeuAn_pir          PPQKAPKEHSWVDFAKKYLNMMDYLGTYFLPFYHSFTPIFQWYHPNINPYQRNEFKFQISFRVPVFRHILWTKGTLYLAYTQTDWFQIYNNPQSAPMRMINFMPELIYVYPINFKPFggNFSEIWIGWQHISNGVGGAqvSAGQRPVFRLVWEKGGLKIMVAYWPYVPYDQSNF
WP_047419295_1_pir  PPQKAPKEHSWVDFAKKYLNMMDYLGTYFLPFYHSFTPIFQWYHPNINPYQRNEFKFQISFRVPVFRHILWTKGTLYLAYTQTDWFQIYNNPQSAPMRMINFMPELIYVYPINFKPFggNFSEIWIGWQHISNGVGGAqvSAGQRPVFRLVWEKGGLKIMVAYWPYVPYDQSNF
WP_053577360_1_pir  PPQKAPKEHSWVDFAKKYLNMMDYLGTYFLPFYHSFTPIFQWYHPNINPYQRNEFKFQISFRVPVFRHILWTKGTLYLAYTQTDWFQIYNNPQSAPMRMINFMPELIYVYPINFKPFggNFSEIWIGWQHISNGVGGAqvSAGQRPVFRLVWEKGGLKIMVAYWPYVPYDQSNF
WP_064432303_1_pir  PPQKAPKEHSWVDFAKKYLNMMDYLGTYFLPFYHSFTPIFQWYHPNINPYQRNEFKFQISFRVPVFRHILWTKGTLYLAYTQTDWFQIYNNPQSAPMRMINFMPELIYVYPINFKPFggNFSEIWIGWQHISNGVGGAqvSAGQRPVFRLVWEKGGLKIMVAYWPYVPYDQSNF
AFR51855_1_pir      PPQKAPKEHSWVDFAKKYLNMMDYLGTYFLPFYHSFTPIFQWYHPNINPYQRNEFKFQISFRVPVFRHILWTKGTLYLAYTQTDWFQIYNNPQSAPMRMINFMPELIYVYPINFKPFggNFSEIWIGWQHISNGVGGAqvSAGQRPVFRLVWEKGGLKIMVAYWPYVPYDQSNF
AFR51729_1_pir      PPQKAPKEHSWVDFAKKYLNMMDYLGTYFLPFYHSFTPIFQWYHPNINPYQRNEFKFQISFRVPVFRHILWTKGTLYLAYTQTNWFQIYNNPQSAPMRMINFMPELIYVYPINFKPFggNFSEIWIGWQHISNGVGGVqvSAGQRPVFRLVWEKGGLKIMVAYWPYVPYDQSNF
AFR51824_1_pir      APQKAPKEHSWVDFAKKYLNMMDYLGTYFLPFYHSFTPIFQWYHPNINPYQRNEFKFQISFRVPVFRHILWTKGTLYLAYTQTNWFQIYNDPQSAPMRMMNFMPELIYVYPINFKPFggNFSEIWIGWQHISNGVGGAqvSAGQRPVFRLVWEKGGLKIMVAYWPYVPYDQSNF
AFR51747_1_pir      PPQKAPKEHSWVDFAKKYLNMMDYLGTYFLPFYHSFTPIFQWYHPNINPYQRNEFKFQISFRVPVFRHILWTKGTLYLAYTQTDWFQIYNDPQSAPMRMMNFMPELIYVYPINFKPFggNFSEIWIGWQHISNGVGGAqvSAGQRPVFRLVWEKGGLKIMVAYWPYVPYDQSNF
AFR51791_1_pir      PPQKAPKEHSWVDFAKKYLNMMDYLGTYFLPFYHSFTPIFQWYHPNINPYQRNEFKFQISFRVPVFRHILWTKGTLYLAYTQTDWFQIYNDPQSAPMRMMNFMPELIYVYPINFKPFggNFSEIWIGWQHISNGVGGAqvSAGQRPVFRLVWEKGGLKIMVAYWPYVPYDQSNF
AFR51803_1_pir      PPQKAPKEHSWVDFAKKYLNMMDYLGTYFLPFYHSFTPIFQWYHPNINPYQRNEFKFQISFRVPVFRHILWTKGTLYLAYTQTDWFQIYNDPQSAPMRMMNFMPELIYVYPINFKPFggNFSEIWIGWQHISNGVGGAqvSAGQRPVFRLVWEKGGLKIMVAYWPYVPYDQSNF
AFR51810_1_pir      PPQKAPKEHSWVDFAKKYLNMMDYLGTYFLPFYHSFTPIFQWYHPNINPYQRNEFKFQISFRVPVFRHILWTKGTLYLAYTQTDWFQIYNDPQSAPMRMMNFMPELIYVYPINFKPFggNFSEIWIGWQHISNGVGGAqvSAGQRPVFRLVWEKGGLKIMVAYWPYVPYDQSNF
AFR51818_1_pir      PPQKAPKEHSWVDFAKKYLNMMDYLGTYFLPFYHSFTPIFQWYHPNINPYQRNEFKFQISFRVPVFRHILWTKGTLYLAYTQTDWFQIYNDPQSAPMRMMNFMPELIYVYPINFKPFggNFSEIWIGWQHISNGVGGAqvSAGQRPVFRLVWEKGGLKIMVAYWPYVPYDQSNF
AFR51822_1_pir      PPQKAPKEHSWVDFAKKYLNMMDYLGTYFLPFYHSFTPIFQWYHPNINPYQRNEFKFQISFRVPVFRHILWTKGTLYLAYTQTDWFQIYNDPQSAPMRMMNFMPELIYVYPINFKPFggNFSEIWIGWQHISNGVGGAqvSAGQRPVFRLVWEKGGLKIMVAYWPYVPYDQSNF
AFR51834_1_pir      PPQKAPKEHSWVDFAKKYLNMMDYLGTYFLPFYHSFTPIFQWYHPNINPYQRNEFKFQISFRVPVFRHILWTKGTLYLAYTQTDWFQIYNDPQSAPMRMMNFMPELIYVYPINFKPFggNFSEIWIGWQHISNGVGGAqvSAGQRPVFRLVWEKGGLKIMVAYWPYVPYDQSNF
AFR51881_1_pir      PPQKAPKEHSWVDFAKKYLNMMDYLGTYFLPFYHSFTPIFQWYHPNINPYQRNEFKFQISFRVPVFRHILWTKGTLYLAYTQTDWFQIYNDPQSAPMRMMNFMPELIYVYPINFKPFggNFSEIWIGWQHISNGVGGAqvSAGQRPVFRLVWEKGGLKIMVAYWPYVPYDQSNF
AFR51911_1_pir      PPQKAPKEHSWVDFAKKYLNMMDYLGTYFLPFYHSFTPIFQWYHPNINPYQRNEFKFQISFRVPVFRHILWTKGTLYLAYTQTDWFQIYNDPQSAPMRMMNFMPELIYVYPINFKPFggNFSEIWIGWQHISNGVGGAqvSAGQRPVFRLVWEKGGLKIMVAYWPYVPYDQSNF
AFR51914_1_pir      PPQKAPKEHSWVDFAKKYLNMMDYLGTYFLPFYHSFTPIFQWYHPNINPYQRNEFKFQISFRVPVFRHILWTKGTLYLAYTQTDWFQIYNDPQSAPMRMMNFMPELIYVYPINFKPFggNFSEIWIGWQHISNGVGGAqvSAGQRPVFRLVWEKGGLKIMVAYWPYVPYDQSNF
AFR51937_1_pir      PPQKAPKEHSWVDFAKKYLNMMDYLGTYFLPFYHSFTPIFQWYHPNINPYQRNEFKFQISFRVPVFRHILWTKGTLYLAYTQTDWFQIYNDPQSAPMRMMNFMPELIYVYPINFKPFggNFSEIWIGWQHISNGVGGAqvSAGQRPVFRLVWEKGGLKIMVAYWPYVPYDQSNF
```

WP_000836138_1_pir_____________PPQKAPKEHSWVDFAKKYLNMMDYLGTYFLPFYHSFTPIFQWYHPNINPYQRNEFKFQISFRVPVFRHILWTKGTLYLAYTQTDWFQIYNDPQSAPMRMMNFMPELIYVYPINFKPFggNFSEIWIGWQHISNGVGGAqvSAGQRPVFRLVWEKGGLKIMVAYWPYVPYDQSN
WP_000836615_1_pir_____________PPQKAPKEHSWVDFAKKYLNMMDYLGTYFLPFYHSFTPIFQWYHPNINPYQRNEFKFQISFRVPVFRHILWTKGTLYLAYTQTDWFQIYNDPQSAPMRMMNFMPELIYVYPINFKPFggNFSEIWIGWQHISNGVGGAqvSAGQRPVFRLVWEKGGLKIMVAYWPYVPYDQSN
WP_015056046_1_pir_____________PPQKAPKEHSWVDFAKKYLNMMDYLGTYFLPFYHSFTPIFQWYHPNINPYQRNEFKFQISFRVPVFRHILWTKGTLYLAYTQTDWFQIYNDPQSAPMRMMNFMPELIYVYPINFKPFggNFSEIWIGWQHISNGVGGAqvSAGQRPVFRLVWEKGGLKIMVAYWPYVPYDQSN
WP_023526083_1_pir_____________PPQKAPKEHSWVDFAKKYLNMMDYLGTYFLPFYHSFTPIFQWYHPNINPYQRNEFKFQISFRVPVFRHILWTKGTLYLAYTQTDWFQIYNDPQSAPMRMMNFMPELIYVYPINFKPFggNFSEIWIGWQHISNGVGGAqvSAGQRPVFRLVWEKGGLKIMVAYWPYVPYDQSN
WP_033617200_1_pir_____________PPQKAPKEHSWVDFAKKYLNMMDYLGTYFLPFYHSFTPIFQWYHPNINPYQRNEFKFQISFRVPVFRHILWTKGTLYLAYTQTDWFQIYNDPQSAPMRMMNFMPELIYVYPINFKPFggNFSEIWIGWQHISNGVGGAqvSAGQRPVFRLVWEKGGLKIMVAYWPYVPYDQSN
AFR51724_1_pir_________________PPQKAPKEHSWVDFAKKYLNMMDYLGTYFLPFYHSFTPIFQWYHPNINPYQRNEFKFQISFRVPVFRHILWTKGTLYLAYTQTNWFQIYNDPQSAPMRMINFMPELIYVYPINFKPFggNFSEIWIGWQHISNGVGGAqvSAGQRPVFRLVWEKGGLKIMVAYWPYVPYDQSN
AFR51737_1_pir_________________PPQKAPKEHSWVDFAKKYLNMMDYLGTYFLPFYHSFTPIFQWYHPNINPYQRNEFKFQISFRVPVFRHILWTKGTLYLAYTQTNWFQIYNDPQSAPMRMINFMPELIYVYPINFKPFggNFSEIWIGWQHISNGVGGAqvSAGQRPVFRLVWEKGGLKIMVAYWPYVPYDQSN
AFR51757_1_pir_________________PPQKAPKEHSWVDFAKKYLNMMDYLGTYFLPFYHSFTPIFQWYHPNINPYQRNEFKFQISFRVPVFRHILWTKGTLYLAYTQTNWFQIYNDPQSAPMRMINFMPELIYVYPINFKPFggNFSEIWIGWQHISNGVGGAqvSAGQRPVFRLVWEKGGLKIMVAYWPYVPYDQSN
WP_000843884_1_pir_____________APQKAPKEHSWVDFAKKYLNMMDYLGTYFLPFYHSFTPIFQWYHPNINPYQRNEFKFQISFRVPVFRHILWTKGTLYLAYTQTNWFQIYNNPQSAPMRMINFMPELIYVYPINFKPFggNFSEIWIGWQHISNGVGGAqvSAGQRPVFRLVWEKGGLKIMVAYWPYVPYDQSN
WP_001938591_1_pir_____________APQKAPKEHSWVDFAKKYLNMMDYLGTYFLPFYHSFTPIFQWYHPNINPYQRNEFKFQISFRVPVFRHILWTKGTLYLAYTQTNWFQIYNNPQSAPMRMINFMPELIYVYPINFKPFggNFSEIWIGWQHISNGVGGAqvSAGQRPVFRLVWEKGGLKIMVAYWPYVPYDQSN
WP_000836091_1_pir_____________PPQKAPKEHSWVDFAKKYLNMMDYLGTYFLPFYHSFTPIFQWYHPNINPYQRNEFKFQISFRVPVFRHILWTKGTLYLAYTQTNWFQIYNDPQSAPMRMINFMPELIYVYPINFKPFggNFSEIWIGWQHISNGVGGAqvSAGQRPVFRLVWEKGGLKIMVAYWPYVPYDQSN
WP_001925679_1_pir_____________PPQKAPKEHSWVDFAKKYLNMMDYLGTYFLPFYHSFTPIFQWYHPNINPYQRNEFKFQISFRVPVFRHILWTKGTLYLAYTQTNWFQIYNDPQSAPMRMINFMPELIYVYPINFKPFggNFSEIWIGWQHISNGVGGTqvSAGQRPVFRLVWEKGGLKIMVAYWPYVPYDQSN
WP_000836147_1_pir_____________PPQKAPKEHSWVDFAKKYLNMMDYLGTYFLPFYHSFTPIFQWYHPNINPYQRNEFKFQISFRVPVFRHILWTKGTLYLAYTQTDWFQIYNDPQSAPMRMINFMPELIYVYPINFKPFggNFSEIWIGWQHISNGVGGAqvSAGQRPVFRLVWEKGGLKIMVAYWPYVPYDQSN
WP_020849831_1_pir_____________PPQKAPKEHSWVDFAKKYLNMMDYLGTYFLPFYHSFTPIFQWYHPNINPYQRNEFKFQISFRVPVFRHILWTKGTLYLAYTQTDWFQIYNNPQSAPMRMINFMPELIYVYPINFKPFggNFSEIWIGWQHISNGVGGAqvSAGQRPVFRLVWEKGGLKIMVAYWPYVPYDQSN
WP_020849831_1_pir_____________PPQKAPKEHSWVDFAKKYLNMMDYLGTYFLPFYHSFTPIFQWYHPNINPYQRNEFKFQISFRVPVFRHILWTKGTLYLAYTQTDWFQIYNNPQSAPMRMINFMPELIYVYPINFKPFggNFSEIWIGWQHISNGVGGAqvSAGQRPVFRLVWEKGGLKIMVAYWPYVPYDQSN
WP_001949636_1_pir_____________PPQKAPKEYSWVDFAKKYLNMMDYLGTYFLPFYHSFTPIFQWYHPNINPYQRNEFKFQISFRVPVFRHILWTKGTLYLAYTQTNWFQIYNDPQSAPMRMINFMPELIYVYPINFKPFggNFSEIWIGWQHISNGVGGAqvSAGQRPVFRLVWEKGGLKIMVAYWPYIPYDQSN
AFR51904_1_pir_________________PPQKAPKEHSWVDFAKKYLNMMDYLGTYFLPFYHSFTPIFQWYHPNINPYQRNEFKFQISFRVPVFRNILWTKGTLYLAYTQTDWFQIYNNPQSAPMRMINFMPELIYVYPINFKPFggNFSEIWIGWQHISNGVGGAqvSAGQRPVFRLVWEKGGLKIMVAYWPYVPYDQSN
AFR51804_1_pir_________________PPQKAPKEHSWVDFAKKYLNMMDYLGTYFLPFYHSFTPIFQWYHPNINPYQRNEFKFQISFRVPVFRHILWTKGTLYLAYTQTNWFQIYNDPQSAPMRMMNFMPELIYVYPINFKPFggNFSEIWIGWQHISNGVGGAqvSAVQRPVFRLVWEKGGLKIMVAYWPYVPYDQSN
WP_001962022_1_pir_____________APQKAPKEHSWVDFAKKYLNMMDYLGTYFLPFYHSFTPIFQWYHPNINPYQRNEFKFQISFRVPVFRHILWTKGTLYLAYTQTNWFQIYNDPQSAPMRMINFMPELIYVYPINFKPFggNFSEIWIGWQHISNGVGGAqvSAGQRPVFRLVWEKGGLKIMVAYWPYVPYDQSN
AFR51720_1_pir_________________PPQKAPKEHSWVDFAKKYLNMMDYLGTYFLPFYHSFTPIFQWYHPNINPYQRNEFKFQISFRVPVFRHILWTKGTLYLAYTQTDWFQIYNDPQSAPMRMINFMPELIYVYPINFKPFggNFSEIWIGWQHISNGVGGAqvSAGQRPVFRLVWEKGGLKIMVAYWPYVPYDQSN
AFR51745_1_pir_________________PPQKAPKEHSWVDFAKKYLNMMDYLGTYFLPFYHSFTPIFQWYHPNINPYQRNEFKFQISFRVPVFRHILWTKGTLYLAYTQTDWFQIYNDPQSAPMRMINFMPELIYVYPINFKPFggNFSEIWIGWQHISNGVGGAqvSAGQRPVFRLVWEKGGLKIMVAYWPYVPYDQSN
AFR51831_1_pir_________________PPQKAPKEHSWVDFAKKYLNMMDYLGTYFLPFYHSFTPIFQWYHPNINPYQRNEFKFQISFRVPVFRHILWTKGTLYLAYTQTDWFQIYNDPQSAPMRMINFMPELIYVYPINFKPFggNFSEIWIGWQHISNGVGGAqvSAGQRPVFRLVWEKGGLKIMVAYWPYVPYDQSN
WP_021187454_1_pir_____________PPQKAPKEHSWVDFAKKYLNMMDYLGTYFLPFYHSFTPIFQWYHPNINPYQRNEFKFQISFRVPVFRHILWTKGTLYLAYTQTNWFQIYNDPQSAPMRMINFMPELIYVYPINFKPFggNFSEIWIGWQHISNGVGGVqvSAGQRPVFRLVWEKGGLKIMVAYWPYVPYDQSN
AFR51917_1_pir_________________PPQKAPKEHSWVDFAKKYLNMMDYLGTYFLPFYHSFTPIFQWYHPNINPYQRNEFKFQISFRVPVFRHILWTKGTLYLAYTQTDWFQIYNNPQSAPMRMINFMPELIYVYPINFKPFggNFSEIWIGWQHISNGVGGAqvSAGQRPVFRLVWEKGDLKIMVAYWPYVPYDQSN
AFR51776_1_pir_________________PPQKAPKEHSWVDFAKKYLNMMDYLGTYFLPFYHSFTPIFQWYHPNINPYQRNEFKFQISFRVPVFRHILWTKGTLYLAYTQTNWFQIYNDPQSAPMRMINFMPELIYVYPINFEPFggKFSEIWIGWQHISNGVGGAqvSAGQRPVFRLVWEKGGLKIMVAYWPYVPYDQSN
WP_000836120_1_pir_____________PPQKAPKEHSWVDFAKKYLNMMDYLGTYFLPFYHSFTPIFQWYHPNINPYQRNEFKFQISFRVPVFRHILWTKGTLYLAYTQTNWFQIYNDPQSAPMRMINFMPELIYVYPINFEPFggKFSEIWIGWQHISNGVGGAqvSAGQRPVFRLVWEKGGLKIMVAYWPYVPYDQSN
WP_000836142_1_pir_____________PPQKAPKEHSWVDFAKKYLNMMDYLGTYFLPFYHSFTPIFQWYHPNINPYQRNEFKFQISFRVPVFRHILWTKGTLYLAYTQTNWFQIYNDPQSAPMRMINFMPELIYVYPINFEPFggKFSEIWIGWQHISNGVGGAqvSAGQRPVFRLVWEKGGLKIMVAYWPYVPYDQSN
WP_015427651_1_pir_____________PPQKAPKEHSWVDFAKKYLNMMDYLGTYFLPFYHSFTPIFQWYHPNINPYQRNEFKFQISFRVPVFRHILWTKGTLYLAYTQTNWFQIYNDPQSAPMRMINFMPELIYVYPINFEPFggKFSEIWIGWQHISNGVGGAqvSAGQRPVFRLVWEKGGLKIMVAYWPYVPYDQSN
WP_025288024_1_pir_____________PPQKAPKEHSWVDFAKKYLNMMDYLGTYFLPFYHSFTPIFQWYHPNINPYQRNEFKFQISFRVPVFRHILWTKGTLYLAYTQTNWFQIYNDPQSAPMRMINFMPELIYVYPINFEPFggKFSEIWIGWQHISNGVGGAqvSAGQRPVFRLVWEKGGLKIMVAYWPYVPYDQSN
WP_025366794_1_pir_____________PPQKAPKEHSWVDFAKKYLNMMDYLGTYFLPFYHSFTPIFQWYHPNINPYQRNEFKFQISFRVPVFRHILWTKGTLYLAYTQTNWFQIYNDPQSAPMRMINFMPELIYVYPINFEPFggKFSEIWIGWQHISNGVGGAqvSAGQRPVFRLVWEKGGLKIMVAYWPYVPYDQSN
AFR51942_1_pir_________________PPQKAPKEHSWVDFAKKYLNMMDYLGTYFLPFYHSFTPIFQWYHPNINPYQRNEFKFQISFRVPVFRHILWTKGTLYLAYTQTNWFQIYNDPQSAPMRMMNFMPELIYVYPINFKPFggNFSEIWIGWQHISNGVGGAqvSAGQRPVFRLVWEKGGLKIMVAYWPYVPYDQSN
WP_000836108_1_pir_____________PPQKAPKEHSWVDFAKKYLNMMDYLGTYFLPFYHSFTPIFQWYHPNINPYQRNEFKFQISFRVPVFRHILWTKGTLYLAYTQTDWFQIYNDPQSAPMRMMNFMPELIYVYPINFKPFggNFSEIWIGWQHISNGVGGAqvSAGQRPVFRLVWEKGDLKIMVAYWPYVPYDQSN
AFR51738_1_pir_________________PPQKAPKEHSWVDFAKKYLNTMDYLGTYFLPFYHSFTPIFQWYHPNINPYQRNEFKFQISFRVPVFRHILWTKGTLYLAYTQTDWFQIYNDPQSAPMRMINFMPELIYVYPINFKPFggNFSEIWIGWQHISNGVGGAqvSAGQRPVFRLVWEKGGLKIMVAYWPYVPYDQSN
AFR51884_1_pir_________________PPQKAPKEHSWVDFAKKYLNMMDYLGTYFLPFYHSFTPIFQWYHPNINPYQRNEFKFQISFRVPVFRHILWTKGTLYLAYTQTDWFQIYNNPQSAPMRMINFMPELIYVYPINFKPFggNFSEIWIGWQHISNGVGGAqvNAGQRPVFRLVWEKGGLKIMVAYWPYVPYDQSN
AFR51735_1_pir_________________PPQKAPKEHSWVDFAKKYLNMMDYLGTYFLPFYHSFTPIFQWYHPNINPYQRNEFKFQISFRVPVFRHILWTKGTLYLAYTQTDWFQIYNNPQSAPMRMMNFMPELIYVYPINFKPFggNFSEIWIGWQHISNGVGGAqvSAGQRPVFRLVWEKGGLKIMVAYWPYVPYDQSN
AFR51820_1_pir_________________PPQKAPKEHSWVDFAKKYLNMMDYLGTYFLPFYHSFTPIFQWYHPNINPYQRNEFKFQISFRVPVFRHILWTKGTLYLAYTQTDWFQIYNNPQSAPMRMMNFMPELIYVYPINFKPFggNFSEIWIGWQHISNGVGGAqvSAGQRPVFRLVWEKGGLKIMVAYWPYVPYDQSN
AFR51877_1_pir_________________PPQKAPKEHSWVDFAKKYLNMMDYLGTYFLPFYHSFTPIFQWYHPNINPYQRNEFKFQISFRVPVFRHILWTKGTLYLAYTQTDWFQIYNNPQSAPMRMMNFMPELIYVYPINFKPFggNFSEIWIGWQHISNGVGGAqvSAGQRPVFRLVWEKGGLKIMVAYWPYVPYDQSN
AFR51908_1_pir_________________PPQKAPKEHSWVDFAKKYLNMMDYLGTYFLPFYHSFTPIFQWYHPNINPYQRNEFKFQISFRVPVFRHILWTKGTLYLAYTQTDWFQIYNNPQSAPMRMMNFMPELIYVYPINFKPFggNFSEIWIGWQHISNGVGGAqvSAGQRPVFRLVWEKGGLKIMVAYWPYVPYDQSN
WP_033613327_1_pir_____________APQKAPKEHSWVDFAKKYLDMMDYLGTYFLPFYHSFTPIFQWYHPNINPYQRNEFKFQISFRVPVFRHILWTKGTLYLAYTQTNWFQIYNDPQSAPMRMINFMPELIYVYPINFKPFggNFSEIWIGWQHISNGVGGAqvSAGQRPVFRLVWEKGGLKIMVAYWPYIPYDQSN
AFR51795_1_pir_________________PPQKAPKEHSWVDFAKKYLNMMDYLGTYFLPFYHSFTPIFQWYHPNINPYQRNEFKFQISFRVPVFRHILWTKGTLYLAYTQTDWFQIYNDPQSAPMRMMNFMPELIYVYPINFKPFggNFSEIWIGWQHISNGVGGAqvNAGQRPVFRLVWEKGGLKIMVAYWPYVPYDQSN
AFR51741_1_pir_________________PPQKAPKEHSWVDFAKKYLNMMDYLGTYFLPFYHSFTPIFQWYHPNINPYQRNEFKFQISFRVPVFRHILWTKGTLYLAYTQTNWFQIYNDPQSAPMRMMNFMPELIYVYPINFKPFggNFSEIWIGWQHISNGVGGAqvSAGQRPVFRLVWEKGGLKIMVAYWPYVPYDQSN
AFR51750_1_pir_________________PPQKAPKEHSWVDFAKKYLNMMDYLGTYFLPFYHSFTPIFQWYHPNINPYQRNEFKFQISFRVPVFRHILWTKGTLYLAYTQTNWFQIYNDPQSAPMRMMNFMPELIYVYPINFKPFggNFSEIWIGWQHISNGVGGAqvSAGQRPVFRLVWEKGGLKIMVAYWPYVPYDQSN
AFR51764_1_pir_________________PPQKAPKEHSWVDFAKKYLNMMDYLGTYFLPFYHSFTPIFQWYHPNINPYQRNEFKFQISFRVPVFRHILWTKGTLYLAYTQTNWFQIYNDPQSAPMRMMNFMPELIYVYPINFKPFggNFSEIWIGWQHISNGVGGAqvSAGQRPVFRLVWEKGGLKIMVAYWPYVPYDQSN
AFR51766_1_pir_________________PPQKAPKEHSWVDFAKKYLNMMDYLGTYFLPFYHSFTPIFQWYHPNINPYQRNEFKFQISFRVPVFRHILWTKGTLYLAYTQTNWFQIYNDPQSAPMRMMNFMPELIYVYPINFKPFggNFSEIWIGWQHISNGVGGAqvSAGQRPVFRLVWEKGGLKIMVAYWPYVPYDQSN
AFR51875_1_pir_________________PPQKAPKEHSWVDFAKKYLNMMDYLGTYFLPFYHSFTPIFQWYHPNINPYQRNEFKFQISFRVPVFRHILWTKGTLYLAYTQTNWFQIYNDPQSAPMRMMNFMPELIYVYPINFKPFggNFSEIWIGWQHISNGVGGAqvSAGQRPVFRLVWEKGGLKIMVAYWPYVPYDQSN
AFR51885_1_pir_________________PPQKAPKEHSWVDFAKKYLNMMDYLGTYFLPFYHSFTPIFQWYHPNINPYQRNEFKFQISFRVPVFRHILWTKGTLYLAYTQTNWFQIYNDPQSAPMRMMNFMPELIYVYPINFKPFggNFSEIWIGWQHISNGVGGAqvSAGQRPVFRLVWEKGGLKIMVAYWPYVPYDQSN
AFR51929_1_pir_________________PPQKAPKEHSWVDFAKKYLNMMDYLGTYFLPFYHSFTPIFQWYHPNINPYQRNEFKFQISFRVPVFRHILWTKGTLYLAYTQTNWFQIYNDPQSAPMRMMNFMPELIYVYPINFKPFggNFSEIWIGWQHISNGVGGAqvSAGQRPVFRLVWEKGGLKIMVAYWPYVPYDQSN
AFR51936_1_pir_________________PPQKAPKEHSWVDFAKKYLNMMDYLGTYFLPFYHSFTPIFQWYHPNINPYQRNEFKFQISFRVPVFRHILWTKGTLYLAYTQTNWFQIYNDPQSAPMRMMNFMPELIYVYPINFKPFggNFSEIWIGWQHISNGVGGAqvSAGQRPVFRLVWEKGGLKIMVAYWPYVPYDQSN
WP_000836123_1_pir_____________PPQKAPKEHSWVDFAKKYLNMMDYLGTYFLPFYHSFTPIFQWYHPNINPYQRNEFKFQISFRVPVFRHILWTKGTLYLAYTQTNWFQIYNDPQSAPMRMINFMPELIYVYPINFKPFggNFSEIWIGWQHISNGVGGAqvSAGQRPVFRLVWEKGGLKIMVAYWPYVPYNQSN
AFR51780_1_pir_________________PPQKAPKEHSWVDFAKKYLNMMDYLGTYFLPFYHSFTPIFQWYHPNINPYQRNEFKFQISFRVPVFRHILWTKGTLYLAYTQTNWFQIYNNPQSAPMRMINFMPELIYVYPINFEPFggKFSEIWIGWQHISNGVGGAqvSAGQRPVFRLVWEKGGLKIMVAYWPYVPYDQSN
AFR51860_1_pir_________________PPQKAPKEHSWVDFAKKYLNMMDYLGTYFLPFYHSFTPIFQWYHPNINPYQRNEFKFQISFRVPVFRHIFWTKGTLYLAYTQTNWFQIYNDPQSAPMRMMNFMPELIYVYPINFKPFggNFSEIWIGWQHISNGVGGAqvSAGQRPVFRLVWEKGGLKIMVAYWPYVPYDQSN
AFR51783_1_pir_________________PPQKAPKEHSWVDFAKKYLNMMDYLGTYFLPFYHSFTPIFQWYHPNINPYQRNEFKFQISFRVPVFRHILWTKGTLYLAYTQTDWFQIYNDPQSAPMRMINFMPELIYVYPINFEPFggKFSEIWIGWQHISNGVGGAqvSAGQRPVFRLVWEKGGLKIMVAYWPYVPYDQSN
WP_000836104_1_pir_____________PPQKAPKEYSWVDFAKKYLNMMDYLGTYFLPFYHSFTPIFQWYHPNINPYQRNEFKFQISFRVPVFRHILWTKGTLYLAYTQTNWFQIYNNPQSAPMRMINFMPELIYVYPINFKPFggNFSEIWIGWQHISNGVGGAqvSAGQRPVFRLVWEKGGLKIMVAYWPYIPYDQSN
AFR51821_1_pir_________________PPQKAPKEHSWVDFAKKYLNMMDYLGTYFLPFYHSFTPIFQWYHPNINPYQRNEFKFQISFRVPVFRHILWTKGTLYLAYTQTDWFQIYNNPQSAPMRMINFMPELIYVYPINFKPFggNFSEIWIGWQHISNGVGGAqvSAGQRPVFRLVWEKGGLKIMVAYWPYVPYDQSN
AFR51719_1_pir_________________PPQKAPKEHSWVDFAKKYLNMMDYLGTYFLPFYHSFTPIFQWYHPNINPYQRNEFKFQISFRVPVFRHILWTKGTLYLAYTQTDWFQIYNDPQSAPMRMMNFMPELIYVYPINFKPFggNFSEIWIGWQHISNGVGGAqvSAGQRPVFRLVWEKGGLKIMVAYWPYVPYDQSN
AFR51856_1_pir_________________PPQKAPKEHSWVDFAKKYLNMMDYLGTYFLPFYHSFTPIFQWYHPNINPYQRNEFKFQISFRVPVFRHILWTKGTLYLAYTQTDWFQIYNDPQSAPMRMMNFMPELIYVYPINFKPFggNFSEIWIGWQHISNGVGGAqvSAGQRPVFRLVWEKGGLKIMVAYWPYVPYDQSN
AFR51736_1_pir_________________PPQKAPKEHSWVDFAKKYLNMMDYLGTYFLPFYHSFTPIFQWYHPNINPYQRNEFKFQISFRVPVFRHILWTKGTLYLAYTQTNWFQIYNDPQSAPMRMMNFMPELIYVYPINFKPFggNFSEIWIGWQHISNGVGGVqvSAGQRPVFRLVWEKGGLKIMVAYWPYVPYDQSN
EJB14110_1_pir_________________PPQKAPKEHSWVDFAKKYLNMMDYLGTYFLPFYHSFTPIFQWYHPNINPYQRNEFKFQISFRVPVFRHILWTKGTLYLAYTQTNWFQIYNNPQSAPMRMINFMPELIYVYPINFEPFggKFSEIWIGWQHISNGVGGAqvSAGQRPVFRLVWEKGGLKIMVAYWPYVPYDQSN
EJB14110_1_pir_________________PPQKAPKEHSWVDFAKKYLNMMDYLGTYFLPFYHSFTPIFQWYHPNINPYQRNEFKFQISFRVPVFRHILWTKGTLYLAYTQTNWFQIYNNPQSAPMRMINFMPELIYVYPINFEPFggKFSEIWIGWQHISNGVGGAqvSAGQRPVFRLVWEKGGLKIMVAYWPYVPYDQSN
AFR51782_1_pir_________________PPQKAPKEHSWVDFAKKYLNMMDYLGTYFLPFYHSFTPIFQWYHPNINPYQRNEFKFQISFRVPVFRHILWTKGTLYLAYTQTNWFQIYNNPQSAPMRMINFMPELIYVYPINFEPFggKFSEIWIGWQHISNGVGGAqvSAGQRPVFRLVWEKGGLKIMVAYWPYVPYDQSN
WP_000836101_1_pir_____________PPQKAPKEHSWVDFAKKYLNMMDYLGTYFLPFYHSFTPIFQWYHPNINPYQRNEFKFQISFRVPVFRHILWTKGTLYLAYTQTNWFQIYNNPQSAPMRMINFMPELIYVYPINFEPFggKFSEIWIGWQHISNGVGGAqvSAGQRPVFRLVWEKGGLKIMVAYWPYVPYDQSN
WP_000836111_1_pir_____________PPQKAPKEHSWVDFAKKYLNMMDYLGTYFLPFYHSFTPIFQWYHPNINPYQRNEFKFQISFRVPVFRHILWTKGTLYLAYTQTNWFQIYNNPQSAPMRMINFMPELIYVYPINFEPFggKFSEIWIGWQHISNGVGGAqvSAGQRPVFRLVWEKGGLKIMVAYWPYVPYDQSN
WP_000836129_1_pir_____________PPQKAPKEHSWVDFAKKYLNMMDYLGTYFLPFYHSFTPIFQWYHPNINPYQRNEFKFQISFRVPVFRHILWTKGTLYLAYTQTNWFQIYNNPQSAPMRMINFMPELIYVYPINFEPFggKFSEIWIGWQHISNGVGGAqvSAGQRPVFRLVWEKGGLKIMVAYWPYVPYDQSN
WP_000836130_1_pir_____________PPQKAPKEHSWVDFAKKYLNMMDYLGTYFLPFYHSFTPIFQWYHPNINPYQRNEFKFQISFRVPVFRHILWTKGTLYLAYTQTNWFQIYNNPQSAPMRMINFMPELIYVYPINFEPFggKFSEIWIGWQHISNGVGGAqvSAGQRPVFRLVWEKGGLKIMVAYWPYVPYDQSN
WP_000836132_1_pir_____________PPQKAPKEHSWVDFAKKYLNMMDYLGTYFLPFYHSFTPIFQWYHPNINPYQRNEFKFQISFRVPVFRHILWTKGTLYLAYTQTNWFQIYNNPQSAPMRMINFMPELIYVYPINFEPFggKFSEIWIGWQHISNGVGGAqvSAGQRPVFRLVWEKGGLKIMVAYWPYVPYDQSN
WP_000836143_1_pir_____________PPQKAPKEHSWVDFAKKYLNMMDYLGTYFLPFYHSFTPIFQWYHPNINPYQRNEFKFQISFRVPVFRHILWTKGTLYLAYTQTNWFQIYNNPQSAPMRMINFMPELIYVYPINFEPFggKFSEIWIGWQHISNGVGGAqvSAGQRPVFRLVWEKGGLKIMVAYWPYVPYDQSN
WP_000836613_1_pir_____________PPQKAPKEHSWVDFAKKYLNMMDYLGTYFLPFYHSFTPIFQWYHPNINPYQRNEFKFQISFRVPVFRHILWTKGTLYLAYTQTNWFQIYNNPQSAPMRMINFMPELIYVYPINFEPFggKFSEIWIGWQHISNGVGGAqvSAGQRPVFRLVWEKGGLKIMVAYWPYVPYDQSN
WP_024116023_1_pir_____________PPQKAPKEHSWVDFAKKYLNMMDYLGTYFLPFYHSFTPIFQWYHPNINPYQRNEFKFQISFRVPVFRHILWTKGTLYLAYTQTNWFQIYNNPQSAPMRMINFMPELIYVYPINFEPFggKFSEIWIGWQHISNGVGGAqvSAGQRPVFRLVWEKGGLKIMVAYWPYVPYDQSN
WP_033582870_1_pir_____________PPQKAPKEHSWVDFAKKYLNMMDYLGTYFLPFYHSFTPIFQWYHPNINPYQRNEFKFQISFRVPVFRHILWTKGTLYLAYTQTNWFQIYNNPQSAPMRMINFMPELIYVYPINFEPFggKFSEIWIGWQHISNGVGGAqvSAGQRPVFRLVWEKGGLKIMVAYWPYVPYDQSN
WP_033586287_1_pir_____________PPQKAPKEHSWVDFAKKYLNMMDYLGTYFLPFYHSFTPIFQWYHPNINPYQRNEFKFQISFRVPVFRHILWTKGTLYLAYTQTNWFQIYNNPQSAPMRMINFMPELIYVYPINFEPFggKFSEIWIGWQHISNGVGGAqvSAGQRPVFRLVWEKGGLKIMVAYWPYVPYDQSN
WP_050852253_1_pir_____________PPQKAPKEHSWVDFAKKYLNMMDYLGTYFLPFYHSFTPIFQWYHPNINPYQRNEFKFQISFRVPVFRHILWTKGTLYLAYTQTNWFQIYNNPQSAPMRMINFMPELIYVYPINFEPFggKFSEIWIGWQHISNGVGGAqvSAGQRPVFRLVWEKGGLKIMVAYWPYVPYDQSN
WP_057112610_1_pir_____________PPQKAPKEHSWVDFAKKYLNMMDYLGTYFLPFYHSFTPIFQWYHPNINPYQRNEFKFQISFRVPVFRHILWTKGTLYLAYTQTNWFQIYNNPQSAPMRMINFMPELIYVYPINFEPFggKFSEIWIGWQHISNGVGGAqvSAGQRPVFRLVWEKGGLKIMVAYWPYVPYDQSN
WP_058914842_1_pir_____________PPQKAPKEHSWVDFAKKYLNMMDYLGTYFLPFYHSFTPIFQWYHPNINPYQRNEFKFQISFRVPVFRHILWTKGTLYLAYTQTNWFQIYNNPQSAPMRMINFMPELIYVYPINFEPFggKFSEIWIGWQHISNGVGGAqvSAGQRPVFRLVWEKGGLKIMVAYWPYVPYDQSN

```
OLR50470_1_pir___________________PPQKAPKEHSWVDFAKKYLNMMDYLGTYFLPFYHSFTPIFQWYHPNINPYQRNEFKFQISFRVPVFRHILWTKGTLYLAYTQTNWFQIYNNPQSAPMRMINFMPELIYVYPINFEPFggKFSEIWIGWQHISNGVGGAqvSAGQRPVFRLVWEKGGLKIMVAYWPYVPYDQSNF
WP_042967153_1_pir_______________PPQKAPKEHSWVDFAKKYLNMMDYLGTYFLPFYHSFTPIFQWYHPNINPYQRNEFKFQISFRVPVFRHILWTKGTLYLAYTQTNWFQIYNNPQSAPMRMINFMPELIYVYPINFKPFggNFSEIWIGWQHISNGVGGAqvSAGQRPVFRLVWEKGGLKIMVAYWPYIPYHQSNF
WP_041051029_1_pir_______________PPQKAPKEHSWVDFAKKYLNMMDYLGTYFLPFYHSFTPIFQWYHPNINPYQRNEFKFQISFRVPVFRHILWTKGTLYLAYTQTNWFQIYNDPQSAPMRMMNFMPELIYVYPINFEPFggKFSEIWIGWQHISNGVGGAqvSAGQRPVFRLVWEKGGLKIMVAYWPYVPYDQSNF
WP_001942292_1_pir_______________APKKAPKEHSWVDFAKKYLNMMDYLGTYFLPFYHSFTPIFQWYHPNINPYQRNEFKFQISFRVPVFRHILWTKGTLYLAYTQTNWFQIYNDPQSAPMRMINFMPELIYVYPINFKPFggNFSEIWIGWQHISNGVGGAqvSAGQRPVFRLVWEKGGLKIMVAYWPYVPYDQSNF
WP_001968310_1_pir_______________APKKAPKEHSWVDFAKKYLNMMDYLGTYFLPFYHSFTPIFQWYHPNINPYQRNEFKFQISFRVPVFRHILWTKGTLYLAYTQTNWFQIYNDPQSAPMRMINFMPELIYVYPINFKPFggNFSEIWIGWQHISNGVGGAqvSAGQRPVFRLVWEKGGLKIMVAYWPYVPYDQSNF
WP_001976950_1_pir_______________APKKAPKEHSWVDFAKKYLNMMDYLGTYFLPFYHSFTPIFQWYHPNINPYQRNEFKFQISFRVPVFRHILWTKGTLYLAYTQTNWFQIYNDPQSAPMRMINFMPELIYVYPINFKPFggNFSEIWIGWQHISNGVGGAqvSAGQRPVFRLVWEKGGLKIMVAYWPYVPYDQSNF
AFR51941_1_pir___________________PPQKAPKEHSWVDFAKKYLNMMDYLGTYFLPFYHSFTPIFQWYHPNINPYQRNEFKFQISFRVPVFRHILWTKGTLYLAYTQTDWFQIYNNPQSAPMRMMNFMPELIYVYPINFKPFggNFSEIWIGWQHISNGVGGAqvNAGQRPVFRLVWEKGGLKIMVAYWPYVPYDQSNF
WP_015644074_1_pir_______________PPQKAPKEHSWVDFAKKYLNMMDYLGTYFLPFYHSFTPIFQWYHPNINPYQRNEFKFQISFRVPVFRHILWTKGTLYLAYTQTNWFQIYNDPQSAPMRMINFMPELIYVYPINFKPFggNFSEIWIGWQHISNGVGGAqvSAGQHPVFRLVWEKGGLKIMVAYWPYVPYDQSNF
AFR51926_1_pir___________________PPQKASKEHSWVDFAKKYLNMMDYLGTYFLPFYHSFTPIFQWYHPNINPYQRNEFKFQISFRVPVFRHILWTKGTLYLAYTQTNWFQIYNDPQSAPMRMINFMPELIYVYPINFKPFggNFSEIWIGWQHISNGVGGAqvSAGQRPVFRLVWEKGGLKIMVAYWPYVPYDQSNF
WP_050852562_1_pir_______________PPQKAPKEHSWVDFAKKYLNMMDYLGTYFLPFYHSFTPIFQWYHPNINSYQRNEFKFQISFRVPVFRHILWTKGTLYLAYTQTDWFQIYNNPQSAPMRMINFMPELIYVYPINFKPFggNFSEIWIGWQHISNGVGGAqvSAGQRPVFRLVWEKGGLKIMVAYWPYVPYDQSNF
WP_000836145_1_pir_______________PPQKAPKEHSWVDFAKKYLNMMDYLGTYFLPFYHSFTPIFQWYHPNINPYQRNEFKFQISFRVPVFRHILWTKGTLYLAYTQTNWFQIYNDPQSAPMRMINFMPELIYVYPINFEPFggKFSEIWIGWQHISNGVGGAqvSAGQRPVFRLVWEKGGLKIMVAYWPYVPYDQSNF
WP_021303427_1_pir_______________PPQKAPKEHSWVDFAKKYLNLMDYLGTYFLPFYHSFTPIFQWYHPNINPYQRNEFKFQISFRVPVFRHILWTKGTLYLAYTQTDWFQIYNNPQSAPMRMINFMPELIYVYPINFKPFggNFSEIWIGWQHISNGVGGAqvSAGQRPVFRLVWEKGGLKIMVAYWPYVPYDQSNF
AFR51785_1_pir___________________PPQKAPKEHSWVDFAKKYLNMMDYLGTYFLPFYHSFTPIFQWYNPNINPYQRNEFKFQISFRVPVFRHILWTKGTLYLAYTQTNWFQIYNDPQSAPMRMINFMPELIYVYPINFKPFggNFSEIWIGWQHISNGVGGAqvSAGQRPVFRLVWEKGGLKIMVAYWPYVPYDQSNF
WP_000836614_1_pir_______________PPQKAPKEHSWVDFAKKYLNMMDYLGTYFLPFYHSFTPIFQWYHPNINPYQRNEFKFQISFRVPVFRHILWTKGTLYLAYTQTDWFQIYNDPQSAPMRMINFMPELIYVYPINFKPFggNFSEIWIGWQHISNGVGGVqvSAGQRPVFRLVWEKGGLKIMVAYWPYVPYDQSNF
WP_033614903_1_pir_______________APQKAAKEHSWVDFAKKYLNMMDYLGTYFLPFYHSFTPIFQWYHPNINPYQRNEFKFQISFRVPVFRHILWTKGTLYLAYTQTNWFQIYNDPQSAPMRMINFMPELIYVYPINFKPFggNFSEIWIGWQHISNGVGGAqvSAGQRPVFRLVWEKGGLKIMVAYWPYVPYDQSNF
WP_033614903_1_pir_______________APQKAAKEHSWVDFAKKYLNMMDYLGTYFLPFYHSFTPIFQWYHPNINPYQRNEFKFQISFRVPVFRHILWTKGTLYLAYTQTNWFQIYNDPQSAPMRMINFMPELIYVYPINFKPFggNFSEIWIGWQHISNGVGGAqvSAGQRPVFRLVWEKGGLKIMVAYWPYVPYDQSNF
AFR51761_1_pir___________________PPQKAPKEHSWVDFAKKYLNMMDYLGTYFLPFYHSFTPIFQWYHPNINPYQRNEFKFQISFRVPVFRHILWTKGTLYLAYTQTDWFQIYNNPQSAPMRMMNFMPELIYVYPINFKPFggNFSEIWIGWQHISNGVGGAqvSAGQRPVFRLVWEKGGLKIMVAYWPYVPYDQSNF
WP_029568087_1_pir_______________PPQKAPKEHSWVDFAKKYLNMMDYLGTYFLPFYHSFTPIFQWYHPNINPYQRNEFKFQISFRVPVFRHILWTKGTLYLAYTQTNWFQIYNNPQSAPMRMMNFMPELIYVYPINFEPFggKFSEIWIGWQHISNGVGGAqvSAGQRPVFRLVWEKGGLKIMVAYWPYVPYDQSNF
WP_000836110_1_pir_______________PPQKAPKEHSWVDFAKKYLNMMDYLGTYFLPFYHSFTPIFQWYHPNINPYQRNEFKFQISFRVPVFRHILWTKGTLYLAYTQTDWFQIYNNPQSAPMRMINFMPELIYVYPINFKPFggNFSEIWIGWQHISNGVGGAqvSAGQRPVFRLVWEKGGLKIMVAYWPYVPYNQSNF
WP_000836616_1_pir_______________PPQKAPKEHSWVDFAKKYLNMMDYLGTYFLPFYHSFTPIFQWYHPNINPYQRNEFKFQISFRVPVFRHILWTKGTLYLAYTQTNWFQIYNDPQSAPMRMINFMPELIYVYPINFKPFggNFSEIWIGWQHISNGVGGAqvSAGQRPVFRLVWEKGGLKIMVAYWPYVPYNQSNF
WP_033779821_1_pir_______________PPQKAPKEHSWVDFAKKYLNMMDYLGTYFLPFYHSFTPIFQWYHPNINPYQRNEFKFQISFRVPVFRHILWTKGTLYLAYTQTNWFQIYNNPQSAPMRMINFMPELIYVYPINFKPFggNFSEIWIGWQHISNGVGGAqvSAGQRPVFRLVWEKGGLKIMVAYWPYVPYDQSNL
WP_000843899_1_pir_______________PPQKAPKEHSWVDFAKKYLNMMDYLGTYFLPFYHSFTPIFQWYHPNINPYQRNEFKFQISFRVPVFRHILWTKGTLYLAYTQTNWFQIYNDLQSAPMRMMNFMPELIYVYPINFKPFggNFSEIWIGWQHISNGVGGAqvSAGQRPVFRLVWEKGGLKIMVAYWPYVPYDQSNF
WP_017283890_1_pir_______________PPQKAPKEHSWVDFAKKYLNMMDYLGTYFLPFYHSFTPIFQWYHPNINPYQRNEFKFQISFRVPVFRHILWTKGTLYLAYTQTNWFQIYNNPQSAPMRMINFMPELIYVYPINFEPFggKFSEIWIGWQHISNGVGGAqvSARQRPVFRLVWEKGGLKIMVAYWPYVPYDQSNF
AFR51850_1_pir___________________PPQKAPKEHSWADFAKKYLNMMDYLGTYFLPFYHSFTPIFQWYHPNINPYQRNEFKFQISFRVPVFRHILWTKGTLYLAYTQTNWFQIYNDPQSAPMRMMNFMPELIYVYPINFKPFggNFSEIWIGWQHISNGVGGAqvSAGQRPVFRLVWEKGGLKIMVAYWPYVPYDQSNF
AFR51870_1_pir___________________PPQKAPKEHSWADFAKKYLNMMDYLGTYFLPFYHSFTPIFQWYHPNINPYQRNEFKFQISFRVPVFRHILWTKGTLYLAYTQTNWFQIYNDPQSAPMRMMNFMPELIYVYPINFKPFggNFSEIWIGWQHISNGVGGAqvSAGQRPVFRLVWEKGGLKIMVAYWPYVPYDQSNF
AFR51840_1_pir___________________PPQKAPKEHSWVDFAKKYLNMMDYLGTYFLPFYHSFTPIFQWYHPNINPYQRNEFKFQISFRVPVFRHILWTKGTLYLAYTQTDWFQIYNNPQSAPMRMINFMPEFIYVYPINFKPFggNFSEIWIGWQHISNGVGGAqvSAGQRPVFRLVWEKGGLKIMVAYWPYVPYDQSNF
WP_000836105_1_pir_______________PPQKAPKEHSWVDFAKKYLNMMDYLGTYFLPFYHSFTPIFQWYHPNINPYQRNEFKFQISFRVPVFRHILWTKGTLYLAYIQTNWFQIYNDPQSAPMRMMNFMPELIYVYPINFKPFggNFSEIWIGWQHISNGVGGAqvSAGQRPVFRLVWEKGGLKIMVAYWPYVPYDQSNF
WP_000676265_1_pir_______________PPQKAPKEHSWVDFAKKYLNMMDYLGTYFLPFYHSFTPIFQWYHPNINPYQRNEFKFQISFRVPVFRHILWTKGTLYLAYTQTNWFQIYNNPQSAPMRMINFMPELIYVYPINFEPFggKFSEIWIGWQHISNGVGGAqvSAGQRPVFRLVWEKGGLKIMVAYWPYVPYDQSNF
WP_048945489_1_pir_______________PPQKAPKEHSWVDFAKKYLNMMDYLGTYFLPFYHSFTPIFQWYHPNINPYQRNEFKFQISFRVPVFRHILWTKGTLYLAYTQTDWFQIYNNPQSAPMRMINFMPELIYVYPINFKPFggNFSEIWIGWQHISNGVGGAqvSAGQRPVFRLVWEKGGLKIMVAYWPYVPYDQSNF
AFR51830_1_pir___________________PPQKASKEHSWVDFAKKYLNMMDYLGTYFLPFYHSFTPIFQWYHPNINPYQRNEFKFQISFRVPVFRHILWTKGTLYLAYTQTNWFQIYNDPQSAPMRMMNFMPELIYVYPINFKPFggNFSEIWIGWQHISNGVGGAqvSAGQRPVFRLVWEKGGLKIMVAYWPYVPYDQSNF
AFR51756_1_pir___________________PPQKAPKEHSWVDFAKKYLNMMDYLGTYFLPFYHSFTPIYQWYHPNINPYQRNEFKFQISFRVPVFRHILWTKGTLYLAYTQTNWFQIYNDPQSAPMRMINFMPELIYVYPINFKPFggNFSEIWIGWQHISNGVGGAqvSAGQRPVFRLVWEKGGLKIMVAYWPYVPYDQSNF
AFR51808_1_pir___________________SPQKAPKEHSWVDFAKKYLNMMDYLGTYFLPFYHSFTPIFQWYHPNINPYQRNEFKFQISFRVPVFRHILWTKGTLYLAYTQTDWFQIYNNPQSAPMRMINFMPELIYVYPINFKPFggNFSEIWIGWQHISNGVGGAqvSAGQRPVFRLVWEKGGLKIMVAYWPYVPYDQSNF
WP_058904469_1_pir_______________PPQKAPKEYSWVDFAKKYLNMMDYLGTYFLPFYHSFTPIFQWYHPNINPYQRNEFKFQISFRVPVFRHILWTKGTLYLAYTQTDWFQIYNNPQSAPMRMINFMPELIYVYPINFKPFggNFSEIWIGWQHISNGVGGAqvSAGQRPVFRLVWEKGGLKIMVAYWPYVPYDQSNF
AFR51786_1_pir___________________PPQKAPKEHSWVDFAKKYLNMMDYLGTYFLPFYHSFTPIFQWYHPNINPYQRNEFKFQISFRVPVFRHILWTKGTLYLAYTQTNWFQIYNNPQSAPMRMINFMPELIYVYPINFEPFggKFSEIWIGWQHISNGVGGAqvSAGQRPVFRLVWEKGGLKIMVAYWPYVPYDQPNF
WP_033602947_1_pir_______________PPQKAPKEHSWVDFAKKYLNMMDYLGTYFLPFYHSFTPIFQWYHPNINPYQRNEFKFQISFRVPVFRHILWTKGTLYLAYTQTNWFQIYNNPQSAPMRMINFMPELIYVYPINFKPFggNFSEIWIGWQHISNGVGGAqvSAGQRPVFRLVWEKGGLKIMVAYWPYIPYYQSNF
OLQ57160_1_pir___________________PPQKAPKEHSWVDFAKKYLNMMDYLGTYFLPFYHSFTPIFQWYHPNINPYQRNEFKFQISFRVPVFRHILWTKGTLYLAYTQTDWFQIYNNPQSAPMRMINFMPELIYVYPINFEPFggKFSEIWIGWQHISNGVGGAqvNAGQRPVFRLVWEKGGLKIMVAYWPYVPYDQSNF
WP_000836131_1_pir_______________PPQKAPKEHSWVDFAKKYLNMMDYLGTYFLPFYHSFTPIFQWYHPNINPYQRNEFKFQISFRVPVFRHILWTKGTLYLAYTQTNWFQIYNNPQSAPMRMINFMPELIYVYPINFEPFggKFSEIWIGWQHISNGVGGAqvSAGQRPVFRLVWEKGGLKIMVAYWPYVPYDQSNF
AFR51740_1_pir___________________PPQKAPKEHSWVDFAKKYLNMMDYLGTYFLPFYHSFTPIFQWYHPNINPYQRNEFKFQISFRVPVFRHILWTKGTLYLAYTQTDWFQIYNDPQSAPMRMMNFMPELIYVYPINFKPFggNFSEIWIGWQHISNGVGRAqvSAGQRPVFRLVWEKGGLKIMVAYWPYVPYDQSNF
WP_000836149_1_pir_______________PPQKAPKEHSWVDFAKKYLNMMDYLGTYFLPFYHSFTPIFQWYHPNINPYQRNEFKFQISFRVPVFRHILWTKGTLYLAYTQTNWFQIYNDPQSAPMRMINFMPELIYVYPINFKPFggNFSEIWIGWQHISNGVGGAqvSAGQRPVFRLVWEKGGLKIMVAYWPYVPYDQSNF
WP_000843903_1_pir_______________PPQKAPKEHSWVDFAKKYLNMMDYLGTYFLSFYHSFTPIFQWYHPNINPYQRNEFKFQISFRVPVFRHILWTKGTLYLAYTQTNWFQIYNDPQSAPMRMINFMPELIYVYPINFKPFggNFSEIWIGWQHISNGVGGAqvSAGQRPVFRLVWEKGGLKIMVAYWPYIPYNQSNF
AFR51796_1_pir___________________PPQKAPKERSWVDFAKKYLNMMDYLGTYFLPFYHSFTPIFQWYHPNINPYQRNEFKFQISFRVPVFRHILWTKGTLYLAYTQTNWFQIYNDPQSAPMRMMNFMPELIYVYPINFKPFggNFSEIWIGWQHISNGVGGAqvSAGQRPVFRLVWEKGGLKIMVAYWPYVPYDQSNF
WP_033769094_1_pir_______________PPQKAPKEHSWVDFAKKYLNMMDYLGTYFLPFYHSFTPIFQWYHPNINPYQRNEFKFQISFRVPVFRHILWTKGTLYLAYTQTNWFQIYNDPQSAPMRMINFMPELIYVYPINFKPFggNFSEIWIGWQHISNGVGGAqvSAGQRPVFRLVWEKGGLKIMVAYWPYVPYVQSNF
AFR51768_1_pir___________________PPQKAPKEHSWVDFAKKYLNMMDYLGTYFLPFYHSFTPIFQWYHPNINPYQRNEFKFQISFRVPVFRHILWTKGMLYLAYTQTNWFQIYNDPQSAPMRMMNFMPELIYVYPINFKPFggNFSEIWIGWQHISNGVGGAqvSAGQRPVFRLVWEKGGLKIMVAYWPYVPYDQSNF
WP_000836088_1_pir_______________PPQKAPKEHSWVDFAKKYLNMMDYLGTYFLPFYHSFTPIFQWYHPNINPYQRNEFKFQISFRVPVFRHILWTKGMLYLAYTQTNWFQIYNDPQSAPMRMMNFMPELIYVYPINFKPFggNFSEIWIGWQHISNGVGGAqvSAGQRPVFRLVWEKGGLKIMVAYWPYVPYDQSNF
OLR43313_1_pir___________________PPQKAPKEHSWVDFAKKYLNMMDYLGTYFLPFYHSFTPIFQWYHPNINPYQRNEFKFQISFRVPVFRHILWTKGTLYLAYTQTDWFQIYNNPQSAPMRMINFMPELIYVYPINFKPFggNFSEIWIGWQHISNGVGGAqvSAGQRPVFRLVWEKGGLKIMVAYWPYVPYDQSSF
WP_033602014_1_pir_______________APQKAPKEHSWVDFAKKYLNMMDYLGTYFLPFYHSFTPIFQWYHPNINPYQRNEFKFQVSFRVPVFRHILWTKGTLYLAYTQTNWFQIYNDPQSAPMRMINFMPELIYVYPINFKPFggNFSEIWIGWQHISNGVGGAqvSAGQRPVFRLVWEKGGLKIMVAYWPYVPYDQSNF
WP_001966057_1_pir_______________APKKAPKEHSWVDFAKKYLNMMDYLGTYFLPFYHSFTPIFQWYHPNINPYQRNEFKFQISFRVPVFRHILWTKGTLYLAYTQTNWFQIYNDPQSAPMRMINFMPELIYVYPINFKPFggNFSEIWIGWQHISNGVGGAqvSAGQRPVFRLVWEKGGLKIMVAYWPYVPYDQSNF
WP_001973093_1_pir_______________APKKAPKEHSWVDFAKKYLNMMDYLGTYFLPFYHSFTPIFQWYHPNINPYQRNEFKFQISFRVPVFRHILWTKGTLYLAYTQTNWFQIYNDPQSAPMRMINFMPELIYVYPINFKPFggNFSEIWIGWQHISNGVGGAqvSAGQRPVFRLVWEKGGLKIMVAYWPYVPYDQSNF
WP_001986761_1_pir_______________APKKAPKEHSWVDFAKKYLNMMDYLGTYFLPFYHSFTPIFQWYHPNINPYQRNEFKFQISFRVPVFRHILWTKGTLYLAYTQTNWFQIYNDPQSAPMRMINFMPELIYVYPINFKPFggNFSEIWIGWQHISNGVGGAqvSAGQRPVFRLVWEKGGLKIMVAYWPYVPYDQSNF
WP_001966057_1_pir_______________APKKAPKEHSWVDFAKKYLNMMDYLGTYFLPFYHSFTPIFQWYHPNINPYQRNEFKFQISFRVPVFRHILWTKGTLYLAYTQTNWFQIYNDPQSAPMRMINFMPELIYVYPINFKPFggNFSEIWIGWQHISNGVGGAqvSAGQRPVFRLVWEKGGLKIMVAYWPYVPYDQSNF
WP_000843897_1_pir_______________PPQKAPKEHSWVDFAKKYLNMMDYLGTYFLPFYHSFTPIFQWYHPNINPYQRNEFKFQISFRVPVFRHILWTKGTLYLAYTQTNWFQIYNNPQSAPMRMINFMPELIYVYPINFKPFggNFSEIWIGWQHISNGVGGAqvSAGQRPVFRLVWEKGGLKIMVAYWPYVPYDQSNF
WP_000843901_1_pir_______________PPQKAPKEHSWVDFAKKYLNMMDYLGTYFLPFYHSFTPIFQWYHPNINPYQRNEFKFQISFRVPVFRHILWTKGTLYLAYTQTNWFQIYNDPQSAPMRMINFMPELIYVYPINFKPFggNFSEIWIGWQHISNGVGGAqvSAGQHPVFRLVWEKGGLKIMVAYWPYVPYDQSNF
AFR51933_1_pir___________________PPQKASKEHSWVDFAKKYLNMMDYLGTYFLPFYHSFTPIFQWYHPNINPYQRNEFKFQISFRVPVFRHILWTKGTLYLAYTQTDWFQIYNNPQSAPMRMINFMPELIYVYPINFKPFggNFSEIWIGWQHISNGVGGAqvSAGQRPVFRLVWEKGGLKIMVAYWPYVPYDQSNF
WP_000843898_1_pir_______________PPQKASKEHSWVDFAKKYLNMMDYLGTYFLPFYHSFTPIFQWYHPNINPYQRNEFKFQISFRVPVFRHILWTKGTLYLAYTQTDWFQIYNNPQSAPMRMINFMPELIYVYPINFKPFggNFSEIWIGWQHISNGVGGAqvSAGQRPVFRLVWEKGGLKIMVAYWPYVPYDQSNF
WP_050836487_1_pir_______________PPQKASKEHSWVDFAKKYLNMMDYLGTYFLPFYHSFTPIFQWYHPNINPYQRNEFKFQISFRVPVFRHILWTKGTLYLAYTQTDWFQIYNNPQSAPMRMINFMPELIYVYPINFKPFggNFSEIWIGWQHISNGVGGAqvSAGQRPVFRLVWEKGGLKIMVAYWPYVPYDQSNF
WP_052937098_1_pir_______________PPQKASKEHSWVDFAKKYLNMMDYLGTYFLPFYHSFTPIFQWYHPNINPYQRNEFKFQISFRVPVFRHILWTKGTLYLAYTQTDWFQIYNNPQSAPMRMINFMPELIYVYPINFKPFggNFSEIWIGWQHISNGVGGAqvSAGQRPVFRLVWEKGGLKIMVAYWPYVPYDQSNF
AFR51772_1_pir___________________PPQKAPKEHSWVDFAKKYLNMMDYLGTYFLPFYHSFTPIFQWYHPNINPYQRNEFKFQISFRVPVFRHIFWTKGTLYLAYTQTNWFQIYNNPQSAPMRMINFMPELIYVYPINFEPFggKFSEIWIGWQHISNGVGGAqvSAGQRPVFRLVWEKGGLKIMVAYWPYVPYDQSNF
AFR51751_1_pir___________________PPQKAPKGHSWVDFAKKYLNMMDYLGTYFLPFYHSFTPIFQWYHPNINPYQRNEFKFQISFRVPVFRHILWTKGTLYLAYTQTNWFQIYNDPQSAPMRMMNFMPELIYVYPINFKPFggNFSEIWIGWQHISNGVGGAqvSAGQRPVFRLVWEKGGLKIMVAYWPYVPYDQSNF
WP_033794331_1_pir_______________APQKAPKEHSWVDFAKKYLNMMDYLGTYFLPFYHSFTPIFQWYHPNINPYQRNEFKFQISFRVPVFRHILWTKGTLYLAYTQTNWFQIYNDPQSAPMRMINFMPEFIYVYPINFKPFggNFSEIWIGWQHISNGVGGAqvSAGQRPVFRLVWEKGGLKIMVAYWPYVPYDQSNF
AFR51759_1_pir___________________PPQKAPKEHSWVDFAKKYLNMMDYLGTYFLPFYHSFTPIFQWYHPNINPYQRNEFKFQISFRVPVFRHILWTKGTLYLAYTQTDWFQIYNDPQSAPMRMMNFMPELIYVYPINFKPFggNFSEIWIGWQHISNGVGGVqvSAGQRPVFRLVWEKGGLKIMVAYWPYVPYDQSNF
WP_001256629_1_pir_______________PPQKAPKEHSWVDFAKKYLNMMDYLGTYFLPFYHSFTPIFQWYHPNINPYQRNEFKFQISFRVPVFRHILWTKGTLYLAYTQTNWFQIYNNPQSAPMRMINFMPELIYVYPINFEPFggKFSEIWIGWQHISNGVGGVqvSAGQRPVFRLVWEKGGLKIMVAYWPYVPYDQSNF
WP_033589341_1_pir_______________PPQKAPKEHSWVDFAKKYLNMMDYLGTYFLPFYHSFTPIFQWYHPNINPYQRNEFKFQISFRVPVFRHILWTKGTLYLAYTQTDWFQIYNDPQSAPMRMMNFMPELIYVYPFNFKPFggNFSEIWIGWQHISNGVGGAqvSAGQRPVFRLVWEKGGLKIMVAYWPYVPYDQSNF
AFR51889_1_pir___________________PPQKAPKEHSWMDFAKKYLNMMDYLGTYFLPFYHSFTPIFQWYHPNINPYQRNEFKFQISFRVPVFRHILWTKGTLYLAYTQTDWFQIYNDPQSAPMRMINFMPELIYVYPINFKPFggNFSEIWIGWQHISNGVGGAqvSAGQRPVFRLVWEKGGLKIMVAYWPYVPYDQSNF
WP_024369531_1_pir_______________APQKAPKEHSWVDFVKKYLNMMDYLGTYFLPFYHSFTPIFQWYHPNINPYQRNEFKFQISFRVPVFRHILWTKGTLYLAYTQTDWFQIYNDPQSAPMRMINFMPELIYVYPINFKPFggNFSEIWIGWQHISNGVGGAqvSAGQRPVFRLVWEKGGLKIMVAYWPYVPYDQSNF
WP_024369531_1_pir_______________APQKAPKEHSWVDFVKKYLNMMDYLGTYFLPFYHSFTPIFQWYHPNINPYQRNEFKFQISFRVPVFRHILWTKGTLYLAYTQTDWFQIYNDPQSAPMRMINFMPELIYVYPINFKPFggNFSEIWIGWQHISNGVGGAqvSAGQRPVFRLVWEKGGLKIMVAYWPYVPYDQSNF
WP_000843805_1_pir_______________PPQKAPKEHSWVDFAKKYLNMMDYLGTYFLPFYHSFTPIFQWYHPNINPYQRNEFKFQISFRVPVFRHILWTKGTLYLAYTQTNWFQIYNNPQSAPMRMINFMPELIYVYPINFEPFggKFSEIWIGWQHISNGVGGAqvSAGQRPVFRLVWEKGDLKIMVAYWPYVPYDQSNF
AFR51742_1_pir___________________PPQKAPKEHSWVDFAKKYLNMMDYLGTYFLPFYHSFTPIFQWYHPNINPYQRNEFKFQISFRVPVFRHILWTKGTLYLAYTQTDWFQIYNNPQSAPMRMINFMPELIYVYPINFKPFggNFSEIWIGWQHISNGVGGAqvSAGQRPVFRLVWEKGGLKIMVAYWPYVPYDQSNF
AFR51746_1_pir___________________PPQKAPKEHSWVDFAKKYLNMMDYLGTYFLPFYHSFTPIFQWYHPNINPYQRNEFKFQISFRVPVFRHILWTKGTLYLAYTQTDWFQIYNNPQSAPMRMINFMPELIYVYPINFKPFggNFSEIWIGWQHISNGVGGAqvSAGQRPVFRLVWEKGGLKIMVAYWPYVPYDQSNF
AFR51755_1_pir___________________PPQKAPKEHSWVDFAKKYLNMMDYLGTYFLPFYHSFTPIFQWYHPNINPYQRNEFKFQISFRVPVFRHILWTKGTLYLAYTQTDWFQIYNNPQSAPMRMINFMPELIYVYPINFKPFggNFSEIWIGWQHISNGVGGAqvSAGQRPVFRLVWEKGGLKIMVAYWPYVPYDQSNF
WP_052919777_1_pir_______________PPQKAPKEHSWVDFAKKYLNMMDYLGTYFLPFYHSFTPIFQWYHPNINPYQRNEFKFQISFRVPVFRHILWTKGTLYLAYTQTDWFQIYNNPQSAPMRMINFMPELIYVYPINFKPFggNFSEIWIGWQHISNGVGGAqvSAGQRPVFRLVWEKGGLKIMVAYWPYVPYDQSNF
AFR51800_1_pir___________________PPQKAPKEYSWVDFAKKYLNMMDYLGTYFLPFYHSFTPIFQWYHPNINPYQRNEFKFQISFRVPVFRHILWTKGTLYLAYTQTNWFQIYNDPQSAPMRMMNFMPELIYVYPINFKPFggNFSEIWIGWQHISNGVGGAqvSAGQRPVFRLVWEKGGLKIMVAYWPYVPYDQSNF
AFR51928_1_pir___________________SPQKAPKEHSWVDFAKKYLNMMDYLGTYFLPFYHSFTPIFQWYHPNINPYQRNEFKFQISFRVPVFRHILWTKGTLYLAYTQTDWFQIYNNPQSAPMRMINFMPELIYVYPINFKPFggNFSEIWIGWQHISNGVGGAqvSAGQRPVFRLVWEKGGLKIMVAYWPYVPYDESNF
AFR51802_1_pir___________________SPQKAPKEHSWVDFAKKYLNMMDYLGTYFLPFYHSFTPIFQWYHPNINPYQRNEFKFQISFRVPVFRHILWTKGTLYLAYTQTNWFQIYNDPQSAPMRMMNFMPELIYVYPINFKPFggNFSEIWIGWQHISNGVGGAqvSAGQRPVFRLVWEKGGLKIMVAYWPYVPYDQSNF
```

```
AFR51882_1_pir________________SPQKAPKEHSWVDFAKKYLNMMDYLGTYFLPFYHSFTPIFQWYHPNINPYQRNEFKFQISFRVPVFRHILWTKGTLYLAYTQTNWFQIYNDPQSAPMRMMNFMPELIYVYPINFKPFggNFSEIWIGWQHISNGVGGAqvSAGQRPVFRLVWEKGGLKIMVAYWPYVPYDQSNF
WP_033736993_1_pir____________PPQKAPKEHSWVDFAKKYLNMMDYLGTYFLPFYHSFTPIFQWYHPNINPYQRNEFKFQISFRVPVFRHILWTKGTLYLAYTQTDWFQIYNNPQSAPMRMINFMPELIYVYPINFKPFggNFSEIWIGWQHISNGVGGAqvSAGQRPVFRLVWEKGGLKIMVAYWPYVPYNQSNF
WP_021582091_1_pir____________PPQKAPKEYSWVDFAKKYLNMMDYLGTYFLPFYHSFTPIFQWYHPNINPYQRNEFKFQISFRVPVFRHILWTKGTLYLAYTQTNWFQIYNDPQSAPMRMINFMPELIYVYPINFKPFggNFSEIWIGWQHISNGVGGVqvSAGQRPVFRLVWEKGGLKIMVAYWPYIPYDQSNF
WP_021582091_1_pir____________PPQKAPKEYSWVDFAKKYLNMMDYLGTYFLPFYHSFTPIFQWYHPNINPYQRNEFKFQISFRVPVFRHILWTKGTLYLAYTQTNWFQIYNDPQSAPMRMINFMPELIYVYPINFKPFggNFSEIWIGWQHISNGVGGVqvSAGQRPVFRLVWEKGGLKIMVAYWPYIPYDQSNF
AFR51748_1_pir________________PPQKAPKEHSWVDFAKKYLNMMDYLGTYFLPFYHSFTPIFQWYHPNINPYQRNEFKFQISFRVPVFRHILWTKGTFYLAYTQTNWFQIYNDPQSAPMRMMNFMPELIYVYPINFKPFggNFSEIWIGWQHISNGVGGAqvSAGQRPVFRLVWEKGGLKIMVAYWPYVPYDQSNF
AFR51728_1_pir________________PPQKAPKEHSWVDFAKKYLNMMDYLGTYFLPFYHSFTPIFQWYHPNINPYQRNEFKFQISFRVPVFRHILWTKGTLYLAYTQTNWFQIYNDPQSAPMRMMNFMPELIYVYPINFKPFggNFSEIWIGWQHISNGVGGAqvSAGQRPVFRLVWEKGGLKIMVAYWPYVPYVQSNF
WP_001950859_1_pir____________APKKAPKEHSWVDFAKKYLNMMDYLGTYFLPFYHSFTPIFQWYHPNINPYQRNEFKFQISFRVPVFRHILWTKGTLYLAYTQTNWFQIYNDPQSAPMRMMNFMPELIYVYPINFKPFggNFSEIWIGWQHISNGVGGAqvSAGQRPVFRLVWEKGGLKIMVAYWPYVPYDQSNF
WP_050826293_1_pir____________PPQKAPKEHSWVDFAKKYLNMMDHLGTYFLPFYHSFTPIFQWYHPNINPYQRNEFKFQISFKVPVFRHILWTKGTLYLAYTQTDWFQIYNNPQSAPMRMINFMPELIYVYPINFKPFggNFSEIWIGWQHISNGVGGAqvSAGQRPVFRLVWEKGGLKIMVAYWPYVPYGQSNF
WP_050826293_1_pir____________PPQKAPKEHSWVDFAKKYLNMMDHLGTYFLPFYHSFTPIFQWYHPNINPYQRNEFKFQISFKVPVFRHILWTKGTLYLAYTQTDWFQIYNNPQSAPMRMINFMPELIYVYPINFKPFggNFSEIWIGWQHISNGVGGAqvSAGQRPVFRLVWEKGGLKIMVAYWPYVPYGQSNF
WP_000836096_1_pir____________PPQKAPKEHSWVDFAKKYLNMMDYLGTYFLPFYHSFTPIFQWYHPNINPYQRNEFKFQISFRVPVFRHILWTKGTLYLAYTQTNWFQIYNDPQSAPMRMMNFMPELIYVYPINFKPFggNFSEIWIGWQHISNGVGGAqvSAGQRPVFRLVWEKGGLKIMVAYWPYVPYDQSSF
AFR51727_1_pir________________PPQKAPKEHSWVDFAKKYLNMMDYLGTYFLPFYHSFTPIFQWYHPNINPYQRNEFKFQISFRVPVFRHILWTKGTLYLAYTQTNWFQIYNDPQSAPMRMMNFMPELIYVYPINFKPFggNFSEIWIGWQHISNGVGGAqvSAGQHPVFRLVWEKGGLKIMVAYWPYVPYDQSNF
AFR51867_1_pir________________PPQKAPKEHSWVDFAKKYLNMMDYLGTYFLPFYHSFTPIFQWYHPNINPYQRNEFKFQISFRVPVFRHILWTKGTLYLANTQTNWFQIYNDPQSAPMRMMNFMPELIYVYPINFKPFggNFSEIWIGWQHISNGVGGAqvSAGQRPVFRLVWEKGGLKIMVAYWPYVPYDQSNF
AFR51815_1_pir________________PPQKASKEHSWVDFAKKYLNMMDYLGTYFLPFYHSFTPIFQWYHPNINPYQRNEFKFQISFRVPVFRHILWTKGTLYLAYTQTDWFQIYNNPQSAPMRMMNFMPELIYVYPINFKPFggNFSEIWIGWQHISNGVGGAqvSAGQRPVFRLVWEKGGLKIMVAYWPYVPYDQSNF
WP_033753692_1_pir____________APQKAPKEHSWVDFAKKYLNMMDYLGTYFLPFYHSFTPIFQWYHPNINPYQRNEFKFQISFRVPVFRHILWTKGTLYLAYTQTNWFQIYNDPQSAPMRMINFMPELIYVYPINFKPFggNFSEIWIGWQHISNGVGGAqvSAGQRPVFRLVWENGGLKIMVAYWPYVPYDQSNF
WP_033753692_1_pir____________APQKAPKEHSWVDFAKKYLNMMDYLGTYFLPFYHSFTPIFQWYHPNINPYQRNEFKFQISFRVPVFRHILWTKGTLYLAYTQTNWFQIYNDPQSAPMRMINFMPELIYVYPINFKPFggNFSEIWIGWQHISNGVGGAqvSAGQRPVFRLVWENGGLKIMVAYWPYVPYDQSNF
AFR51835_1_pir________________PPQKAPKEYSWVDFAKKYLNMMDYLGTYFLPFYHSFTPIFQWYHPNINPYQRNEFKFQISFRVPVFRHILWTKGTLYLAYTQTDWFQIYNDPQSAPMRMMNFMPELIYVYPINFKPFggNFSEIWIGWQHISNGVGGAqvSAGQRPVFRLVWEKGGLKIMVAYWPYVPYDQSNF
AFR51905_1_pir________________PPQKAPKEYSWVDFAKKYLNMMDYLGTYFLPFYHSFTPIFQWYHPNINPYQRNEFKFQISFRVPVFRHILWTKGTLYLAYTQTDWFQIYNDPQSAPMRMMNFMPELIYVYPINFKPFggNFSEIWIGWQHISNGVGGAqvSAGQRPVFRLVWEKGGLKIMVAYWPYVPYDQSNF
EJB29429_1_pir________________APQKAPKEHSWVDFAKKYLNMMDYLGTYFLPFYHSFTPIFQWYHPNINPYQRNEFKFQISFRVPVFRHILWTKGTLYLAYTQTNWFQIYNDPQSAPMRMMNFMPELIYVHPINFKPFggNFSEIWIGWQHISNGVGGAqvSAGQRPVFRLVWEKGGLKIMVAYWPYVPYDQSNF
WP_000836087_1_pir____________APQKAPKEHSWVDFAKKYLNMMDYLGTYFLPFYHSFTPIFQWYHPNINPYQRNEFKFQISFRVPVFRHILWTKGTLYLAYTQTNWFQIYNDPQSAPMRMMNFMPELIYVHPINFKPFggNFSEIWIGWQHISNGVGGAqvSAGQRPVFRLVWEKGGLKIMVAYWPYVPYDQSNF
EJB29429_1_pir________________APQKAPKEHSWVDFAKKYLNMMDYLGTYFLPFYHSFTPIFQWYHPNINPYQRNEFKFQISFRVPVFRHILWTKGTLYLAYTQTNWFQIYNDPQSAPMRMMNFMPELIYVHPINFKPFggNFSEIWIGWQHISNGVGGAqvSAGQRPVFRLVWEKGGLKIMVAYWPYVPYDQSNF
AFR51896_1_pir________________PPQKAPKEHSWVDFAKKYLNMMDYLGTYFLPFYHSFTPIFQWYHPNINPYQRNEFKFQISFRVPVFRHILWTKGTLYLAYTQTDWFQIYNDPQSAPMRMMNFMPELIYVYPINFKPFggNFSEIWIGWQHISNGVGGAqvSAGQRPVFRLVWEKGGLKIMVAYWPYVPYVQSNF
WP_058055080_1_pir____________PPQKAPKEHSWADFAKKYLNMMDYLGTYFLPFYHSFTPIFQWYHPNINPYQRNEFKFQISFRVPVFRHILWTKGTLYLAYTQTNWFQIYNNPQSAPMRMINFMPELIYVYPINFEPFggKFSEIWIGWQHISNGVGGAqvSAGQRPVFRLVWEKGGLKIMVAYWPYVPYDQSNF
OLR40803_1_pir________________PPQKAPKEHSWADFAKKYLNMMDYLGTYFLPFYHSFTPIFQWYHPNINPYQRNEFKFQISFRVPVFRHILWTKGTLYLAYTQTNWFQIYNNPQSAPMRMINFMPELIYVYPINFEPFggKFSEIWIGWQHISNGVGGAqvSAGQRPVFRLVWEKGGLKIMVAYWPYVPYDQSNF
WP_000836137_1_pir____________PPQKAPKEHSWVDFAKKYLNMMDYLGTYFLPFYHSFTPIFQWYHPNINPYQRNEFKFQISFRVPVFRHILWTKGTLYLAYTQTDWFQIYNDPQSAPMRMMNFMPELIYVYPINFKPFggNFSEIWIGWQHISNGVGGAqvSAGQHPVFRLVWEKGGLKIMVAYWPYVPYDQSNF
WP_001956566_1_pir____________PPQKATKDHSWVDFAKKYLNMMDYLGTYFLPFYHSFTPIFQWYHPNINPYQRNEFKFQISFRVPVFRHILWTKGTLYLAYTQTNWFQIYNDPQSAPMRMINFMPELIYVYPINFKPFggNFSEIWIGWQHISNGVGGAqvSAGQRPVFRLVWEKGGLKIMVAYWPYVPYDQSNF
AFR51778_1_pir________________PPQKASKEHSWVDFAKKYLNMMDYLGTYFLPFYHSFTPIFQWYHPNINPYQRNEFKFQISFRVPVFRHILWTKGTLYLAYTQTNWFQIYNNPQSAPMRMINFMPELIYVYPINFEPFggKFSEIWIGWQHISNGVGGAqvSAGQRPVFRLVWEKGGLKIMVAYWPYVPYDQSNF
WP_000836136_1_pir____________PPQKAPKEHSWVDFAKKYLNMMDYLGTYFLPFYHSFTPIFQWYHPNINPYQRNEFKFQISFRVPVFKHILWTKGTLYLAYTQTDWFQIYNNPQSAPMRMMNFMPELIYVYPINFKPFggNFSEIWIGWQHISNGVGGAqvSAGQRPVFRLVWEKGGLKIMVAYWPYVPYDQSNF
WP_024114803_1_pir____________SPQKAPKEHSWVDFAKKYLNMMDYLGTYFLPFYHSFTPIFQWYHPNINPYQRNEFKFQISFRVPVFRHILWTKGTLYLAYTQTDWFQIYNNPQSAPMRMINFMPELIYVYPINFKPFggNFSEIWIGWQHISNGVGGAqvSAGQRPVFRLVWEKGDLKIMVAYWPYVPYDQSNF
WP_000676266_1_pir____________SPQKAPKEHSWVDFAKKYLNMMDYLGTYFLPFYHSFTPIFQWYHPNINPYQRNEFKFQISFRVPVFRHILWTKGTLYLAYTQTNWFQIYNDPQSAPMRMINFMPELIYVYPINFEPFggKFSEIWIGWQHISNGVGGAqvSAGQRPVFRLVWEKGGLKIMVAYWPYVPYDQSNF
AFR51836_1_pir________________PPQKAPKEHSWMDFAKKYLNMMDYLGTYFLPFYHSFTPIFQWYHPNINPYQRNEFKFQISFRVPVFRHILWTKGTLYLAYTQTDWFQIYNDPQSAPMRMINFMPELIYVYPINFKPFggNFSEIWIGWQHISNGVGGAqvSAGQRPVFRLVWEKGGLKIMVAYWPYVPYDQSNF
WP_000836095_1_pir____________PPQKAPKEHSWVDFAKKYLNMMDYLGTYFLPFYHSFTPIFQWYHPNINPYQRNEFKFQISFRVPVFRHILWTKGTLYLAYTQTDWFQIYNNPQSAPMRMINFMPELIYVYPINFKPFggNFSEIWIGWQHISNGVGGAqvSARQRPVFRLVWEKGGLKIMVAYWPYVSYDQSNF
AFR51760_1_pir________________PPQKAPKEHSWVDFAKKYLNMMDYLGTYFLPFYHSFTPIFQWYHPNINPYQRNEFKFQISFRVPVFRHILWTKGTLYLAYTQTNWFQIYNNPQSAPMRMINFMPELIYVYPINFEPFggKFSEIWIGWQHISNGVGGAqvSAGQRPVFRLVWEKGGLKIMVAYWPYVPYGQSNF
WP_021306849_1_pir____________PPQKAPKEHSWVDFAKKYLNMMDYLGTYFLPLYHSFTPIFQWYHPNINPYQRNEFKFQISFRVPVFRHILWTKGTLYLAYTQTNWFQIYNNPQSAPMRMINFMPELIYVYPINFEPFggKFSEIWIGWQHISNGVGGAqvSAGQRPVFRLVWEKGGLKIMVAYWPYVPYDQSNF
WP_000836121_1_pir____________PPQKAPKEHSWVDFAKKYLNMMDYLGTYFLPFYHSFTPIFQWYHPNINPYQRNEFKFQISFRVPVFRHILWTKGTLYLAYTQTNWFQIYNDPQSAPMRMINFMPELIYVYPINFEPFggKFSEIWIGWQHISNGVGGAqvSAGQRPVFRLVWEKGGLKIMVAYWPYVPYDQSNF
WP_021301674_1_pir____________SPQKAPKEHSWVDFAKKYLNMMDYLGTYFLPFYHSFTPIFQWYHPNINPYQRNEFKFQISFRVPVFRHILWTKGTLYLAYTQTNWFQIYNNPQSAPMRMINFMPELIYVYPINFEPFggKFSEIWIGWQHISNGVGGAqvNAGQRPVFRLVWEKGGLKIMVAYWPYVPYDQSNF
AFR51788_1_pir________________SPQKAPKEHSWVDFAKKYLNMMDYLGTYFLPFYHSFTPIFQWYHPNINPYQRNEFKFQISFRVPVFRHILWTKGTLYLAYTQTDWFQIYNNPQSAPMRMINFMPELIYVYPINFKPFggNFSEIWIGWQHISNGVGGAqvSAGQRPVFRLVWEKGGLKIMVAYWPYVPYNQSNF
WP_000836113_1_pir____________PPQKAPKEHFWVDFAKKYLNMMDYLGTYFLPFYHSFTPIFQWYHPNINPYQRNEFKFQISFRVPVFRHILWTKGTLYLAYTQTNWFQIYNDPQSAPMRMMNFMPELIYVYPINFKPFggNFSEIWIGWQHISNGVGGAqvSAGQRPVFRLVWEKGGLKIMVAYWPYVPYDQSNF
AFR51779_1_pir________________PPQKAPKEHSWVDFAKKYLNMMDYLGTYFLPFYHSFTPIFQWYHPNINPYQRNEFKFQISFRVPVFRHILWTKGTLYLAYTQTNWFQIYNNPQSAPMRMINFMPELIYIYPINFEPFggKFSEIWIGWQHISNGVGGAqvSAGQRPVFRLVWEKGGLKIMVAYWPYVPYDQSNF
WP_000836115_1_pir____________PPQKAPKEHSWVDFAKKYLNMMDYLGTYFLPFYHSFTLIFQWYHPNINPYQRNEFKFQISFRVPVFRHILWTKGTLYLAYTQTDWFQIYNNPQSAPMRMINFMPELIYVYPINFKPFggNFSEIWIGWQHISNGVGGAqvSAGQRPVFRLVWEKGGLKIMVAYWPYVPYDQSNF
AFR51771_1_pir________________PPQKAPKEHSWVDFAKKYLNMMDYLGTYFLPFYHSFTPIFQWYHPNINPYQRNEFKFQISFRVPVFRHILWTKGTLYLAYTQTNWFQIYNNPQSAPMRMINFMPELIYVYPINFEPFggKFSEIWIGWQHISNGVGGAqvSAGQRPVFRLVWEKGGLKIMVAYWPYVPYDQSNF
WP_000836134_1_pir____________PPQKAPKEHSWVDFAKKYLNMMDYLGTYFLPFYHSFTPIFQWYYPNINPYQRNEFKFQISFRVPVFRHILWTKGTLYLAYTQTNWFQIYNNPQSAPMRMINFMPELIYVYPINFEPFggKFSEIWIGWQHISNGVGGAqvSAGQRPVFRLVWEKGGLKIMVAYWPYVPYNQSNF
WP_000836127_1_pir____________PPQKAPKEHSWVDFAKKYLNMMDYLGTYFLPFYHSFTPIFQWYHPNINPYQRNEFKFQISFRVPVFRHILWTKGTLYLAYTQTNWFQIYNNPQSAPMRMINFMPELIYIYPINFEPFggKFSEIWIGWQHISNGVGGAqvSAGQRPVFRLVWEKGGLKIMVAYWPYVPYDQSNF
AFR51916_1_pir________________PPQKAPKEHSWVDFAKKYLNMMDYLGTYFLPFYHSFTPIFQWYHPNINPYQRNEFKFQISFRVPVFRHILWTKGTLYLAYTQTNWFQIYNDPQSAPMRMMNFMPELIYVYPINFKPFggNFSEIWIGWQHISNGVGGTqvSAGQRPVFRLVWEKGGLKIMVAYWPYVPYDQSSF
WP_060760079_1_pir____________PPQKAPKEHSWVDFAKKYLNMMDYLGTYFLPFYHSFTPIFQWYHPNINPYQRNEFKLQISFRVPVFRHILWTKGTLYLAYTQTNWFQIYNNPQSAPMRMINFMPELIYVYPINFEPFggKFSEIWIGWQHISNGVGGAqvSAGQRPVFRLVWEKGGLKIMVAYWPYVPYDQSNF
WP_050832777_1_pir____________PPQKAPKEYSWVDFAKKYLNMMDYLGTYFLPFYHSFTPIFQWYHPNINPYQRNEFKFQISFRVPVFRHILWTKGTLYLAYTQTNWFQIYNNPQSAPMRMINFMPELIYVYPINFEPFggKFSEIWIGWQHISNGVGGAqvSAGQRPVFRLVWEKGGLKIMVAYWPYVPYDQSNF
AFR51789_1_pir________________PPQKAPKEHSWVDFAKKYLNMMDYLGTYFLPFYHSFTPIFQWYHPNINPYQRNEFKFQISFRVPVFRNILWTKGTLYLAYTQTNWFQIYNNPQSAPMRMINFMPELIYIYPINFEPFggKFSEIWIGWQHISNGVGGAqvSAGQRPVFRLVWEKGGLKIMVAYWPYVPYDQSNF
WP_001953625_1_pir____________APQKAPKEHSWVDFAKKYLNMMDYLGTYFLPFYHSFTPIFQWYHPNINPYQRNEFKFQISFRVPVFRHILWTKGTLYLAYTQTNWFQIYNDPQSAPMRMINFMPELIYVYPINFKPFggNFSEIWIGWQHISNGVGGAqvSAGQRPVFRLVWENGGLKIMVAYWPYVPYDQSNF
WP_033123738_1_pir____________PPQKAPKEHSWVDFAKKYLNMMDYLGTYFLPFYHSFTPIFQWYHPNINPYQRNEFKFQISFRVPVFRHILWTKGTFYLAYTQTNWFQIYNNPQSAPMRMINFMPELIYVYPINFEPFggKFSEIWIGWQHISNGVGGAqvSAGQRPVFRLVWEKGGLKIMVAYWPYVPYDQSNF
WP_020982246_1_pir____________PPQKAHKEHSWVDFAKKYLNMMDYLGTYFLPFYHSFTPIFQWYHPNINPYQRNEFKFQISFRVPVFRHILWTKGTLYLAYTQTNWFQIYNNPQSAPMRMINFMPELIYVYPINFEPFggKFSEIWIGWQHISNGVGGAqvSAGQRPVFRLVWEKGGLKIMVAYWPYVPYDQSNF
WP_001956149_1_pir____________APQKATKEHSWVDFAKKYLNMMDYLGTYFLPFYHSFTPIFQWYHPNINPYQRNEFKFQISFRVPVFRHILWTRGTLYLAYTQTNWFQIYNDPQSAPMRMINFMPELIYVYPINFKPFggNFSEIWIGWQHISNGVGGAqvSAGQRPVFRLVWEKGGLKIMVAYWPYVPYDQSNF
WP_000836100_1_pir____________PPQKAPKEHSWVDFAKKYLNMMDYLGTYFLPFYHSFTPIFQWYHPNINPYQRNEFKFQISFRVPVFRHILWTKGTLYLAYTQTNWFQIYNNPQSAPMRMINFMPELIYVYPINFEPFggKFSEIWIGWQHISNGVGGAqvSAGQRPVFRLVWEKGGLKIMVAYWPYVPYVQSNF
OLR51663_1_pir________________PPQKAPKEHSWVDFAKKYLNMMDYLGTYFLPFYHSFTPIFQWYHPNINPYQRNEFKFQISFRVPVFRHILWTKGTLYLAYTQTNWFQIYNNPQSAPMRMINFMPELIYVYPINFEPFggKFSEIWIGWQHISNGVGGAqvSAGQRPVFRLVWEKGGLKIMVAYWPYVPYVQSNF
OLR49643_1_pir________________PPQKAPKEHSWVDFAKKYLNMMDYLGTYFLPFYHSFTPIFQWYHPNINPYQRNEFKFQISFRVPVFMHILWTKGTLYLAYTQTNWFQIYNNPQSAPMRMINFMPELIYVYPINFEPFggKFSEIWIGWQHISNGVGGAqvSAGQRPVFRLVWEKGGLKIMVAYWPYVPYDQSNF
AFR51828_1_pir________________PPQKAPKEYSWVDFAKKYLNMMDYLGTYFLPFYHSFTPIFQWYHPNINPYQRNEFKFQISFRVPVFRHILWTKGTLYLAYTQTNWFQIYNDPQSAPMRMMNFMPELIYVYPINFKPFggNFSEIWIGWQHISNGVGGAqvSAGQRPVFRLVWEKGDLKIMVAYWPYVPYDQSNF
WP_000836133_1_pir____________PPQKAPKEHSWVDFAKKYLNMMDYLGTYFLPFYHSFTPIFQWYHPNINPYQRNEFKFQISFRVPVFRHILWTKGTLYLAYTQTNWFQIYNNPQSAPMRMINFMPKLIYVYPINFEPFggKFSEIWIGWQHISNGVGGAqvSAGQRPVFRLVWEKGGLKIMVAYWPYVPYDQSNF
WP_024116348_1_pir____________PPQKAPKEHSWVDFAKKYLNMMDYLGTYFLPFYHSFTPIFQWYHPNINPYQRNEFKFQISFRVPVFRHILWTKGTLYLAYTQTNWFQIYNNPQSAPMCMINFMPELIYVYPINFEPFggKFSEIWIGWQHISNGVGGAqvSAGQRPVFRLVWEKGGLKIMVAYWPYVPYDQSNF
WP_000836107_1_pir____________PPQKATKDHSWVDFAKKYLNMMDYLGTYFLPFYHSFTPIFQWYHPNINPYQRNEFKFQISFRVPVFRHILWTKGTLYLAYTQTNWFQIYNDPQSAPMRMINFMPELIYVYPINFKPFggNFSEIWIGWQHISNGVGGAqvSAGQRPVFRLVWEKGGLKIMVAYWPYVPYDQSNF
WP_073470282_1_pir____________PPQKAPKEHSWVDFAKKYLNMMDYLGTYFLPLYHSFTPIFQWYHPNINPYQRNEFKFQISFRVPVFRHILWTKGTLYLAYTQTNWFQIYNNPQSAPMRMINFMPELIYVYPINFEPFggKFSEIWIGWQHISNGVGGAqvSARQRPVFRLVWEKGGLKIMVAYWPYVPYDQSNF
WP_000836608_1_pir____________PPQKAPKEHSWVDFAKKYLNMMDYLGTYFLPFYHSFTPIFQWYHPNINPYQRNEFKFQISFRVPVFRHILWTKGTLYLAYTQTDWFQIYNNPQSAPMRMMNFMPELIYVYPINFKPFggNFSEIWIGWQHISNGVGGAqvSAGQRPVFRLVWEKGGLKIMVAYWPYVPYDQSNF
WP_064437711_1_pir____________PPQKAPKEHSWVDFAKKYLNMMDYLGTYFMPFYHSFTPIYQWYHPNINPYQRNEFKFQISFRVPVFRHILWTKGTLYLAYTQTDWFQIYNNPQSAPMRMMNFMPELIYVYPINFKPFggNFSEIWIGWQHISNGVGGAqvSAGQRPVFRLVWEKGGLKIMVAYWPYVPYDQSNF
AFR51878_1_pir________________PPQKAPKEHSWVDFAKKYLNMMDYLGTYFLPFYHSFTPIFQWYHPNINPYQRNEFKFQISFRVPVFRHILWTKGTLYLAYTQTDWFQIYNNPQSAPMRMINFMPELIYVYPINFKPFggNFSEIWIGWQHISNGVGGVqvSAGQRPVFRLVWEKGGLKIMVAYWPYVPYDQSNF
WP_000836093_1_pir____________PPQKAPKEHSWVDFAKKYLNMMDHLGTYFLPFYHSFTPIFQWYHPNINPYQRNEFKFQISFRVPVFRHILWTKGMLYLAYTQTDWFQIYNNPQSAPMRMINFMPELIYVYPINFKPFggNFSEIWIGWQHISNGVGGAqvSAGQRPVFRLVWEKGGLKIMVAYWPYVPYDQSNF
WP_000836114_1_pir____________PPQKAPKEHSWADFAKKYLNMMDYLGTYFLPFYHSFTPIFQWYHPNINPYQRNEFKFQISFRVPVFRHILWTKGTLYLAYTQTNWFQIYNDPQSAPMRMINFMPELIYVYPINFEPFggKFSEIWIGWQHISNGVGGAqvSAGQRPVFRLVWEKGGLKIMVAYWPYVPHDQSNF
OLQ25551_1_pir________________PPQKAPKEHSWADFAKKYLNMMDYLGTYFLPFYHSFTPIFQWYHPNINPYQRNEFKFQISFRVPVFRHILWSKGTLYLAYTQTNWFQIYNNPQSAPMRMINFMPELIYVYPINFEPFggNFSEIWIGWQHISNGVGGAqvSAGQRPVFRLVWEKGGLKIMVAYWPYVPYDQSNF
WP_021300265_1_pir____________PPQKAPKEHSWVDFAKKYLNMMDYLGTYFLPFYHSFTPIFQWYHPNINPYQRNEFKFQISFRVPVFRHILWTKGTLYLAYTQTDWFQIYNNPQSAPMRMINFMPELIYIYPINFEPFggKFSEIWIGWQHISNGVGGAqvSAGQRPVFRLVWEKGDLKIMVAYWPYVPYDQSNF
WP_021300265_1_pir____________PPQKAPKEHSWVDFAKKYLNMMDYLGTYFLPFYHSFTPIFQWYHPNINPYQRNEFKFQISFRVPVFRHILWTKGTLYLAYTQTDWFQIYNNPQSAPMRMINFMPELIYIYPINFEPFggKFSEIWIGWQHISNGVGGAqvSAGQRPVFRLVWEKGDLKIMVAYWPYVPYDQSNF
OLQ57136_1_pir________________PPQKASKEHSWVDFAKKYLNMMDYLGTYFLPFYHSFTPIFQWYHPNINPYQRNEFKFQISFRVPVFRHILWTKGTLYLAYTQTDWFQIYNNPQSAPMRMINFMPELIYVYPINFKPFggNFSEIWIGWQHISNGVGGAqvSAGQRPVFRLVWEKGGLKIMVAYWPYVPYDQSNF
AFR51872_1_pir________________PPQKAPKEHSWVDFAKKYLSMMDYLGTYFLPFYHSFTPIFQWYHPNINPYQRNEFKFQISFRVPVLRHILWTKGTLYLAYTQTDWFQIYNDPQSAPMRMMNFMPELIYVYPINFKPFggNFSEIWIGWQHISNGVGGAqvSAGQRPVFRLVWEKGGLKIMVAYWPYVPYDQSNF
WP_000836124_1_pir____________PPQKAPKEHSWVDFAKKYLNMMDYLGTYFLPFYHSFTPIFQWYHPNINPYQRNEFKFQISFRVPVFRHILWTKGTLYLAYTQTNWFQIYNDPQSAPMRMINFMPELIYVYPINFKPFggNFSEIWIGWQHISNGVGGAqvSAGNALCFVLVWEKGGLKIMVAYWPYVPYDQSNF
WP_000836124_1_pir____________PPQKAPKEHSWVDFAKKYLNMMDYLGTYFLPFYHSFTPIFQWYHPNINPYQRNEFKFQISFRVPVFRHILWTKGTLYLAYTQTNWFQIYNDPQSAPMRMINFMPELIYVYPINFKPFggNFSEIWIGWQHISNGVGGAqvSAGNALCFVLVWEKGGLKIMVAYWPYVPYDQSNF
WP_003015563_1_pir____________PPQKASKEHSWVDFAKKYLNMMDYLGTYFLPFYHSFTPIFQWYHPNINPYQRNEFKFQISFRVPVFRHILWTKGTLYLAYTQTDWFQIYNNPQSAPMRMINFMPELIYVYPINFKPFggNFSEIWIGWQHISNGVGGAqvSAGQRPVFRLVWEKGGLKIMVAYWPYVPYNQSNF
AFR51743_1_pir________________PPQKAQKDHSWVDFAKKYLNMMDYLGTYFLPFYHSFTPIFQWYHPNINPYQRNEFKFQISFRVPVFRHILWTKGTLYLAYTQTNWFQIYNDPQSAPMRMMNFMPELIYVYPINFKPFggNFSEIWIGWQHISNGVGGAqvSAGQRPVFRLVWEKGGLKIMVAYWPYVPYDQSNF
WP_033603222_1_pir____________APQKAPKEHSWVDFAKKYLNMMDYLGTYFLPFYHSFTPIFQWYHPNINPYQRNEFKFQVSFRVPVFRHILWTKGTLYLAYTQTNWFQIYNDPQSAPMRMINFMPELIYVYPINFKPFggNFSEIWIGWQHISNGVGGAqvSAGQRPVFRLVWEKGGLKIMVAYWPYVLYDQSNF
```

```
AFR51774_1_pir________________PPQKAPKEYSWVDFAKKYLNMMDYLGTYFLPFYHSFTPIFQWYHPNINPYQRNEFKFQISFRVPVFRHILWTKGTLYLAYTQTNWFQIYNNPQSAPMRMINFMPELIYVYPINFEPFggKFSEIWIGWQHISNGVGGAqvSAGQRPVFRLVWEKGGLKIMVAYWPYVPYDQSNF
WP_033122072_1_pir____________PPQKAPKEHSLADFAKKYLNMMDYLGTYFLPFYHSFTPIFQWYHPNINPYQRNEFKFQISFRVPVFRHILWTKGTLYLAYTQTNWFQIYNDPQSAPMRMINFMPELIYVYPINFEPFggKFSEIWIGWQHISNGVGGAqvSAGQRPVFRLVWEKGGLKIMVAYWPYVPYDQSNF
AFR51814_1_pir________________PPQKASKEHSWVDFAKKYLNMMDYLGTYFLPFYHSFTPIFQWYHPNINPYQRNEFKFQISFRVPVFRHILWTKGTLYLAYTQTDWFQIYNNPQSAPMRMINFMPELIYVYPINFKPFggNFSEIWIGWQHISNGVGGAqvSAGQRPVFRLVWEKGGLKIMVAYWPYVHYDQSNF
WP_021308106_1_pir____________PPQKASKEHSWADFAKKYLNMMDYLGTYFLPFYHSFTPIFQWYHPNINPYQRNEFKFQISFRVPVFRHILWTKGTLYLAYTQTNWFQIYNNPQSAPMRMINFMPELIYVYPINFEPFggKFSEIWIGWQHISNGVGGAqvSAGQRPVFRLVWEKGGLKIMVAYWPYVPYDQSNF
WP_021308106_1_pir____________PPQKASKEHSWADFAKKYLNMMDYLGTYFLPFYHSFTPIFQWYHPNINPYQRNEFKFQISFRVPVFRHILWTKGTLYLAYTQTNWFQIYNNPQSAPMRMINFMPELIYVYPINFEPFggKFSEIWIGWQHISNGVGGAqvSAGQRPVFRLVWEKGGLKIMVAYWPYVPYDQSNF
WP_000836092_1_pir____________PQKAAPKEHSWVDFAKKYLNMMDYLGTYFLPFYHSFTPIFQWYHPNINPYQRNEFKFQISFRVPVFRHILWTKGTLYLAYTQTNWFQIYNDPQSAPMRMINFMPELIYVYPINFKPFggNFSEIWIGWQHISNGVGGAqvSAGQRPVFRLVWEKGGLKIMVAYWPYIPYDQSNF
WP_000836092_1_pir____________PQKAAPKEHSWVDFAKKYLNMMDYLGTYFLPFYHSFTPIFQWYHPNINPYQRNEFKFQISFRVPVFRHILWTKGTLYLAYTQTNWFQIYNDPQSAPMRMINFMPELIYVYPINFKPFggNFSEIWIGWQHISNGVGGAqvSAGQRPVFRLVWEKGGLKIMVAYWPYIPYDQSNF
AFR51754_1_pir________________APQKAPKEHSWVDFAKKYLNMMDYLGTYFLPFYHSFTPIFQWYHPNINPYQRNEFKFQISFRVLVFRHILWTKGTLYLAYTQTDWFQIYNDPQSAPMRMMNFMPELIYVYPINFKPFggNFSEIWIGWQHISNGVGGAqvSAGQRPVFRLVWEKGGLKIMVAYWPYVPYDQSNF
WP_021305677_1_pir____________PPQKAPKEHSWVDFAKKYLNMMDYLGTYFLPFYHSFTPIFQWYHPNINPYQRNEFKFQISFRVPVFRHILWTKGTLYLAYTQTDWFQIYNNPQSAPMRMINFMPELIYVYPINFKPFggNFSEIWIGWQHISNGVGGAqvSAGQRPVFRLVWEKGGLKIMVAYWPYVPYDQSSF
WP_024422083_1_pir____________PPQKAPKEHSWADFAKKYLNMMDYLGTYFLPLYHSFTPIFQWYHPNINPYQRNEFKFQISFRVPVFRHILWTKGTLYLAYTQTNWFQIYNNPQSAPMRMINFMPELIYVYPINFEPFggKFSEIWIGWQHISNGVGGAqvSAGQRPVFRLVWEKGGLKIMVAYWPYVPYDQSNF
WP_000292766_1_pir____________PPQKAPKEHSWVDFAKKYLNMVDYLGTYFLPFYHSFTPIFQWYHPNINPYQRNEFKFQISFRVPVFRHILWTKGTLYLAYTQTNWFQIYNNPQSAPMRMMNFMPELIYVYPINFKPFggNFSEIWIGWQHISNGVGGAqvSAGQRPVFRLVWEKGGLKIMVAYWPYVPYVQSNF
WP_000292766_1_pir____________PPQKAPKEHSWVDFAKKYLNMVDYLGTYFLPFYHSFTPIFQWYHPNINPYQRNEFKFQISFRVPVFRHILWTKGTLYLAYTQTNWFQIYNNPQSAPMRMMNFMPELIYVYPINFKPFggNFSEIWIGWQHISNGVGGAqvSAGQRPVFRLVWEKGGLKIMVAYWPYVPYVQSNF
WP_000836119_1_pir____________PPQKAPKEHSWVDFAKKYLNMMDYLGTYFLPFYHSFTPIFQWYHPNINPYQRNEFKFQISFRVPVFRHILWTKGTLYLAYTQTNWFQIYNDPQSAPMRMINFMPELIYVYPINFEPFggKFSEIWIGWQHISNGVGGAqvSAGQRPVFRLVWEKGGLKIMVAYWPYVLYNQSNF
WP_052918420_1_pir____________PPQKAPKEYSWVDFAKKYLNMMDYLGTYFLPFYHSFTPIFQWYHPNINPYQRNEFKFQISFRVPVFRHILWTKGTLYLAYTQTDWFQIYNNPQSAPMRMINFMTELIYVYPINFKPFggNFSEIWIGWQHISNGVGGAqvSAGQRPVFRLVWEKGGLKIMVAYWPYVPYDQSNF
WP_000836125_1_pir____________PPQKAPKEHSWVDFAKKYLNMMDYLGTYFLPFYHSFTPIFQWYHPNINPYQRNEFKFQISFRVPVFRHILWTKGTLYLAYTQTNWFQIYNDPQSAPMRMINFMPELIYVYSINFEPFggKFSEIWIGWQHISNGVGGAqvSAGQRPVFRLVWEKGGLKIMVAYWPYVPYNQSNF
AFR51733_1_pir________________PPQKAQKDHSWVDFAKKYLNMMDYLGTYFLPFYHSFTPIFQWYHPNINPYQRNEFKFQISFRVPVFRHILWTKGTLYLAYTQTDWFQIYNNPQSAPMRMINFMPELIYVYPINFKPFggNFSEIWIGWQHISNGVGGAqvSAGQRPVFRLVWEKGGLKIMVAYWPYVPYDQSNF
WP_064433524_1_pir____________PPQKAPKEHSWVDFAKKYLNMMDYLGTYFMPFYHSFTPIYQWYHPNINPYQRNEFKFQISFRVPVFRHILWTKGTLYLAYTQTNWFQIYNNPQSAPMRMMNFMPELIYVYPINFKPFggNFSEIWIGWQHISNGVGGAevSAGQRPVFRLVWEKGGLKIMVAYWPYVPYDQSNF
WP_000836140_1_pir____________PPQKAPKEHSWVDFAKKYLNMMDYLGTYFLPFYHSFTPIFQWYHPNINPYQRNEFKFQISFRVPVFRHILWTKGTLYLAYTQTNWFQIYNNSQSAPMRMINFMPELIYVYPINFEPFggKFSEIWIGWQHISNGVGGAqvSAGQRPVFRLVWEKGGLKIMVAYWPYVPYDQSDF
WP_021308614_1_pir____________PPQKASKEHSWADFAKKYLNMMDYLGTYFLPFYHSFTPIFQWYHPNINPYQRNEFKFQISFRVPVFRHILWTKGTLYLAYTQTNWFQIYNNPQSAPMRMINFMPELIYVYPINFEPFggKFSEIWIGWQHISNGVGGVqvSAGQRPVFRLVWEKGGLKIMVAYWPYVPYDQSNF
WP_000836128_1_pir____________PPQKAPKEHSWVDFAKKYLNMMDYLGTYFLPFYHSFTPIFQWYHPNINPYQRNEFKFQISFRVPVFRHILWTKGTLYLAYTQTNWFQIYNNPQSAPMRMINFMPELIYIYPINFEPFggKFSEIWIGWQHISNGVGGAqvSAGQRPVFRLVWEKGGLKIMVAYWPYVPYDQSNF
AFR51765_1_pir________________PSQKAPKEHSWVDFAKKYLNMMDYLGTYFLPFYHSFTPIFQWYNPNINPYQRNEFKFQISFRVPVFRHILWTKGTFYLAYTQTNWFQIYNDPQSAPMRMMNFMPELIYVYPINFKPFggNFSEIWIGWQHISNGVGGAqvSAGQRPVFRLVWEKGGLKIMVAYWPYVPYDQSNF
WP_025313772_1_pir____________PPQKAPKEHSWVDFAKKYLNMMDYLGTYFLPFYHSFTPIFQWYHPNINPYQRNEFKFQISFRVPVFRHILWTKGMLYLAYTQTNWFQIYNDPQSAPMRMINFMPELIYVYPINFEPFggKFSEIWIGWQHISNGVGGAqvSAGQRPVFRLVWEKGGLKIMVAYWPYVPYNQSNF
WP_025313772_1_pir____________PPQKAPKEHSWVDFAKKYLNMMDYLGTYFLPFYHSFTPIFQWYHPNINPYQRNEFKFQISFRVPVFRHILWTKGMLYLAYTQTNWFQIYNDPQSAPMRMINFMPELIYVYPINFEPFggKFSEIWIGWQHISNGVGGAqvSAGQRPVFRLVWEKGGLKIMVAYWPYVPYNQSNF
WP_024949602_1_pir____________PPQKAPKEHSWADFAKKYLNMMDYLGTYFLPLYHRFTPIFQWYHPNINPYQRNEFKFQISFRVPVFRHILWTKGTLYLAYTQTNWFQIYNNPQSAPMRMINFMPELIYVYPINFEPFggKFSEIWIGWQHISNGVGGAqvSAGQRPVFRLVWEKGGLKIMVAYWPYVPYDQSNF
EKE89635_1_pir________________..........MDFAKKYLNMMDYLGTYFLPFYHSFTPIFQWYHPNINPYQRNEFKFQISFRVPVFRHILWTKGTLYLAYTQTNWFQIYNDPQSAPMRMMNFMPELIYVYPINFKPFggNFSEIWIGWQHISNGVGGAqvSTGQRPVFRLVWEKGGLKIMVAYWPYVPYDQSNF
EKE89635_1_pir________________..........MDFAKKYLNMMDYLGTYFLPFYHSFTPIFQWYHPNINPYQRNEFKFQISFRVPVFRHILWTKGTLYLAYTQTNWFQIYNDPQSAPMRMMNFMPELIYVYPINFKPFggNFSEIWIGWQHISNGVGGAqvSTGQRPVFRLVWEKGGLKIMVAYWPYVPYDQSNF
SFZ70766_1_pir________________APKKASKDHSWVDLAKKYLDVVDYQGTYFMPFYHSFTPVYQWYRPNINPYQRNEFKFQISFKMPVFKHFLWTKGTLYLAYTQTNWFQIYNDPQSAPMRMVNYMPELIYVYPINFEPFggNFSEIWAGWQHISNGVGGAevSAGQRPVFRLVWEKGGLKIMVAYWPYVPYDQSNF
WP_011577717_1_pir____________APKKASKDHSWVDLAKKYLDVVDYQGTYFMPFYHSFTPVYQWYRPNINPYQRNEFKFQISFKMPVFKRFLWTKGTLYLAYTQTNWFQIYNDPQSAPMRMVNYMPELIYVYPINFEPFggNFSEIWAGWQHISNGVGGAevSAGQRPVFRLVWEKGGLKIMVAYWPYVPYDQSNF
WP_011577717_1_pir____________APKKASKDHSWVDLAKKYLDVVDYQGTYFMPFYHSFTPVYQWYRPNINPYQRNEFKFQISFKMPVFKRFLWTKGTLYLAYTQTNWFQIYNDPQSAPMRMVNYMPELIYVYPINFEPFggNFSEIWAGWQHISNGVGGAevSAGQRPVFRLVWEKGGLKIMVAYWPYVPYDQSNF
WP_014658866_1_pir____________APKKAPKEHSWAKFAKKYLDIVDYQGTYFLPFYHSFTPIYEWYYPNLNEYQRNEFKFQISFKMPVFSHFLWTKGTLYLAYTQTNWFQIYNNPQSAPMRMVNYMPELIYVYPINFEPFggNFSEVWIGWQHISNGVGGAevSAGQRPVFRLVWQKWGLKVMVAYWPYVPYDQSNF
WP_014658866_1_pir____________APKKAPKEHSWAKFAKKYLDIVDYQGTYFLPFYHSFTPIYEWYYPNLNEYQRNEFKFQISFKMPVFSHFLWTKGTLYLAYTQTNWFQIYNNPQSAPMRMVNYMPELIYVYPINFEPFggNFSEVWIGWQHISNGVGGAevSAGQRPVFRLVWQKWGLKVMVAYWPYVPYDQSNF
WP_014660919_1_pir____________APKETPKEKSWARFAKRYLELIDYQGTYFLPIYHSFTPIYEWYHPNINPYQRNDFKFQISFKMPVFKHLLWTKGTLFLAYTQTNWFQIYNDPQSAPMRMVNYMPELIYVYPINFEPFggNFAELWIGWQHISNGVGGAavSAGQRPVFRLVWKKGGLKIMVAYWPYIPYNQSNF
WP_014660919_1_pir____________APKETPKEKSWARFAKRYLELIDYQGTYFLPIYHSFTPIYEWYHPNINPYQRNDFKFQISFKMPVFKHLLWTKGTLFLAYTQTNWFQIYNDPQSAPMRMVNYMPELIYVYPINFEPFggNFAELWIGWQHISNGVGGAavSAGQRPVFRLVWKKGGLKIMVAYWPYIPYNQSNF
EJB15462_1_pir________________PPQKAPKEHSWVDFAKKYLNMMDYLGTYFLPFYHSFTPIFQWYHPNINPYQRNEFKFQISFRVPVFRHILWTKGTLYLAYTQTDWFQIYNNPQSAPMRMINFMPELIYVYPINFEPFggKFSEIWIGWQHISNGVGGAqvSAGQRPVFRLVWEKGGLKIMVAYWPYVPYDQSNF
EJB15462_1_pir________________PPQKAPKEHSWVDFAKKYLNMMDYLGTYFLPFYHSFTPIFQWYHPNINPYQRNEFKFQISFRVPVFRHILWTKGTLYLAYTQTDWFQIYNNPQSAPMRMINFMPELIYVYPINFEPFggKFSEIWIGWQHISNGVGGAqvSAGQRPVFRLVWEKGGLKIMVAYWPYVPYDQSNF
WP_017345667_1_pir____________EVPGSIIANLLEKHDNPFVLYPYESNYVLYTYTSDMNKEAISSYNWADKAKKDEVKFQLSLAFPLWRGILGDNSVLAASYTQQSWWQLSNHKESSPFRETNYEPQLFLGWATDYSLGGWTLRDVEVGFNHQSNGRSDPTSRSWNRVYARLMAENGNFLAEIKPWYRIPEsdDNF
WP_003854378_1_pir____________QVQGSIIANLLEKHDNPFVLYPYENNYLLYTYTSDLNKEAISSYNWGENARKDEVKFQLSLAFPLWRGILGDNSVLAASYTQRSWWQLSNSAESSPFRETNYEPQIFLGWATDYSLAGWTLRDVEVGLNHQSNGRSDPTSRSWNRVYARLMAENGNFLAEIKPWYRIPEsDDNF
WP_050320734_1_pir____________AVRGSIIAAMLQDHDNPFLLYPYETNYLLYTYTNEINKQAISSYDWAEQANKDEVKFQLSLAFPIWRGIAGDNSLLGASYTQRSWWQASNSEESSPFRETNYEPQLFLAWSTDYELAGWTFREVEVEFGFNHQSNGKADPTSRSWNRAYTRVMAQRGNLEIDLKPWYRIPEkDDNF
WP_038628931_1_pir____________AVQGSIIANLLEKHDNPFVLYPYESNYLLYTLTSDMNKEAIESYDWADKAKKDEVKFQLSLAFPLWRGILGDNSVLAASYTQRSWWQLSNRGASSPFRETNYEPQIFLGWATDYSFAGWTLRDIELGLNHQSNGRSDPTSRSWNRVYARLMAENGNLLAEIKPWYRLpsNDDNF
WP_010257301_1_pir____________QVQGSIIANLLEKHDNPFVLYPYENNYLLYTYTSDLNKEAISSYNWGENARKDEVKFQLSLAFPLWRGILGDNSVLAASYTQRSWWQLSNSAESSPFRETNYEPQIFLGWATDYSLAGWTLRDVEVGLNHQSNGRSDPTSRSWNRVYARLMAENGNFLAEIKPWYRIPEsDDNF
WP_050109697_1_pir____________AVRGSIIAAMLQDHDNPFLLYPYETNYLLYTYTSDLNKDAISSYDWAENAKKDEVKFQLSLGFPIWRGIAGDNSLLGASYTQRSWWQASNSDESSPFRETNYEPQLFLAWATDYSLAGWTLRDVEVGLNHQSNGKADPTSRSWDRVYTRLMAQRGNLEIDLKPWYRLPEkDDNF
AHM76289_1_pir________________TVRGSIIAAMLQDHDNPFLLYPYETNYLLYTYTSDINKEAISAYDWAENAKKDEVKFQLSLGFPIWRGIAGDNSLLGASYTQRSWWQASNSDESSPFRETNYEPQLFLAWATDYELAGWTFREVEVEFGFNHQSNGKADPTSRSWDRVYTRLMAQRGNLEIDLKPWYRIPEkDDNF
WP_041143500_1_pir____________AVRGSIIANLLQDHDNPFLLYPYESNYLLYTWSSDINKEAISSYDWAENARKDEVKFQLSLAFPLWRGILGDNSVLAASYTQKSWWQLSNSKESAPFRETNYEPQLFLGFATDYRLAGWTLRDVEVGYNHDSNGRSDPTSRSWNRLYTRLMAQNGNWLVEVKPWYVIGSTDDNF
WP_012907912_1_pir____________AVQGSIIANMLVEHDNPFTLYPYDTNYLIYTNTSDLNKEAISTYNWSENARKDEVKFQLSLAFPLWRGILGPNSVLGASYTQKSWWQLSNSEESAPFRETNYEPQLFLGFATDYRFAGWTLRDIEMGYNHDSNGRSDPTSRSWNRLYTRLMAQNGNWLVEVKPWYVIGSTDDNF
WP_061061281_1_pir____________QVQGSIIANLLEKHDNPFVLYPYENNYLLYTYTSDLNKEAISSYNWGENARKDEVKFQLSLAFPLWRGILGDNSVLAASYTQRSWWQLSNSAESSPFRETNYEPQIFLGWATDYSLGGWTLRDVELGLNHQSNGRSDPTSRSWNRVYARLMAENGNFLAEIKPWYRIPEsDDNF
WP_002883408_1_pir____________AVRGSIIANLLQDHDNPFLLYPYESNYLLYTWTSDLNKEAIRSYDWAENARKDEVKFQLSLAFPLWRGILGDNSLLGASYTQKSWWQLSNSKESAPFRETNYEPQLFLGFATDYQFAGWTLRDIEMGYNHDSNGRSDPTSRSWNRLYARLMAQNGNWLVEVKPWYVVGNTDDNF
WP_023321873_1_pir____________AVRGSIIANLLQDHDNPFLLYPYESNYLLYTWTSDLNKEAIRSYDWAENARKDEVKFQLSLAFPLWRGILGENSLLGASYTQKSWWQLSNSKESAPFRETNYEPQLFLGFATDYSFAGWTLRDVEMGYNHDSNGRSDPTSRSWNRLYTRLMAQNGNWLVEVKPWYVIGSTDDNF
WP_062777415_1_pir____________AVRGSIIANLLQEHDNPFTLYPYESNYLLYTWTSDLNKEAIRTYNWAENARKDEVKFQLSLAFPLWRGILGDNSVLAASYTQKSWWQLSNSDESAPFRETNYEPQVFLGFATDYRFAGWTLRDVEMGYNHDSNGRSDPTSRSWNRLYARLMAQNGNWLVEVKPWYVLGETDDNF
WP_035894401_1_pir____________AVRGSIIANLLQEHDNPFTLYPYESNYLLYTWTSDLNKEAIRTYDWAENARKDEVKFQLSLAFPLWRGILGDNSVLAASYTQRSWWQMSNSDESAPFRETNYEPQLFLGFATDYRFAGWTLRDVEVGYNHDSNGRSDPTSRSWNRLYTRLMAQNGNWLVEVKPWYVIGDTDDNF
AJJ64943_1_pir________________AVRGSIIAAMLQDHDNPFLLYPYETNYLLYTYTSDINKNAISSYDWAQNAKKDEVKFQLSLGFPIWRGIAGDNSLLGASYTQRSWWQASNSDESSPFRETNYEPQVFLAWATDYELAGWTFREVEVEFGVNHQSNGKADPTSRSWNRVYTRLMAQRGNLEVDLKPWYRMPEkDDNF
WP_042547204_1_pir____________AVRGSIIAAMLQDHDNPFLLYPYETNYLLYTYTSDINKNAISSYDWAQNAKKDEVKFQLSLGFPIWRGIAGDNSLLGASYTQRSWWQASNSDESSPFRETNYEPQVFLAWATDYELAGWTFREVEVEFGVNHQSNGKADPTSRSWNRVYTRLMAQRGNLEVDLKPWYRMPEkDDNF
WP_013468740_1_pir____________APKKPKKSHPFLDFAKKYLDIMEYRGTYFMPFYHSFTPIYQWYHPDINRYQSTEFKFQISFKVPVFRHFLFTNGTLYLAYTQTNWFQIYNNPQSAPMRMVNYMPELIYVYPLNIPFfmGYLSEFWIGWQHISNGIGGRqvSAGQRPVFKLTWQKGGFKIRIGYWPYIPYNQSNF
WP_013468740_1_pir____________APKKPKKSHPFLDFAKKYLDIMEYRGTYFMPFYHSFTPIYQWYHPDINRYQSTEFKFQISFKVPVFRHFLFTNGTLYLAYTQTNWFQIYNNPQSAPMRMVNYMPELIYVYPLNIPFfmGYLSEFWIGWQHISNGIGGRqvSAGQRPVFKLTWQKGGFKIRIGYWPYIPYNQSNF
SFZ71270_1_pir________________APKKPKKSHPFLDFAKKYLDIMEYRGTYFMPFYHSFTPIYQWYHPDINRYQSTEFKFQISFKVPVFRHFLFTNGTLYLAYTQTNWFQIYNNPQSAPMRMVNYMPELIYVYPLNIPFfmGYLSEFWIGWQHISNGIGGRqvSAGQRPVFKLTWQKGGFKIRIGYWPYIPYNQSNF
SFZ71062_1_pir________________APTKPKKSHPFLDFAKKYLDIMEYRGTYFMPFYHSFTPIYQWYHPDINRYQSTEFKFQISFKVPVFRHFLFTNGTLYLAYTQTNWFQIYNNPQSAPMRMVNYMPELIYVYPLNIPFfmGYLSEFWIGWQHISNGIGGRqvSAGQRPVFKLTWQKGGFKIRIGYWPYIPYNQSNF
EEP90858_1_pir________________AVRGSIIAAMLQDHDNPFLLYPYETNYLLYTYTSDINKEAISSYDWAENAKKDEVKFQLSLGFPIWRGIAGDNSLLGASYTQRSWWQASNSDESSPFRETNYEPQIFLAWATDYEWAGWTFREVEVEFGYNHQSNGKADPTSRSWDRVYTRLMAQRGNLEIDLKPWYRIPEkDDNF
CFQ12651_1_pir________________TVRGSIIAAMLQDHDNPFLLYPYETNYLLYTYTSDINKDAISSYDWAQNANKDEVKFQLSLGFPIWRGIAGDNSLLGASYTQRSWWQASNKDESSPFRETNYEPQLFLAWATDYELAGWTFREVEVEFGYNHQSNGKADPTSRSWDRVYTRLMAQRGNLEIDLKPWYRLPEkDDNF
WP_067434574_1_pir____________AVRGSIIANLLEKHDNPFVLYPYEKNYLLYTYTSDMNREAISSYNWSDKVKKDEVKFQLSLAFPLWRGILGDNSVLAASYTQGSWWQLSNRGASSPFRETNYEPQIFLGWATDYSLGGWTLRDVETGFDHQSNGRSDPTSRSWNRLYARLMAQNGNFMVEVKPWYRLPedDDNF
WP_055769254_1_pir____________WELAKDSKLGLFQLRAYKPVYLLPAFWTDKPNRMPHSPNPANTVTEAEELDNTELKFQLSFKTKVAENLFGDNGDIWVGYTQSSHWQAYNSEISRPFRETNYEPEAMLVFRNGYSLFGWNGRMTGISLNHQSNGRSDPLSRSWNRVILNVGLDRDNWAIMLRPWYRIPedDNNF
WP_045378719_1_pir____________AVRGSIIANLLQEHDNPFLLYPYESNYLLYTWTSDLNKEAIRSYDWADDARKDEVKFQLSLAFPLWRGILGDNSLLGASYTQKSWWQLSNSKGSAPFRETNYEPQLFLGFATDYEFAGWTLRDIEVGYNHDSNGRSDPTSRSWNRLYTRLMAQNGNWLVEVKPWYVVGSTDDNF
WP_054668662_1_pir____________WELAKDSKLGLFQLRAYKPVYLLPAFWSSKPNRLPHSPNPANTATEAEDLDATELKFQLSFKTKVAENLFGDNGDIWVGYTQSSRWQAYNSEISRPFRETNYEPEAMLVFRNGYSLFGWNGRMTGISLNHQSNGRSDPLSRSWNRVILNVGLDRDNWAIMLRPWYRIPedDNNF
WP_005279368_1_pir____________AVRGSIIAAMLQDHDNPFLLYPYETNYLLYTYTSDINKDAISSYNWAENAKRDEVKFQLSLGFPIWRGILGDNSLLGASYTQRSWWQASNSDESSPFRETNYEPQLFLAWATDYELAGWTFREVEVEFGYNHQSNGKADPTSRSWDRVYTRVMAQRGNLEIDLKPWYRLPEkDDNF
CCV63406_1_pir________________TVRGSIIAAMLQDHDNPFLLYPYETNYLLYTYTSDINKDAIRSYDWAQNAKKDEVKFQLSLGFPIWRGIAGDNSLLGASYTQRSWWQASNSDESSPFRETNYEPQIFLAWATDYELAGWTFREVEVEFGYNHQSNGKADPTSRSWDRVYTRLMAQRGNLEIDLKPWYRIPEkDDNF
AAA67617_1_pir________________AVRGSIIANMLQEHDNPFTLYPYDTNYLIYTQTSDLNKEAIASYDWAENARKDEVKFQLSLAFPLWRGILGPNSVLGASYTQKSWWQLSNSEESSPFRETNYEPQLFLGFATDYRFAGWTLRDVEMGYNHDSNGRSDPTSRSWNRLYTRLMAENGNWLVEVKPWYVVGNTDDNF
1QD5_A_pir____________________AVRGSIIANMLQEHDNPFTLYPYDTNYLIYTQTSDLNKEAIASYDWAENARKDEVKFQLSLAFPLWRGILGPNSVLGASYTQKSWWQLSNSEESSPFRETNYEPQLFLGFATDYRFAGWTLRDVEMGYNHDSNGRSDPTSRSWNRLYTRLMAENGNWLVEVKPWYVVGNTDDNF
EHV01635_1_pir________________AVRGSIIANMLQEHDNPFTLYPYDTNYLIYTQTSDLNKEAIASYDWAENARKDEVKFQLSLAFPLWRGILGPNSVLGASYTQKSWWQLSNSEESSPFRETNYEPQLFLGFATDYRFAGWTLRDVEMGYNHDSNGRSDPTSRSWNRLYTRLMAENGNWLVEVKPWYVVGNTDDNF
ETJ18474_1_pir________________AVRGSIIANMLQEHDNPFTLYPYDTNYLIYTQTSDLNKEAIASYDWAENARKDEVKFQLSLAFPLWRGILGPNSVLGASYTQKSWWQLSNSEESSPFRETNYEPQLFLGFATDYRFAGWTLRDVEMGYNHDSNGRSDPTSRSWNRLYTRLMAENGNWLVEVKPWYVVGNTDDNF
1QD5template_pir______________AVRGSIIANMLQEHDNPFTLYPYDTNYLIYTQTSDLNKEAIASYDWAENARKDEVKFQLSLAFPLWRGILGPNSVLGASYTQKSWWQLSNSEESSPFRETNYEPQLFLGFATDYRFAGWTLRDVEMGYNHDSNGRSDPTSRSWNRLYTRLMAENGNWLVEVKPWYVVGNTDDNF
WP_064546457_1_pir____________AVRGSIIANLLQEHDNPFTLYPYESNYLLYTWTSDLNKEAIRSYDWAENARKDEVKFQLSLAFPLWRGILGDNSVLAASYTQRSWWQLSNSGESAPFRETNYEPQMFLGFATDYRFAGWTLRDVEVGFNHDSNGRSDPTSRSWNRLYTRLMAQNGNWLVEVKPWYVIGDTDDNF
WP_049562453_1_pir____________AVRGSIIAAMLQDHDNPFLLYPYETNYLLYTYTSDINKEAISSYDWAENAKKDEVKFQLSLGFPIWRGIAGDNSLLGASYTQRSWWQASNSDESSPFRETNYEPQLFLAWATDYELAGWTFREVEFGYNHQSNGKADPTSRSWDRVYTRLMAQRGDLEIDLKPWYRLPEkDDNF
WP_003017916_1_pir____________AVRGSIIANMLQEHDNPFTLYPYDTNYLIYTNTSDMNKEAISSYSWSENARKDEVKFQLSLAFPLWRGILGPNSVLGGSYTQKSWWQLSNSEESSPFRETNYEPQLFLGFATDYRFAGWTLRDVEMGYNHDSNGRSDPTSRSWNRLYTRLMAENGNWLVEVKPWYVIGSTDDNF
WP_047066634_1_pir____________AVRGSIIANLLQDHDNPFLLYPYESNYLLYTWTSDLNKEAIRSYDWADDARKDEVKFQLSLAFPLWRGILGDNSLLGASYTQKSWWQLSNSKGSAPFRETNYEPQLFLGFATDYEFAGWTLRDIEVGYNHDSNGRSDPTSRSWNRLYTRLMAQNGNWLVEVKPWYVVGSTDDNF
WP_032718480_1_pir____________AVRGSIIANLLQDHDNPFLLYPYESNYLLYTWSSDINKEAISSYDWAENARKDEVKFQLSLAFPLWRGILGENSLLGASYTQKSWWQLSNSKESAPFRETNYEPQLFLGFATDYQLAGWTLRDVEMGYNHDSNGRSDPTSRSWNRLYARLMAQNGNWLVEVKPWYVVGSTNDNF
```

WP_004772421_1_pir____________ WELSEDSKLGTWNIRAYQPVYLLPVFWTSDKNEFPSSPNPENTVKDKQELTSSEAKFQISFKTKAWENIFGDNGDLWLGYTQSSRWQVYNSDESRPFRETNYEPEASLMFRTNYEVLGLNARLLGVTLNHQSNGRSDPLSRSWNRVIFNLGFEKDNFALMLRPWYRVEedDNNF

WP_033754601_1_pir____________ QVRGSIIANLLEKHDNPFVLYPYESNYLLYTYTSDINKEAISSYNWADNARKDEVKFQLSLAFPLWRGILGDNSVLAASYTQRSWWQLSNRGESSPFRETNYEPQIFLGWATDYDFAGWTLRDVEVGFNHQSNGRSDPTSRSWNRVYARLMAQNGNFLAEVKPWYRIPDsdDNF

WP_052283953_1_pir____________ AVRGSILANLLQEHDNPFTLYPYESNYLLYTWTSDLNKEAIRSYDWAENARKDEVKFQLSLAFPLWRGILGDNSVLAASYTQRSWWQLSNSGESAPFRETNYEPQLFLGFATDYQFAGWTLRDIEMGYNHDSNGRSDPTSRSWNRLYTRLMAQNGNWLVEVKPWYVVGETNDNF

EIQ60709_1_pir________________ AVRGSIIANMLQEHDNPFTLYPYDTNYLIYTQTSDLNKEAIASYDWAENARKDEVKFQLSLAFPLWRGILGPNSVLGASYTQKSWWQLSNSEESSPFRETNYEPQLFLGFATDYRFAGWTLRDVEMGYNHDSNGRSDPTSRSWNRLYTRLMAENGNWLVEVKPWYVVGNTDDNF

WP_075183429_1_pir____________ AVRGSIIANMLEKHDNPFVLYPYENNYLLYTYTTNLNKDAIESYDWSNDAKKDEVKFQLSLAFPLWRGILGDNSVLGASYTQRSWWQLSNRGASSPFRETDYEPQVFLGWATDYQLAGWTLRDVEVGLNHQSNGRADPTSRSWNRVYARLMAQNGNFLAEVKPWYRIPEsdDNF

WP_001259597_1_pir____________ AVRGSIIANMLQEHDNPFTLYPYDTNYIIYTQTSDLNKEAIASYDWAENARKDEVKFQLSLAFPLWRGILGPNSVLGASYTQKSWWQLSNSEESSPFRETNYEPQLFLGFATDYRFAGWTLRDVEMGYNHDSNGRSDPTSRSWNRLYTRLMAENGNWLVEVKPWYVVGNTDDNF

WP_050115202_1_pir____________ AVRGSIIAAMLQDHDNPFLLYPYETNYLLYTYTSDINKNAISSYDWAEHANKDEVKFQLSLGFPIWRGIAGDNSLLGASYTQRSWWQASNKDESSPFRETNYEPQVFLAWATDYELAGWTFREVEFGYNHQSNGKADPTSRSWDRVYTRLMAQRGNLEIDLKPWYRLPekDDNF

WP_042292764_1_pir____________ SVQGSIIANMLVEHDNPFTLYPYDTNYLIYTSTSDLNKEAISTYNWSENARKDEVKFQLSLAFPLWRGILGPHSVLGASYTQKSWWQLSNSEESSPFRETNYEPQLFLGFATDYRFAGWTLRDVEMGYNHDSNGRSDPTSRSWNRLYTRLMAENGNWLVEVKPWYVIGSTDDNF

WP_005230732_1_pir____________ WELSEDSKLGTWNIRAYQPVYLLPVFWTSDKNEFPSSPNPENTVKDKQELTSSEAKFQISFKTKAWENIFGDNGDLWLGYTQSSRWQVYNSDESRPFRETNYEPEASLMFRTNYEILGLNARLLGVTLNHQSNGRSDPLSRSWNRVIFNLGFEKDNFALMLRPWYRVEedDNNF

WP_052900570_1_pir____________ AVRGSIIANLLEKHDNPFVLYPYENNYLLYTATSDLNKEAIQSYDWADKAKKDEVKFQLSLAFPLWSGILGDNSVLAASYTQRSWWQLSNRGASSPFRETNYEPQIFLGWATDYRFAGWTLRDVELGLNHQSNGRSDPTSRSWNRVYARLMAQNGNFMAEVKPWYRLPEsdDNF

WP_017800665_1_pir____________ AVRGSIIANLLEKHDNPFVLYPYENNYLLYTYTSNLNTEAIESYDWADKAKKDEVKFQLSLAFPLWRGIFGDNSLLAASYTQRSWWQLSNRGASSPFRETDYEPQIFLGWATDYNLAGWTLRDVEFGLNHQSNGRSDPTSRSWNRVYARLMAQNGNFMAEVKPWYRLPEsdDNF

WP_005275171_1_pir____________ WELSEESKLGVWNIRAYQPVYLLPVFWTSDKNEFPSSPNPENTVKEKQALTSSEAKFQLSFKTKAWENIFGDNGDLWLGYTQSSRWQVYNSEESRPFRETNYEPEASLMFRTNYEILGLNARLLGVTLNHQSNGRSDPLSRSWNRVIFNLGFEKDNFALMVRPWYRIEedDNNF

WP_038638216_1_pir____________ TVRGSIIAAMLQDHDNPFLLYPYETNYLLYTYTSDINKDAISSYDWAQNANKDEVKFQLSLGFPIWRGIAGDNSLLGASYTQRSWWQASNKDESSPFRETNYEPQVFLAWATDYELAGWTFREVEFGYNHQSNGKADPTSRSWDRVYTRFMAQRGNLEIDLKPWYRLPekDDNF

KLD76872_1_pir________________ WELAKDSKLGNFQLRAYKPVYLLPAFWTSKKNQTPSSPNPNNTVTSAEPLDSSEAKFQLSFKTKIVENIFGDNGDLWGAYTQSSRWQVYNSEQSRPFRETNYEPELALVFRNNYSLFGWKGRMTGIQLNHQSNGRSDPFSRSWNRAILNIGLDRENWALVLRPWYRIPEsdNNF

WP_069577939_1_pir____________ WELSEDSKLGTWNIRAYQPVYLLPVFWTSDKNEFPSSPNPENTVKDKQELTSSEAKFQISFKTKAWENIFGDNGDLWLGYTQSSRWQVYNSDESRPFRETNYEPEASLMFRTNYEVLGLNARLLGVTLNHQSNGRSDPLSRSWNRVIFNLGFEKDNFALMLRPWYRVEedDNNF

WP_023653454_1_pir____________ AVPGSIIANLLEKHDNPFVMYPYESNYLLYTDTSNLNKEAIRTYSWSDKAKHDEVKFQLSLAFPLWRGIVGDNSVLAASYTQKSWWQLSNRSESSPFRETDYEPQIFLGWATDYRFAGWTLRDVETGFNHQSNGRSEPTSRSWNRVYARLMAENGNWLVEVKPWYRLPEsdDNF

WP_049847184_1_pir____________ AVRGSIIANLLQEHDNPFTLYPYESNYLLYTWTSDLNKEAIRSYSWSENARKDEVKFQLSLAFPLWRGILGDNSVLGASYTQRSWWQLSNSKESSPFRETNYEPQLFLGFATDYSLAGWTLRDVEFGYNHDSNGRSDPTSRSWNRLYTRLMAQNGNWLVEVKPWYVVGSTDDNF

SFZ72838_1_pir________________ APAKPKKTHPFVEFARKYLELMEYRGTYFMPYYHSFTPIYQWYHPDINRYQSTEFKFQISFRVPVFRHFLFTTGTLYLAYTQTNWFQIYNNPQSAPMRMVNYMPELIYVYPLNLSikLGTLSEFWFGGQHISNGIGGRqvSAGQRPVFHLVWQKGGLKVNVAYWPYIPYNQSNF

SFZ72838_1_pir________________ APAKPKKTHPFVEFARKYLELMEYRGTYFMPYYHSFTPIYQWYHPDINRYQSTEFKFQISFRVPVFRHFLFTTGTLYLAYTQTNWFQIYNNPQSAPMRMVNYMPELIYVYPLNLSikLGTLSEFWFGGQHISNGIGGRqvSAGQRPVFHLVWQKGGLKVNVAYWPYIPYNQSNF

WP_001201668_1_pir____________ AVRGSIIANMLQEHDNPFTLYPYDTNYLIYTYTSDLNKEAIRTYNWSENARKDEVKFQLSLAFPLWRGILGDNSVLGASYTQKSWWQLSNSKESAPFRETNYEPQLFLGFATDYRFAGWTLRDVEMGYNHDSNGRSDPTSRSWNRLYTRLMAENGNWLVEVKPWYVIGSTDDNF

AJJ17614_1_pir________________ EVRGSIIAAMLQDHDNPFLLYPYETNYLLYTYTSNINKDAISSYDWANNARKDEVKFQLSLGFPIWRGIAGDNSLLGASYTQRSWWQASNSGESSPFRETNYEPQVFLAWATDYELAGWNFREVEVGLNHQSNGKADPTSRSWNRAYTRVMAQRGNLEIDLKPWYRLPekDDNF

WP_050297678_1_pir____________ EVRGSIIAAMLQDHDNPFLLYPYETNYLLYTYTSNINKDAISSYDWANNARKDEVKFQLSLGFPIWRGIAGDNSLLGASYTQRSWWQASNSGESSPFRETNYEPQVFLAWATDYELAGWNFREVEVGLNHQSNGKADPTSRSWNRAYTRVMAQRGNLEIDLKPWYRLPekDDNF

WP_026143478_1_pir____________ WELAKDSKLGTFQLRAYKPVYLLPAFWTSKKNELPSSPNPVNTVTTAEPLDSVEAKFQLSFKTKIVENIFGDNGDLWGAYTQSSRWQVYNAEQSRPFRETNYEPELMLVFRNNYSLFGWKGRMTGIQLTHQSNGRSDPLSRSWNRAMLNIGLDRDNWALVLRPWYRIPEsdNNF

WP_061706552_1_pir____________ AVRGSVIANLLQEHDNPFTLYPYDTNYVIYTDTSDLNKEAISSYSWSDNARKDEVKFQLSLAFPFWRGILGPNSVLGASYTQKSWWQLSNSGESAPFRETNYEPQLFLGFATDYEFAGWTLRDVEVGYNHDSNGRSDPTSRSWNRVYTRLMAENGNWLVEVKPWYVVGSTDDNF

WP_004662296_1_pir____________ WELSEESKLGVWNIRAYQPVYLLPAFWTSDKNEFPSSPNPNNTVKDQQNLTSTEAKFQISLKTKALENILGDNGDLWLGYTQSSHWQVYNSEESRPFRETNYEPEASLMFRTNYEILGLNARLLGVTLNHQSNGRSDPLSRSWNRVIFNLGFEKDNFALMLRPWYRVEedDNNF

WP_021506965_1_pir____________ AVKGSIIANLLEKHDNPFVLYPYENNYLLYTYTSDMNKEAISSYNWADKAKKDEVKFQLSLAFPLWRGILGDNSVLAASYTQRSWWQLSNRGASSPFRETNYEPQIFLGWATDYSLGGWTLRDVELGLNHQSNGRSDPTSRSWNRAYARLMAENGNFLAEIKPWYRLPEsdDNF

WP_005294551_1_pir____________ WELSEESKLGVWNIRAYQPVYLLPMFWTSDKNEFPSSPNPENTVKDKQDLKSSEAKFQLSFKTKAWENIFGDNGDLWVGYTQSSRWQVYNSEESRPFRETNYEPEASLMFRTNYEVLGLNARLLGVTLNHQSNGRSDPLSRSWNRVIFNLGFEKDNFALMVRPWYRVEedDNNF

WP_001259600_1_pir____________ VVRGSIIANMLQEHDNPFTLYPYDTNYIIYTQTSDLNKEAIASYDWAENARKDEVKFQLSLAFPLWRGILGANSVLGASYTQKSWWQLSNSEESSPFRETNYEPQLFLGFATDYRFAGWTLRDVEMGYNHDSNGRSDPTSRSWNRIYTRLMAENGNWLVEVKPWYVVGNTDDNF

WP_050087000_1_pir____________ EVRGSIIAAMLQDHDNPFLLYPYETNYLLYTYTSNINKDAISSYDWANNARKDEVKFQLSLGFPIWRGIAGDNSLLGASYTQRSWWQASNSGESSPFRETNYEPQVFLAWATDYELAGWNFREVEVGLNHQSNGKADPTSRSWNRAYTRIMAQRGNLEIDLKPWYRLpeKDDNF

SFZ73147_1_pir________________ APAKPKKTHPFVEFARKYLELMEYRGTYFMPYYHSFTPIYQWYHPDINRYQSTEFKFQISFRVPVFRHFLFTTGTLYLAYTQTNWFQIYNNPQSAPMRMVNYMPELIYVYPLNLSikLGTLSEFWFGGQHISNGIGGRqvSAGQRPVFHLVWQKGGFKINVAYWPYIPYNQSNF

CNI23333_1_pir________________ AVRGSIIAAMLQDHDNPFLLYPYETNYLLYTYTSDINKDAISSYDWAQNAKRDEVKFQLSLGFPIWRGIAGENSLLGASYTQRSWWQASNSDESSPFRETNYEPQLFLAWATDYELAGWTFREVEFGYNHQSNGKADPTSRSWDRVYTRVMAQRGNLEIDLKPWYRLPekDDNF

WP_038160466_1_pir____________ AVRGSIIANLLQEHDNPFTLYPYESNYLLYTWTSDLNKEAIRSYDWSENARKDEVKFQLSLAFPLWRGILGDNSVLGASYTQRSWWQLSNSKESAPFRETNYEPQLFLGFATDYSFAGWTLRDVEFGYNHDSNGRSDPTSRSWNRLYTRLMAQNGNWLVEVKPWYVIGSTNDNF

WP_001259598_1_pir____________ AVRGSVIANMLQEHDNPFTLYPYDTNYIIYTDTSDLNKEAISSYNWSENARKDEVKFQLSLAFPLWRGILGPNSVLGASYTQKSWWQLSNSKESSPFRETNYEPQLFLGFATDYRFAGWTLRDVEMGYNHDSNGRSDPTSRSWNRIYTRLMAENGNWLVEVKPWYVVGNTDDNF

EGC97070_1_pir________________ AVRGSVIANMLQEHDNPFTLYPYDTNYIIYTDTSDLNKEAISSYNWSENARKDEVKFQLSLAFPLWRGILGPNSVLGASYTQKSWWQLSNSKESSPFRETNYEPQLFLGFATDYRFAGWTLRDVEMGYNHDSNGRSDPTSRSWNRIYTRLMAENGNWLVEVKPWYVVGNTDDNF

WP_009088467_1_pir____________ QVQGSIIANLLEKHDNPFVLYPYENNYLLYTYTSDLNKEAISSYNWGENARKDEVKFQLSLAFPLWRGILGDNSVLAASYTQRSWWQLSNRGESSPFRETNYEPQIFLGWATDYSLGGWTLRDVELGLNHESNGRSDPTSRSWNRVYARLMAENGNFLAEIKPWYRIPDgdDNF

WP_031375034_1_pir____________ QVRGSIIANLLEKHDNPFVLYPYENNYLLYTYTSDLNKEAISSYNWAENARKDEVKFQLSLAFPLWRGILGDNSVLAASYTQRSWWQLSNRGESSPFRETNYEPQIFLGWATDYSFAGWTLRDVEVGLNHESNGRSDPTSRSWNRVYTRLMAENGNFLAEIKPWYRIPesDDNF

WP_023185187_1_pir____________ AVRGSIIANMLQEHDNPFTLYPYDTNYLIYTNTSDLNKEAIRTYNWSENARKDEVKFQLSLAFPLWRGILGPNSVLGASYTQKSWWQLSNSKESSPFRETNYEPQLFLGFATDYRFAGWTLRDVEMGYNHDSNGRSDPTSRSWNRLYTRLMAENGNWLVEVKPWYVVGSTDDNF

WP_005188153_1_pir____________ WELSEESKLGVWNIRAYQPVYLLPVFWTSDKNEFPSSPNPENTVKEKQELTSSEAKFQLSFKTKAWENIFGDNGDLWLGYTQSSRWQVYNSEESRPFRETNYEPEASLMFRTNYEVLGLNARLLGVTLNHQSNGRSDPLSRSWNRVIFNLGFEKDNFALMVRPWYRVEedDNNF

WP_051412633_1_pir____________ WELAKDSKLGIFQLRAYKPVYALPAFWTSKPNTRPHSPNPNNTVTTPEELDSVEAKFQISFKTKLAEDLFGDNGDIWAGYTQSSRWQAYNSEISRPFRETNYEPEVMLVFRNNYSLFGWKGRMMGVGINHQSNGRSDPLSRSWNRVIFTAGLDRENWAFVVRPWYRVSedDNNF

WP_006535791_1_pir____________ LVQGSIISGLLQEYDSPFVLYPYESNYIIYTDTSDMNKEAIQSYDWGNKAKKDEVKFQLSLAFPLWRGIAGENSVLAASYTQRSWWQLSNKKESAPFRETNYEPQLFLGWATDYKFAGWTLREIETGFNHESNGRSDPTSRSWNRAYARFMAQKGNLQLDLKPWYRFSEsdDNF

WP_001259602_1_pir____________ AVRGSIIANMLLEHDNPFTLYPYDTNYVIYTQTSDMNKEAIASYDWSKNARKDEVKFQLSLAFPLWRGILGPNSVLGASYTQKSWWQLSNSEESSPFRETNYEPQLFLGFATDYRFAGWTLRDVEMGYNHDSNGRSDPTSRSWNRLYTRLMAENGNWLLEVKPWYVIGNTDDNF

WP_036939519_1_pir____________ LVQGSIISGLLQEYDSPFVLYPYESNYIIYTDTSDMNKEAIESYDWGNKARKDEVKFQLSLAFPLWRGIAGENSVLAASYTQRSWWQLSNKKESAPFRETNYEPQLFLGWATDYKFAGWTLREIETGFNHESNGRSDPTSRSWNRVYARFMAQKGNLQLDLKPWYRFSEsdDNF

WP_056234787_1_pir____________ DVPGSIIANLLEKHDNPFVLYPYENNYLLYTWTSDINKDAIASYNWADKAKHDEVKFQLSLAFPLWRGILGDNSVLAASYTQKSFWQLSNRGASSPFRETDYEPQIFLGWSTDYSLGGWTLRDVETGFNHQSNGRSEPTSRSWNRVYARLMAENGNWLVEVKPWYRLPEsdDNF

WP_013507363_1_pir____________ AVRGSIIANLLEKHDNPFVLYPYENNYLLYTYTSDMNKEAISSYDWADKAKKDEVKFQLSLAFPLWRGILGDNSVLAASYTQRSWWQLSNRGASSPFRETNYEPQIFLGWATDYSLGGWTLRDVEMGLNHESNGRSDPTSRSWNRVYARLMAENGNFLAEIKPWYRIPEsdDNF

WP_005241783_1_pir____________ WELSEESKLGVWNIRAYQPVYLLPVFWTSDKNEFPSSPNPENTVKQKQDLTSSEAKFQLSFKTKAWENIFGDNGDLWLGYTQSSRWQVYNSKESRPFRETNYEPEASLMFRTNYEILGLNARLLGVTLNHQSNGRSDPLSRSWNRVIFNLGFEKDNFALMVRPWYRIEedDNNF

WP_038343952_1_pir____________ WELSEDSKLGVWNIRAYQPVYLLPVFWTSDKNEFPSSPNPENTVKEKQDLTSSEAKFQISFKTKAWENIFGNNGDLWLGYTQSSRWQVYNSAESRPFRETNYEPEASLMFRTNYEILGLNARLLGVTLNHQSNGRSDPLSRSWNRVIFNLGFEKDNFALMLRPWYRVEedDNNF

WP_004154940_1_pir____________ AVPGSIIANLLEKHDNPFVMYPYESNYLLYTDTSNLNKEAIQSYSWSDKAKHDEVKFQLSLAFPLWRGIVGDNSVLAASYTQKSWWQLSNRGASSPFRETDYEPQIFLGWATDYRFAGWTLRDVETGFNHQSNGRSEPTSRSWNRVYARLMAENGHWLVEVKPWYRIPEsdDNF

WP_049608949_1_pir____________ EVRGSIIAAMLQDHDNPFLLYPYETNYLLYTYTSDINKEAISSYDWAEHAKKDEVKFQLSLGFPIWRGILGDNSLLGASYTQRSWWQASNSDESSPFRETNYEPQLFLAWATDYEVAGWTFREVEFGYNHQSNGKADPTSRSWDRIYTRVMAQRGNLEIDLKPWYRLpeKDDNF

WP_073708272_1_pir____________ AVRGSIIANMLQEHDNPFTLYPYDTNYLIYTNTSDLNKEAISTYNWSENARKDEVKFQLSLAFPLWRGILGPNSVLGASYTQKSWWQLSNSKESSPFRETNYEPQLFLGFATDYRFAGWTLRDVEMGYNHDSNGRSDPTSRSWNRLYTRLMAENGNWLVEVKPWYVIGSTDDNF

EHJ80011_1_pir________________ AVRGSIIANMLQEHDNPFTLYPYDTNYLIYTNTSDLNKEAISTYNWSENARKDEVKFQLSLAFPLWRGILGPNSVLGASYTQKSWWQLSNSKESspFRETNYEPQLFLGFATDYRFAGWTLRDVEMGYNHDSNGRSDPTSRSWNRLYTRLMAENGNWLVEVKPWYVIGSTDDNF

WP_074006710_1_pir____________ AVRGSIIANMLQEHDNPFTLYPYDTNYLIYTNTSDLNKEAISTYNWSENARKDEVKFQLSLAFPLWRGILGPNSVLGASYTQKSWWQLSNSKESSPFRETNYEPQLFLGFATDYRFAGWTLRDVEMGYNHDSNGRSDPTSRSWNRLYTRLMAENGNWLVEVKPWYVIGSTDDNF

WP_015966424_1_pir____________ AVKGSIMANLLQEHDNPFTLYPYESNYLLYTWTSDLNKEAISSYDWAEHARKDEVKFQLSLAFPLWRGILGDNSVLGASYTQRSWWQLSNSGESSPFRETNYEPQLFLGFATDYSWEGWTLRDVEFGFNHDSNGRSDPTSRSWNRLYTRLMAQNGNWLVEVKPWYVVGNSNDNF

WP_054882784_1_pir____________ AARGSIIAAMLEDHDNPFLLYPYETNYLLYTYTSDINKDAISSYDWAENAKKDEVKFQLSLGFPIWRGIAGENSLLGASYTQRSWWQASNSDESAPFRETNYEPQMFLAWATDYEFAGWTFREVEFGFNHQSNGKADPTSRSWNRVYTRVMAQRGNLEVDLKPWFRldEKDDNF

WP_004639251_1_pir____________ WELSEESKLGAWNIRAYQPVYLLPVFWTSNKNEFPSSPNPVNTVDEKQNLTSSEAKFQLSLKTKAWENIFGNNGDLWLGYTQSSRWQVYNSEESRPFRETNYEPEASLMFRTNYEILGLNARLLGVTLNHQSNGRSDPLSRSWNRVIFNLGFEKDNFALMLRPWYRVEedDNNF

WP_057631914_1_pir____________ WELAKDSKLGLFQLRAYKPVYLLPAFWSSKPNVMPHSPNPNNSVTAPEELDGTELKFQLSFKTKVTENLFGDNGDIWVGYTQSSRWQAYNSELSRPFRETNYEPEVMLTFRNGYSLFGWNGRMTGISLNHQSNGRSDPLSRSWNRVILNVGLDRENWAIMLRPWYRIPEsdNNF

WP_024966089_1_pir____________ AVRGSIIANLLEKHDNPFVLFPYESNYLLYSVTSNINQSALESYSWADNVKRDEVKFQLSLAFPLWRGILGDNSVLAASYTQGSWWQLSNRAESSPFRETNYEPQVFLGWATDYSFAGWTLRDVEVGLNHQSNGRSDPTSRSWNRVYARLMAQNGNWMVEAKPWFRIAEsdDNF

WP_039381766_1_pir____________ AVKGSVIANLLEKHDNPFVLYPYENNYLLYTYTSDMNKEAISSYNWADKAKKDEVKFQLSLAFPLWRGILGDNSVLAASYTQRSWWQLSNRGASSPFRETNYEPQLFLGWATDYSLAGWTLRDVELGVNHQSNGRSDPTSRSWNRLYARLMAENGNFLAEIKPWYRLPEsdDNF

ADD75343_1_pir________________ QVRGSIIANLLEQHDNPFVLYPYENNYLLYTYTSDINKEAISSYNWAQNARKDEVKFQLSLAFPLWRGILGDNSVLAASYTQRSWWQLSNKKESSPFRETNYEPQIFLGWATDYSFAGWTLRDVEVGVNHQSNGRSDPTSRSWNRVYTRLMAQNGNFLAEIKPWFRISetDDNF

WP_062748621_1_pir____________ AVRGSIIANLLEKHDNPFILFPYENNYLLYTDTSNINKDAIESYDWGNKAKHDEVKFQLSLAFPLWRGILGDNSVLAASYTQRSWWQLSNRGASSPFRETNYEPQIFLGWATDYSFAGWTLRDVETGLNHQSNGRSDPTSRSWNRVYARLMAQNGNWMVEAKPWFRLPEsdDNF

WP_063877908_1_pir____________ QVRGSIIANLLEQHDNPFVLYPYENNYLLYTYTSDINKEAISSYNWAKNARKDEVKFQLSLAFPLWRGIVGDNSVLAASYTQRSWWQVSNKKESSPFRETNYEPQIFLGWATDYSLAGWTLRDVEVGVNHQSNGRSDPTSRSWNRVYTRLMAQNGNFLVEIKPWFRISetDDNF

WP_005054159_1_pir____________ WELSEESKLGVWNIRGYQPVYLLPVFWTSDKNEFPSSPNPQNTVTDKQELTSSEAKFQLSLKTKALENIFGNNGDLWLGYTQSSRWQVYNSEESRPFRETNYEPEVSLMFRTNYEILGLNARLLGVTLNHQSNGRSDPLSRSWNRVIFNLGFEKNNFALMLRPWYRVEedDNNF

WP_004879781_1_pir____________ WELSEESKLGVWNIRAYQPVYLLPAFWTSDKNEFPSSPNPNNTVTDKQNLTSTEAKFQISLKTKAWENIIGNNGDLWLGYTQSSRWQVFNSEESRPFRETNYEPEASLMFRTNYEVLGLNARLLGVTLNHQSNGRSDPLSRSWNRVIFNLGFEKDNFALMLRPWYRVEedDNNF

EHC30066_1_pir________________ AVRGSIIANMLQEHDNPFTLYPYDTNYLIYTNTSDLNKEAISTYNWSENARKDEVKFQLSLAFPLWRGILGPNSVLGASYTQKSWWQLSNSKEsaAFRETNYEPQLFLGFATDYRFAGWTLRDVEMGYNHDSNGRSDPTSRSWNRLYTRLMAENGNWLVEVKPWYVIGSTDDNF

WP_004246319_1_pir____________ LVQGSIISGLLQEYDSPFVLYPYESNYIIYTKTSDMNKEAIASYDWGHKAKKDEVKFQISLAFPLWRGIAGENSVLAASYTQRSWWQLSNKKESAPFRETNYEPQLFLGWATDYQFAGWTLREIETGFNHESNGRAEPTSRSWNRVYARFMAQKGNFQLDLKPWYRFNEsdDNF

KXC00751_1_pir________________ LVQGSIISGLLQEYDSPFVLYPYESNYIIYTKTSDMNKEAIASYDWGHKAKKDEVKFQISLAFPLWRGIAGENSVLAASYTQRSWWQLSNKKESAPFRETNYEPQLFLGWATDYQFAGWTLREIETGFNHESNGRAEPTSRSWNRVYARFMAQKGNFQLDLKPWYRFNEsdDNF

WP_005160038_1_pir____________ WELSEESKLGVWNIRAYQPVYLLPVFWTSDKNEFPSSPNPQNTVKDKQELTSSEAKFQISFKTKAWENILGNNGDLWVGYTQSSRWQVYNSEESRPFRETNYEPEASLIFRTNYEILGLNARLLGVTLNHQSNGRSDPLSRSWNRVIFNLGFEKDNFALMLRPWYRIEedDNNF

SFN50945_1_pir________________ VVKGSIIANLLQNHDNPFLLYPYESNYVLYTYTSNMNKDAIDSYNWSDKAKKDEVKFQLSLAFPLWRGVLGDNSVLAASYTQRSWWQFSNRSASSPFRETDYEPQIFLGWATDYSVAGWTLRDVEVGLNHQSNGRSEPTSRSWNRVYARLMAQNGNWLAEVKPWYRVPEsdDNF

WP_006565291_1_pir____________ APRRPKKHNILIEFAKKYLDIMEYRGTYFLPFYYSFTPIYQWYHPDINRYQPVDFKFQISFKVPVIHHFLYTKGTLYLAYTETNWFQIYNNPQSAPMRMVNYMPELIYIYPLKIPLfwGTFSEFWIGWQHISNGIGGRqvSAGQRPVFHLVWQKGGLKINVAYWPYIPYNQSNF

WP_006565291_1_pir____________ APRRPKKHNILIEFAKKYLDIMEYRGTYFLPFYYSFTPIYQWYHPDINRYQPVDFKFQISFKVPVIHHFLYTKGTLYLAYTETNWFQIYNNPQSAPMRMVNYMPELIYIYPLKIPLfwGTFSEFWIGWQHISNGIGGRqvSAGQRPVFHLVWQKGGLKINVAYWPYIPYNQSNF

WP_004677305_1_pir____________ WELSEESKLGVWNIRAYQPVYLLPAFWTSDKNEFPSSPNPQNTVNEKQELTSSEAKFQISFKTKAWENILGNNGDLWIGYTQSSRWQVYNSEESRPFRETNYEPEASLMFRTNYEILGLNARLLGVTLNHQSNGRSDPLSRSWNRVIFNLGFEKDNFALMLRPWYRVEedDNNF

WP_039031205_1_pir____________ AVRGSIIANLLEEHDNPFTLYPYDSNYLIYTATSDLNKEAIKTYDWADDSHKDEVKFQLSLAFPLWRGILGPNSILGGSYTQKSWWQLSNSDESSPFRETNYEPQLFLGFATDYSLAGWTLRDVEFGYNHQSNGRSDPTSRSWNRLYTRLMAQNGNWLVEVKPWYVVGNTDDNF

WP_055773330_1_pir____________ SVRGSIIANMLQQHDNPFTLYPYESNYILYTYTNHINKEAIQSYNWADNAKNDEVEFQLSLAFPLWRGIVGDNSVLGASYTQKSFWQLSNSAESSPFRETNYEPQLFLAWATDYEFMGWTLREAEYGLNHQSNGRADPTSRSWNRVYARYMAQNGNWQVDLKPWYRIPEsdDNF

WP_012004663_1_pir____________ AVRGSIIANMLQDHDNPFTLYPYDTNYVLYTDTSNINKEAIQSYNWADNARKDEVKFQLSLAFPLWRGIAGDNSVLGASYTQRSWWQLSNHSESSPFRETNYEPQVFLGWATDYSFAGWTLRDVEVGLNHQSNGRSDPTSRSWNRGYARFMAQNGNWQVDLKPWFRFSesDDNF

```
WP_042326129_1_pir_____________AVRGSIIANMLQEHDNPFTLYPYDTNYLIYTNTSDMNKEAIGSYNWSENARKDEVKFQLSLAFPIWRGIAGPNSVLGASYTQKSWWQLSNTEESSPFRETNYEPQLFLGFATDYRVAGWTLRDVEMGYNHDSNGRSDPTSRSWNRLYTRLMAENGNWLVEVKPWYVIGSTDDNP
CNF02030_1_pir_________________AVRGSIIAAMLQDHDNPFLLYPYETNYLLYTYSSNINKEAISSYDWAENARKDEIKFQLSLGFPIWRGIAGENSLLGASYTQRSWWQASNSGESSPFRETNYEPQVFLAWATDYDFAGWTFREIEVGLNHQSNGKADPTSRSWDRAYARVMAQRGDWEIDLKPWYRFSekDDNP
WP_057628331_1_pir_____________WELAKDSKLGLYQLRAYKPVYLLPAFWSSKPNEMPHSPNPNNTVTESEQLDSTELKFQLSFKTKVAENLFGDNGDIWVGYTQSSRWQAYNSELSRPFRETNYEPEVMLTFRNGYSLFGWNGRMTGVSLNHQSNGRSDPLSRSWNRVVMNVGLDRENWAIMLRPWYRIPedDNP
WP_042845834_1_pir_____________PIRGSIISGLLQNYDNPFVLYPYESNYIIYTYTSSINKNAISSYNWGDDALKDEVKFQLSLAFPLWEGIAGDDSILAVSYTQRSWWQLSNKKESSPFRETNYEPQVFVGWLTDYQLGGWTLREIETGFNHESNGRSDPTSRSWNRVYARMMAQNGNWQVDLKPWYRIHesDDNS
WP_004653752_1_pir_____________WELSEESKLGVWNIRAYQPVYLLPVFWTSDKNEFPTSPNPENTVKDKQELTSSEAKFQLSFKTKAWENILGNNGDLWLGYTQSSRWQVYNSEESRPFRETNYEPEASLMFRTNYEILGLNARLLGVTLNHQSNGRSDPLSRSWNRVIFNLGFEKDNFALMLRPWYRVEedDNNP
WP_049610136_1_pir_____________TVRGSIIAAMLQDHDNPFLLYPYETNYLLYTYTSNINKNAISSYDWADNARKDEVKFQLSLGFPIWRGIAGENSLLGASYTQRSWWQASNSGESSPFRETNYEPQVFLAWATDYELAGWNFREVEFGLNHQSNGKADPTSRSWNRAYTRVMAQRGDWEVDLKPWYRLpeKDDNP
WP_026110591_1_pir_____________SVRGSIIANMLQEHDNPFTLYPYDTNYVIYTYTNHINKEAIQSYNWADNAKNDELKFQLSLAFPLWRGIAGDNSVLGASYTQKSFWQVSNSAESSPFRETNYEPQLFLAWATDYEVLGWTLREAEYGLNHQSNGRADPTSRSWNRVYARFMAQRGDWQVEVKPWYRIPenDDNP
WP_039991531_1_pir_____________AVRGSIIANMLQSHDNPFTLYPYDTNYVLYTDTSDLNKEAIETYNWSDKARKDEVKFQLSLAFPLWRGIAGENSVLGASYTQRSWWQLSNSGQSSPFRETNYEPQVFLGWATDYSFAGWTLRDVEIGLNHQSNGRSEPTSRSWNRAYARLMAQNGNWQVDVKPWLRFSesDDNP
EFE96952_1_pir_________________AVRGSIIANMLQSHDNPFTLYPYDTNYVLYTDTSDLNKEAIETYNWSDKARKDEVKFQLSLAFPLWRGIAGENSVLGASYTQRSWWQLSNSGQSSPFRETNYEPQVFLGWATDYSFAGWTLRDVEIGLNHQSNGRSEPTSRSWNRAYARLMAQNGNWQVDVKPWLRFSesDDNP
AGN86610_1_pir_________________AVRGSIIANLLQEHDNPFTLYPYESNYVIYTDTSDLNKEAIRSYNWADNARKDEVKFQLSLAFPLWRGILGPNSVFGGSYTQKSWWQLSNSGESSPFRETNYEPQLFLGFATDYEFAGWTLRDVEFGFNHDSNGRSDPTSRSWNRAYTRLMAQSGNWLVEVKPWYVVGSVDDNP
WP_013577507_1_pir_____________AVRGSIIANMLQEHDNPFTLYPYEQNYLIYTDTSNINKKAIQSYDWADKARHDEVEFQLSLAFPLWRGIAGDNSVLGASYTQKSFWQLSNSGESSPFRETNYEPQLFLAWATDYDFLGWTLREAEYGVSHQSNGRSDPTSRSWNRVYARFMAQNGNWQVDLKPWYRIPEsdDNP
WP_018679243_1_pir_____________WELSEESKLGLWNIRAYQPVYLLPAFWTSNKNEFPSSPNPNNTVTDKQNLSSTEAKFQISLKTKALENILGNNGDVWLGYTQSSHWQVYNAEESRPFRETNYEPEASLMFRTNYEILGLNARLLGVTLNHQSNGRSDPLSRSWNRVIFNLGFEKDNFALMLRPWYRVEEsdNNP
WP_054659171_1_pir_____________WELARDSKLGRFQLRAYKPVYLLPAFWTSKPNVMPRSPNPANSVTQAEELESTELKFQLSFKTKVAENLFGDNGDLWVGYTQSSRWQAYNSEISRPFRETNYEPEVMLAFRNGYSLFGWNGRMSAVSFNHQSNGRSDPLSRSWNRVILNVGLDRENWAVMLRPWYRIPedDNNP
WP_014729809_1_pir_____________AVRGSIIANLLQDHDNPFTLYPYESNYLLYTVTDDLNKEAIKSYDWADNARKDEVKFQLSLAFPFWRGIMGPNSVLGGSYTQKSWWQLSNSGQSSPFRETNYEPQLFLGFATDYELAGWTLRDVEFGYNHQSNGRSDPTSRSWNRLYTRLMAQNGDWLVEVKPWYVVGNTDDNP
EHC79958_1_pir_________________AVRGIIIANMLQEHDNPFTLYPYDTNYLIYTNTSDLNKEAISTYNWSENARKDEVKFQLSLAFPLWRGILGPNSVLGASYTQKSWWQLSNSKEssCFRETNYEPQLFLGFATDYRFAGWTLRDVEMGYNHDSNGRSDPTSRSWNRLYTRLMAENGNWLVEVKPWYVIGSTDDNP
WP_016539950_1_pir_____________WELSEESKLGVWNIRAYQPVYLLPVFWTSDKNEFPSSPNPENTVKDKQELTSSEAKFQLSFKTKAWENIFGNNGDLWLGYTQSSRWQVYNSEESRPFRETNYEPEASLMFRTNYEILGLNARLLGVTLNHQSNGRSDPLSRSWNRVIFNLGFEKDNFALMLRPWYRVEedDNNP
WP_058570356_1_pir_____________WELAKDSKLGTFQLRAYKPVYLLPAFWTSKKNETPSSPNPNDTVTSPESLDSIEAKFQLSFKTKIVENIFGDNGDLWGAYTQSSRWQVYNSEQSRPFRETNYEPELALVFRNNYSLFGWKGRMTGIQLTHQSNGRSDPFSRSWNRAILNVGLDRENWALVLRPWYRIPEndNNP
WP_039005032_1_pir_____________WELAKDSKLGTFQLRAYKPVYLLPAFWTSKKNETPSSPNPNDTVTSPESLDSIEAKFQLSFKTKIVENIFGDNGDLWGAYTQSSRWQVYNSEQSRPFRETNYEPELALVFRNNYSLFGWKGRMTGIQLTHQSNGRSDPFSRSWNRAILNVGLDRENWALVLRPWYRIPEndNNP
WP_032615125_1_pir_____________AVRGSIIANLLEEHDNPFTLYPYDSNYLIYTATSDLNKEAIRTYDWADDSQKDEVKFQLSLAFPLWRGILGPNSILGGSYTQKSWWQLSNSDESSPFRETNYEPQLFLGFATDYSLAGWTLRDVEFGYNHMSNGRSDPTSRSWNRLYTRLMAQNGNWLVEVKPWYVVGNTDDNP
CCK04820_1_pir_________________AVRGSIIANLLQDHDNPFTLYPYESNYLLYTVTDDLNKEAIKSYDWADDARKDEVKFQLSLAFPFWRGIMGPNSVLGGSYTQKSWWQLSNSGQSSPFRETNYEPQLFLGFATDYELAGWTLRDVEFGYNHQSNGRSDPTSRSWNRLYTRLMAQNGDWLVEVKPWYVVGNTDDNP
WP_039329242_1_pir_____________AVKGSIIANLLEKHDNPFVLYPYENNYLLYTYTSDMNKEAISSYNWADKAKNDEVKFQLSLAFPLWRGILGDNSVLAASYTQRSWWQLSNRGASSPFRETNYEPQIFLGWATDYTFAGWTLRDIETGLNHESNGRSDPTSRSWNRVYARLMAQNGNFLAEIKPWYRIPenDDNP
WP_053834267_1_pir_____________WELAKDSKLGTFQLRAYKPVYLLPAFWTSKKNETPSSPNPNNTVTSPESLDSIEAKFQLSFKTKIVENIFGDNGDLWGAYTQSSRWQVYNSEQSRPFRETNYEPELALVFRNNYSLFGWKGRMTGIQLTHQSNGRSDPFSRSWNRAILNVGLDRENWALVLRPWYRIPEsdNNP
WP_061499131_1_pir_____________AVRGSIIANLLQEHDNPFTLYPYESNYVIYTDTSDLNKEAIRSYNWSDNARKDEVKFQLSLAFPLWRGILGPNSVFGGSYTQKSWWQLSNSSESSPFRETNYEPQLFLGFATDYELAGWTLRDVEFGFNHDSNGRSDPTSRSWNRLYTRLMAQQGNWLVEVKPWYVVGSTDDNP
WP_004911364_1_pir_____________WELSEESKLGVWNIRAYQPVYLLPVFWTSDKNEYPESPNPNNTVTDKQNLTSSEAKFQISLKTKAWENIFGNNGDLWLGYTQSSRWQVYNSEESRPFRETNYEPEASLMFRTNYELLGLNARLLGVTLNHQSNGRSDPLSRSWNRVIFNLGFEKDNFALMLRPWYRIEedDNNP
WP_045815823_1_pir_____________AVKGSIIANLLEKHDNPFVLYPYENNYLLYTYTSDMNKEAISSYNWADKAKNDEVKFQLSLAFPLWRGILGDNSVLAASYTQRSWWQLSNRGASSPFRETNYEPQIFLGWATDYTFAGWTLRDIETGLNHESNGRSDPTSRSWNRVYARLMAQNGNFLAEIKPWYRIPenDDNP
WP_012666591_1_pir_____________AVPGSIIANLLEKHDNPFVMYPYESNYLLYTDTSNLNKEAIRSYSWSDKAKHDEVKFQLSLAFPLWRGIAGDSSVLAASYTQKSWWQLSNRGASSPFRESDYEPQIFLGWATDYRFAGWTLRDVETGFNHQSNGRSEPTSRSWNRVYARLMAENGNWLVEVKPWYRLPEsdDNP
WP_051464464_1_pir_____________WELAKDSKLGIFQLRAYKPVYALPAFWTSKPNTLPHSPNPNNTATTPEALDAVEAKFQISFKTKLAEDVFGDNGDVWAGYTQSSRWQAYNSELSRPFRETNYEPEVMLVFRNNYSLFGWKGRMMGVGINHQSNGRSDPLSRSWNRVIFTAGLDRENWAFVVRPWYRISEgdNNP
WP_062791175_1_pir_____________AVRGSIIANMLQDHDNPFTLYPYDTNYVLYTDTSNINKEAIKSYNWAEKARKDEVKYQLSLAFPLWRGILGDNSVLGASYTQRSWWQLSNHGESSPFRETNYEPQVFLGWATDYRFAGWTLRDVEVGLNHQSNGRSDPTSRSWNRGYARFMAQNGNWQVDLKPWFRFSesDDNP
WP_050100968_1_pir_____________TVRGSIIAAMLQDHDNPFLLYPYETNYLLYTYTSDINKNAISSYDWAENAKKDEVKFQLSLGFPIWRGIAGENSLLGASYTQRSWWQASNKDESSPFRETNYEPQLFLAWATDYEFAGWTFREVEFGYNHQSNGKADPTSRSWDRIYTRVMAQRGDLEIDLKPWYRLpeKDDNP
WP_029590749_1_pir_____________AVRGSIIANLLQEHDNPFTLYPYESNYLLYTVTDDLNKEAIQSYNWSDNARKDEVKFQISLAFPLWRGILGENSVLGASYTQRSWWQLSNSGESSPFRETNYEPQLFLGFATDYSLGGWTLRDVEVGYNHHSNGRSDPTSRSWNRAYARLMAQNGNWLVEAKPWYVLGNTDDNP
WP_070927819_1_pir_____________PVRGSIISGLLQNYDNPFVLYPYESNYIIYTYTSSMNKDAIKSYDWGKDALRDEVKFQLSLAFPLWRGIAGDNSVLAASYTQRSWWQLSNKKESSPFRETNYEPQIFIGWATDYQLGGWTLREFETGFNHESNGRSDPTSRSWNRIYARAMAQNGNWQVDLKPWYRLPesDDNK
WP_016677402_1_pir_____________............DHDNPFLLYPYETNYLLYTYTNEINKQAISSYDWAEQANKDEVKFQLSLAFPIWRGIAGDNSLLGASYTQRSWWQASNSEESSPFRETNYEPQLFLAWSTDYELAGWTFREVEFGFNHQSNGKADPTSRSWNRVYTRVMAQRGNLEIDLKPWYRIPekDDNP
WP_023333803_1_pir_____________AVHGSIIANLLQEHDNPFTLYPYDTNYVIYTQTSDLNKEAISSYNWSDNARKDEVKFQLSLAFPFWRGILGPNSVLGASYTQKSWWQLSNSGESSPFRETNYEPQLFLGFATDYEFAGWTLRDVEVGFNHDSNGRSDPTSRSWNRAYTRLMAQNGNWMVEVKPWYVVGETDDNP
WP_066096496_1_pir_____________WELAKDSKLGTFQLRAFKPVYLLPAFWTSRKNTLPSSPNPANTVTTPQVLDSTELKFQLSFKTKVWENIFGDNGDLWAGYTQSSRWQAYNAEDSRPFRETNYEPELMLVFRNNYSFAGWHGRMAAIGLDHQSNGRSDPLSRSWNRVILNFGFDRDNWAFVVRPWYRVPedDNNP
WP_004934270_1_pir_____________AVRGSIIANLLQEHDNPFTLYPYDPNYLLYTDTSDINKEAIKTYNWADKARKDEVKYQLSLAFPLWRGIAGDNSVLGASYTQRSWWQLSNRGESSPFRETNYEPQLFVGWATDYRFAGWTLRDVEIGVNHQSNGRSDPTSRSWNRGYARLMAQNGNWQVDLKPWFRFSEsdDNP
WP_025420597_1_pir_____________AVKGSIIANLLQEHDNPFTLYPYESNYLLYTYTSNINKQAISSYDWANNAKKDEVAFQLSLGFPLWRGIAGDNSLLGASYTQRSWWQLSNSEESSPFRETNYEPQLFIGWATDYDFAGWTLRDVEMGLNHQSNGRSDPTSRSWNRAYTRLMAQNGNWQVEVKPWLRLDDSSDNP
WP_005313536_1_pir_____________WELSEESKLGVWNIRAYQPVYLLPVFWTSDKNEFPSSPNPENTVKDKQELTSSEAKFQLSFKTKAWENIFGNNGDLWLGYTQSSRWQVYNSEESRPFRETNYEPEASLMFRTNYEILGLNARLLGVTLNHQSNGRSDPLSRSWNRVIFNLGFEKDNFALMLRPWYRVEedDNNP
WP_062166069_1_pir_____________WELAKDSKLGLYQLRAYKPVYLLPAFWSSKPNEMPHSPNPNNTVTESEQLDSTELKFQLSFKTKVAENLFGDNGDIWVGYTQSSRWQAYNSELSRPFRETNYEPEVMLTFRNGYSLFGWNGRMTGISLNHQSNGRSDPLSRSWNRVVMNVGLDRDNWAIMLRPWYRIPedDNNP
WP_063863394_1_pir_____________AVKGSIIANMLQEHDNPFTLYPYDTNYVIYTQTSDLNKEAISSYNWSDNARKDEVKFQLSLAFPFWRGILGPNSVLGASYTQKSWWQLSNSGESSPFRETNYEPQLFLGFATDYEFAGWTLRDVEVGYNHDSNGRSDPTSRSWNRIYTRLMAQNGNFMVEVKPWYVVGNTDDNP
WP_067709753_1_pir_____________AVRGSIIANLLEKHDNPFVLYPYESNYIIYTDTSSINKEAIKSYNWANKAKHDEAKFQLSLAFPLWRGIAGDNSVLAASYTQDSWWQISNRGGSSPFRETNYQPQIFIGWATDYSFAGWTLRDVETGLNHESNGRSDPTSRSWNRVYARLMAQNGNWMVEAKPWYRIPEsdDNP
WP_004830448_1_pir_____________WELSQDSKLGTWNIRAHQPVYLLPAFWTSKKNEFPHSPNPQNTVDEDQNLKSMEAKFQISLKTKAVENLFGNNGDLWLGYTQSSRWQVYNSEESRPFRETNYEPEASLMFRTNYNLLGLNGRLLGLTLNHQSNGRSDPLSRSWNRVILNLGFEKDNFALMVRPWYRIEedDNNP
WP_061798820_1_pir_____________AVRGSIIANMLQDHDNPFTLYPYETNYVLYTDTSDLNKEAISSYNWADKARKDEVKFQLSLAFPLWRGIVGDNSVLGASYTQRSWWQLSNHGESSPFRETNYEPQVFLGWATDYRFAGWTLRDVEVGLNHQSNGRSEPTSRSWNRGYARFMAQNGNWQVDLKPWFRFSesDDNP
WP_048618851_1_pir_____________TVRGSIIAAMLQDHDNPFLLYPYETNYLLYTYTSDINKDAISSYDWAQQAKKDEVKFQLSLGFPIWRGIAGENSLLGASYTQRSWWQASNSDESSPFRETNYEPQLFLAWATDYEVAGWTFREVEFGYNHQSNGKADPTSRSWDRVYTRVMAQRGDLEIDLKPWYRLPekDDNP
WP_023479589_1_pir_____________AVRGSIIANMLQEHDNPFTLYPYESNYVIYTDTSDLNKEAIRSYNWADNARKDEVKFQISLAFPFWRGILGPNSVLGGSYTQRSWWQLSNSGESSPFRETNYEPQLFLGFATDYKFAGWTLRDVEVGFNHDSNGRSDPTSRSWNRAYTRLMAQNGNWLVEVKPWYVVGSTDDNP
WP_074865257_1_pir_____________WELAKDSKLGVFNFRAYKPVYLLPVFWNNDPNTLPHSPNPRNTVTEAQSLDSVEAKFQISFKTKAAENLFGDNGDLWMGYTQSSRWQVYNGENSRPFRETNYEPEVLLVFRNNYRIGDWKGRMFAVGLNHQSNGRADPLSRSWNRVMFNIGLDREDWALMLRPWYRigNDDDNP
WP_016539001_1_pir_____________AVRGSVIANLLQEHDNPFTLYPYDTNYLLYTWTSDLNKEAISSYDWASNARKDEVKFQLSLAFPLWRGILGDNSVLGASYTQKSWWQLSNRNESSPFRETNYEPQLFLGFATDASFAGWTLRDVEFGYNHDSNGRSDPTSRGWNRLYTRLMAQNGDWQVEVKPWYVVGSVDDNP
WP_034938864_1_pir_____________AVRGSIIANLLEKHDNPFVLYPYESNYILYTDTSNINKEAIQSYNWADKAKHDEVKFKLSLAFPLWRGILGDNSVLAASYTQDSWWQLSNRGGSSPFRETDYQPQIFLGWATDYSFAGWTLRDVEMGLNHQSNGRSEPTSRSWNRVYARFMAQNGNWLVEAKPWYRLPEsdDNP
WP_033737373_1_pir_____________QVRGSIIANLLEQHDNPFVLYPYENNYLLYTYTSDINKEAISSYNWAKNARKDEVKFQLSLAFPLWRGIVGDNSVLAASYTQRSWWQLSNKKESSPFRETNYEPQIFLGWATDYSFAGWTLRDVEVGVNHQSNGRSDPTSRSWNRVYTRLMAQNGNFLAEIKPWFRISEsdDNP
WP_057640251_1_pir_____________WELARDSKLGRFQLRAYKPVYLLPAFWSSKPNEMPHSPNPNNTVTQPEKLDDTELKFQLSFKTKVAENLFGDNGDVWVGYTQSSRWQAYNSELSRPFRETNYEPEAMLVFRNGYSLLGWNGRMTGISLNHQSNGRSDPLSRSWNRVILSVGLDRENWALVLRPWYRVPedDNNP
WP_008914037_1_pir_____________PVRGSIISGLLQNYDNPFVLYPYESNYIIYTYSSSINKEAIKSYDWGKDALRDEVKFQLSLAFPLWRGIAGDNSLLAASYTQRSWWQLSNKKESAPFRETNYEPQVFIGWATDYQFAGWTLRDIETGFNHESNGRSDPTSRSWNRIYARAMAQNGNWQVDLKPWYRIHesDDNP
WP_034898091_1_pir_____________AVNGSIIANLLEKHDNPFVLYPYESNYVLYTDTSNINKEAIQSYNWADSAKHDEVKFQLSLAFPLWRGILGDNSVLAASYTQGSWWQLSNRGGSSPFRETDYQPQIFLGWATDYTFAGWTLRDVETGLNHQSNGRSEPTSRSWNRVYARLMAQNGNWMVEAKPWYRIPEsdDNP
WP_020882880_1_pir_____________AVQGSIIANLLQEHDNPFTLYPYDTNYVIYTQTSDLNKEAISSYNWSDNARKDEVKFQLSLAFPFWRGILGPNSVLGASYTQKSWWQLSNSGESSPFRETNYEPQLFLGFATDYEFAGWTLRDIEVGFNHDSNGRSDPTSRSWNRAYTRLMAQNGNWMAEVKPWFVVGDTDDNP
WP_038015390_1_pir_____________AVRGSIIANLLEKHDNAFLLYPYESNYVLYTDTSNMNTSAISSYSWADKAKKDELKFQLSLAFPLWRGIAGDNSVLAMSYTQRSWWQISNTRASSPFRETNYEPQLFIGWATDYSLGGWTLRDVEAGFNHQSNGRSDPTSRSWNRVYARLMAKNGNMLAEIKPWYRIPesDDNP
WP_049839564_1_pir_____________AVRGSVIANLLQEHDNPFTLYPYDSNYVLYTWTSDLNKEAINTYNWADNARKDEVKFQLSLAFPFWRGILGPNSVLGASYTQKSWWQLSNSGESSPFRETNYEPQLFLGFATDYRLGDWSLRDVEVGFNHDSNGRSDPTSRSWNRAYTRLMAQNGNWLVEVKPWYVIGSTDDNP
WP_012847010_1_pir_____________SDNEGVIANMLRHYDNPFTLYPYSTNYVLYTQTSDINKEAISSYDWGDQAKKNEIKFQISLAFPIWRGIAGDNSVLGAAYTQQSWWQAFNRSESSPFRETNYEPRLFLGWATDYPLfgWVLRDIEAGGVHQSNGRSDPTSRSWNRVYTRFLAQNGNFQVQLMPWYRIPekDDNP
AGH72233_1_pir_________________SDNEGVIANMLRHYDNPFTLYPYSTNYVLYTQTSDINKEAISSYDWGDQAKKNEIKFQISLAFPIWRGIAGDNSVLGAAYTQQSWWQAFNRSESSPFRETNYEPRLFLGWATDYPLfgWVLRDIEAGGVHQSNGRSDPTSRSWNRVYTRFLAQNGNFQVQLMPWYRIPekDDNP
WP_031523725_1_pir_____________AVRGSIIANLLQEHDNPFTLYPYESNYLLYTVTSDLNKEAIGSYDWSDNARKDEVKFQLSLAFPFWRGIMGPNSVLGASYTQKSWWQLSNSGESSPFRETNYEPQLFLGFATDYRLAGWTLRDVEFGYNHQSNGRSDPTSRSWNRLYTRLMAQNGNWLVEVKPWYVMGSTDDNP
WP_004981834_1_pir_____________WELSPESKLGTWNIRAYQPVYLMPGFWTTDKNEYPQSPNPTNTVDEDQNLKSMEAKFQLSLKTKAVENLFGDNGDVWLGYTQSSRWQLYNSDESRPFRETNYEPEASLVFRTNYELLGLNGRLLGLTFNHQSNGRSDPLSRSWNRVMLNVGFERDNFALMLRPWYRLDedDNNP
CCJ90903_1_pir_________________AVRGSIIANLLQDHDNPFTLYPYESNYLLYTVTDDLNKEAIKSYDWTDNARKDEVKFQLSLAFPFWRGIMGPNSVLGASYTQKSWWQLSNSGQSSPFRETNYEPQLFLGFATDYDLAGWTLRDVEFGYNHQSNGRSDPTSRSWNRLYTRLMAQNGDWLVEVKPWYVIGDTDDNP
WP_045260988_1_pir_____________AVKGSIIANMLQEHDNPFTLYPYDTNYVIYTQTSDLNKEAISSYNWSDNARKDEVKFQLSLAFPFWRGILGPNSVLGASYTQKSWWQLSNSGESSPFRETNYEPQLFLGFATDYEFAGWTLRDVEVGFNHDSNGRSDPTSRSWNRAYTRLMAQNGNWMVEVKPWYVIGSTDDNP
WP_057645128_1_pir_____________WELAKDSKLGTFQLRAYKPVYLLPAFWTSKVNETPSSPNPNNTVTDPQPLDSTELKFQLSFKTKVVEDVFHDNGDIWVGYTQSSRWQSYNAEESRPFRETNYEPEAMLVFRNNYGLFGWRGRMTGIALTHQSNGRSDPLSRSWNRVILNFGLDRENWALMLRPWFRIKedDNNP
WP_004822164_1_pir_____________WELSQDSKLGTWNIRANQPVYLLPAFWTSKKNEFPQSPNPQNTVDEDQNLKSMEAKFQISLKTKAAENIFGNNGDVWVGYTQSSRWQVYNSEESRPFRETNYEPEASLIFRTNYNLLGLNGRLLGLTLNHQSNGRSDPLSRSWNRVILNLGFEKDNFALMVRPWYRIEedDNNP
WP_006015922_1_pir_____________PPKKPKKVNILSDFAKKYLDIMEYRGTYFMPFYHSFTPIYQWYFPSINRYQSTEFKFQVSFRVPVIRHFLFTTGTLYLAYTQTNWFQIYNNPQSAPMRMVNYMPELIYVYPLNIPFfmGHLSEFWIGWQHISNGIGGRqvSAGQRPVFKLTWQKGGLKIRVAYWPYIPYNQSNP
WP_044435077_1_pir_____________WELSEKAKLGTWNIRAFKPVYLMPVFWTTKKNEFPSSPNPNNTVTENQNLDSLESKFQLSLKTKAWENIIGNNGDLWLGYTQSSRWQVYNGDESRPFRETNYEPEASLMFRTNYKVFGLDGRLLGVTLNHQSNGRSDPLSRSWNRVIFNVGFEKDNFALMLRPWVRIQEddNNP
WP_010426300_1_pir_____________AVRGSIIANMLQEHENPFTLYPYDTNYVIYTQTSDLNKEAISSYNWSDNARKDEVKFQLSLAFPFWRGILGPNSVLGASYTQKSWWQLSNSGESSPFRETNYEPQLFLGFATDYEFAGWTLRDVEVGFNHDSNGRSDPTSRSWNRAYTRLMAQNGNWMVEVKPWYVVGSTDDNP
WP_020824779_1_pir_____________AVRGSIIANMLQDHDNPFTLYPYETNYVLYTDTSNINKEAIQSYNWSDNARKDEVKFQLSLAFPLWRGIAGDNSVLGASYTQRSWWQLSNHGESSPFRETNYEPQVFLGWATDYRFAGWTLRDVEVGLNHQSNGRSDPTSRSWNRGYARFMAQNGNWQVDLKPWFRFSesDDNP
WP_014833705_1_pir_____________AVRGSIIANLLQEHDNPFTLYPYDTNYVLYTWTSDLNKEAIRSYDWAENARKDEVKYQLSLAFPFWRGILGPNSVLGASYTQKSWWQLSNSGESSPFRETNYEPQLFLGFATDYEFAGWTLRDVEVGFNHDSNGRSDPTSRSWNRAYTRLMAQNGNLMVEVKPWYVVGSTDDNP
WP_038865789_1_pir_____________AVRGSIIANLLQEHDNPFTLYPYESNYLLYTVTDDLNKEAIKSYDWTDNARKDEVKFQLSLAFPFWRGIMGPNSVLGASYTQKSWWQLSNSGQSSPFRETNYEPQLFLGFATDYQLAGWTLRDVEFGYNHQSNGRSDPTSRSWNRLYTRLMAQNENWLVEVKPWYVIGDTDDNP
WP_025263770_1_pir_____________AVRGSIIANLLQEHDNPFTLYPYESNYVIYTDTSDLNKEAIRSYNWADNARKDEVKFQLSLAFPFWRGILGPNSVFGGSYTQKSWWQLSNSGESSPFRETNYEPQLFLGFATDYEFAGWTLRDVEFGFNHDSNGRSDPTSRSWNRAYTRLMAQSGNWLVEVKPWYVVGSVDDNP
WP_067658486_1_pir_____________WELAADSKLGTWNIRAHEPVYLLPGFWTSDKNEQPESPNEKNSLPADQNLKSMEAKFQLSLKTKALENLIGDNGDVWLGYTQSSRWQVYNEEESRPFRETNYEPEASLVFRTNYELLGLNGRLLGLTFNHQSNGRSDPLSRSWNRVMLNVGLERGNFAMMIRPWYRIEedDNNP
WP_038252972_1_pir_____________AVRGSIIANMLQEHDNPFVLYPYESNYVIYTWTSDLNKEAIRSYDWAEHARKDEVKFQLSLAFPLWRGILGDNSVLGASYTQKSWWQLSNSKESAPFRETNYEPQLFLGFATDYQFAGWTLRDVEFGYNHDSNGRADPTSRSWNRLYTRLMAQNGNWLVEVKPWMVIGNTTDNP
WP_072571107_1_pir_____________AVRGSIIANLLQEHDNPFTLYPYESNYAIYTATSDLNKEAIETYNWAKNSRKDEVKFQLSLAFPFWRGIMGPNSVLGASYTQKSWWQLSNSGESSPFRETNYEPQLFLGFATDYELAGWTLRDVEFGFNHDSNGRSDPTSRSWNRVYTRLMAQNSNWLVEVKPWYVVGDTDDNP
WP_009127855_1_pir_____________AVRGSIIANLLEKHDNPFVLYPYENNYLLYTYTTDMNKEAISSYNWADKAKNDEVKFQLSLAFPLWRGILGDNSVLAASYTQRSWWQLSNRGASSPFRETDYEPQIFLGWATDYTFAGWTLRDVETGLVHQSNGRSEPTSRSWNRVYARLMAENGNLLAEIKPWYRLPEsdDNP
```

```
WP_043955844_1_pir_____________AVRGSIIANLLQEHDNPFTLYPYDTNYVIYTDTSDLNKEAIRSYNWSDNARKDEVKFQLSLAFPLWRGILGPNSVFGASYTQKSWWQLSNSAESSPFRETNYEPQLFLGFATDYEFAGWTLRDVEMGFNHDSNGRSDPTSRSWNRIYTRLMAQNGNWMVEVKPWYVVGSTDDN
CCJ87254_1_pir_________________AVRGSIIANLLQEHDNPFTLYPYESNYVLYTVTDDLNKEAIKSYDWADNARKDEVKFQLSLAFPFWRGIVGPNSVLGASYTQKSWWQLSNSGQSSPFRETNYEPQLFLGFATDYQLAGWTLRDVEVGFNHQSNGRSDPTSRSWNRAYARLMAQNDNWLVEVKPWYVLGNTDDN
WP_050556254_1_pir_____________AVRGSIIANLLQEHDNPFTLYPYESNYVLYTVTDDLNKEAIKSYDWADNARKDEVKFQLSLAFPFWRGIVGPNSVLGASYTQKSWWQLSNSGQSSPFRETNYEPQLFLGFATDYQLAGWTLRDVEVGFNHQSNGRSDPTSRSWNRAYARLMAQNDNWLVEVKPWYVLGNTDDN
WP_007717116_1_pir_____________AVRGSIIANLLQEHDNPFTLYPYESNYVLYTVTDDLNKEAIKSYDWADNARKDEVKFQLSLAFPFWRGIVGPNSVLGASYTQKSWWQLSNSGQSSPFRETNYEPQLFLGFATDYQLAGWTLRDVEVGFNHQSNGRSDPTSRSWNRAYARLMAQNDNWLVEVKPWYVLGNTDDN
WP_034588872_1_pir_____________WELSPESKLGTWNIRSYQPVYVLPGFWTSKKNEFPSSPNERNTVTEDQNLKSMESKFQLSLKTKAVENIFGDNGDLWVAYTQSSRWQVYNSEESRPFRETNYEPEVSLVFRTNYDLFGLDWRMLGLTLNHQSNGRSDPLSRSWNRVMLNLGFERNNFALMLRPWYRFEedDNN
WP_064718574_1_pir_____________LVQGSIISGLLQEYDSPFVLYPYESNYIIYTKTSDMNKEAIASYDWGDKAKKDEVKFQLSLAFPLWRGIAGENSVLAASYTQRSWWQLSNKKESAPFRETNYEPQLFLGWVTDYRFAGWTLREIETGFNHESNGRSDPTSRSWNRVYARFMAQKGNFQLDLKPWYRFSEsdDN
WP_037061332_1_pir_____________WELAKDSKLGIFQLRAYKPVYALPAFWTSKPNTLPHSPNPNNTATTPEALDAVEAKFQISFKTKLAEDVFGDNGDVWAGYTQSSRWQAYNSGISRPFRETNYEPEVMLVFRNNYSLFGWKGRMMGVGINHQSNGRSDPLSRSWNRVIFTAGLDRENWAFVVRPWYRISEgdNN
WP_013890942_1_pir_____________PPRKPKKVNILSDFAKKYLDIMEYRGTYFMPFYHSFTPIYQWYFPSINRYQSTEFKFQVSFRVPVIRHFLFTTGTLYLAYTQTNWFQIYNNPQSAPMRMVNYMPELIYVYPLNIPFfmGRLSEFWIGWQHISNGIGGRqvSAGQRPVFKLTWQKGGLKIRVAYWPYIPYNQSN
WP_013890942_1_pir_____________PPRKPKKVNILSDFAKKYLDIMEYRGTYFMPFYHSFTPIYQWYFPSINRYQSTEFKFQVSFRVPVIRHFLFTTGTLYLAYTQTNWFQIYNNPQSAPMRMVNYMPELIYVYPLNIPFfmGRLSEFWIGWQHISNGIGGRqvSAGQRPVFKLTWQKGGLKIRVAYWPYIPYNQSN
WP_057921372_1_pir_____________WELAKDSKLGLFNFRAYKPVYLLPTFWNSDPNELPHSPNPRNTVTEPQSLQSVEAKFQISFKTKAVENLFGDNGDVWLGYTQSSRWQVYNGDNSRPFRETNYEPEAMLVFRNNYHIGGWSGRMAAIGLNHQSNGRADPLSRSWNRVIGQVGLDRENWSILARPWWRigNDDDN
WP_056108275_1_pir_____________WELAKDSKLGLFNFRAYKPVYLLPAFWNSDPNVLPHSPNPRNTVTEPQSLQSVEAKFQISFKTKAVENLFGDNGDVWLGYTQSSRWQVYNSDNSRPFRETNYEPEAMLVFRNNYHIGGWSGRMAAIGLNHQSNGRADPLSRSWNRVIGQVGLDRENWSIVARPWWRigNDDDN
EGT71014_1_pir_________________AVRGSIIANMLQEHDNPFTLYPYDTNYLIYTQTSDLNKEAIASYDWAENARKDEVKFQLSLAFPLWRGILGPNSVLGASYTQKSWWQLSNSEESSPFRETNYEPQLFLGFATDYRFAGWTLRDVEMGYNHDSNGRSDPTSRSWNRLYTRLMAENGNWLVEVKPWYVVGNTDDN
SFZ71754_1_pir_________________PPKKPKKVNILSDFAKKYLDIMEYRGTYFMPFYHSFTPIYQWYFPSINRYQSTEFKFQVSFRVPVIRHFLFTTGTLYLAYTQTNWFQIYNNPQSAPMRMVNYMPELIYVYPLNIPFfmGRLSEFWIGWQHISNGIGGRqvSAGQRPVFKLTWQKGGLKIRVAYWPYIPYNQSN
WP_066749822_1_pir_____________LVQGSIISGLLQKYDSPFVLYPYESNYILYTDTSDMNKEAIQSYDWGNKAKKDEVKFQISLAFPLWRGIAGENSVLAASYTQRSWWQFSNKKESSPFRETNYEPQIFLGWATDYQFAGWTLREIETGLNHESNGRSDPTSRSWNRVYARFMAQKGNFQLDLKPWYRFNEsdDN
AIJ08337_1_pir_________________SDNEGVIANMLRHYDNPFTLYPYSTNYVLYTQTSDINKEAISSYDWGDQAKKNEIKFQISLAFPIWRGIAGDNSVLGAAYTQQSWWQAFNRSESSPFRETNYEPRLFLGWATDYPLfgWVLRDIEAGGVHQSNGRSDPTSRSWNRVYTRFLAQNGNFQVQLMPWYRIPekDDN
WP_040078115_1_pir_____________AVRGSIIANLLQEHDNPFTLYPYDTNYLLYTTTSDLNKEAIKTYSWSDNARKDEVKFQLSLAFPFWRGILGPNSVLGASYTQKSWWQLSNSGESSPFRETNYEPQLFLGFATDYEFAGWTLRDVEFGYNHDSNGRSDPTSRSWNRLYTRLAAQNGNWLVEVKPWYVVGDTDDN
WP_007671108_1_pir_____________AVRGSIIANLLQEHDNPFTLYPYESNYLLYTVTDDLNKEAIKSYDWTDNARKDEVKFQLSLAFPFWRGILGPNSVLGGSYTQKSWWQLSNSGQSSPFRETNYEPQLFLGFATDYELAGWTLRDVEFGYNHQSNGRSDPTSRSWNRLYTRLMAQNDNWLMEVKPWYVIGDTDDN
WP_028018183_1_pir_____________AVHGSIIANLLQEHDNPFTLYPYDTNYVIYTQTSDLNKEAISSYNWADNARKDEVKYQLSLAFPFWRGILGPNSVFGASYTQKSWWQLSNSGESSPFRETNYEPQLFLGFATDYEFAGWTLRDVEVGFNHDSNGRSDPTSRSWNRAYTRLMAQNGNLMVEVKPWYVVGSTDDN
```
