# Supplementary material for: Outer membrane phospholipase A’s roles in Helicobacter pylori acid adaptation
Source: Gut Pathog. 2017 Jun 12;9:36. doi: 10.1186/s13099-017-0184-y (PMC5469174; doi:10.1186/s13099-017-0184-y)
Supplement: Supplementary file 3 — Additional file 3. Homology modelling results. [file 13099_2017_184_MOESM3_ESM.docx]

# **Additional File 3: Homology Modelling**

**Multiple sequence alignment (MSA)**

PDB currently only holds OMPLA structures derived from *E. coli*. PDB ID 1QD5 was used as the monomeric template, and 1QD6 as the template when a dimerized structure was needed. The structural alignment between *E. coli* structure and *H. pylori* model has an RMSD of 0.043 Angstrom over 254 aligned residues with 27.56% sequence identity (using Mustang Pairwise Motif Aligner).

**Preliminary model**

This model was simulated for 100 ns in a POPC membrane (at the Computational Biomolecular Dynamics Group, Max Planck Institute for Biophysical Chemistry in Germany) [4]. Comparing the first simulated model (based on about 100 sequences) with the final model (based on more than 600 sequences) yields 6.6 Å RMSD, yet most variation was observed in the loop region. Furthermore, the residues aligned to construct the final model do seem more closed which would stabilize the structure even more than initially observed (yields a closer RMSD over a larger number of residues to the template).

**Polar core residues**

Polar residues of HpOMPLA in stick-view mode coloured according to side chain properties in Figure 1. These residues are compared to EcOMPLA in Figure 2.

*
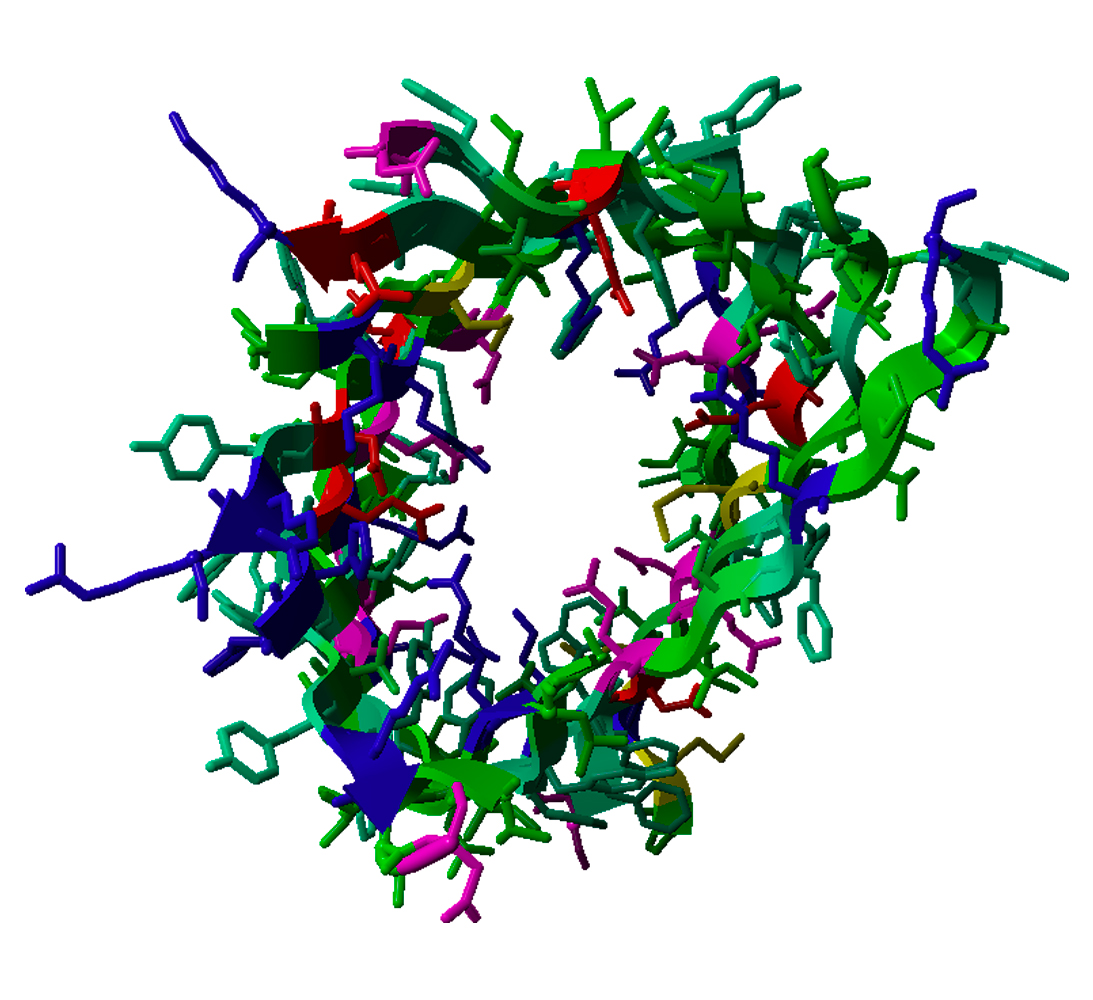
*

**Figure 1: Polar core residues of HpOMPLA.** Structure is visualized using YASARA-WHATIF twinset. Positively charged residues (Arg, Lys, and His) are coloured blue, negatively charged residues (Glue, and Asp) are coloured red, polar residues (Asn, and Gln) are coloured purple, hydrophobic residues (Gly, Ala, Val, Leu, and Pro) are coloured grass green, alcoholic residues (Thr and Ser) are coloured light green, aromatic residues (Phe, Tyr, and Trp) are coloured light blue-green, and sulphur containing residues (Cys, Met) are coloured yellow.

The *H. pylori* OMPLA pore size was estimated to be approximately 4 Å (Figure 3; slightly larger than the 3.5 Å estimated pore size of *E. coli* OMPLA). These estimations are based on the calculations of spherical probes (using the WHAT IF software, see Figure B3A), but OMPLA structures and models have elliptic shaped pores that could allow larger substrates than estimated. PoreWalker characterized the channel and estimated the optimal pore axis for the OMPLA channel, see Figure 3.


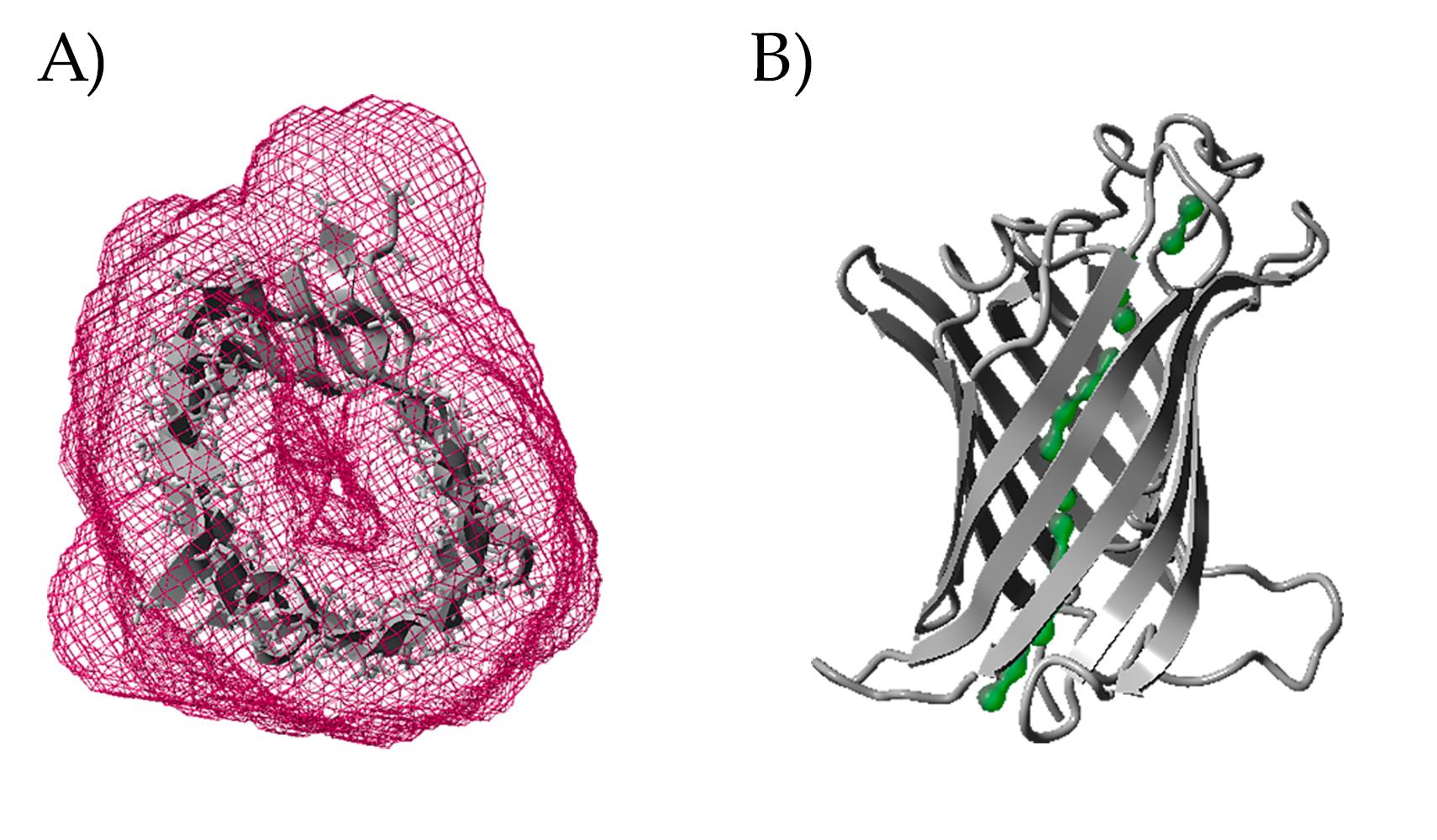


**Figure 3: OMPLA pore function prediction**. A) YASARA’s visualization of the WHAT IF calculated excluded volume around the poly-alanine mutated *H. pylori* OMPLA. The probe radius P is 4 Angstrom. B) PoreWalker predicted molecule (in green) path for *H. pylori* OMPLA.

***
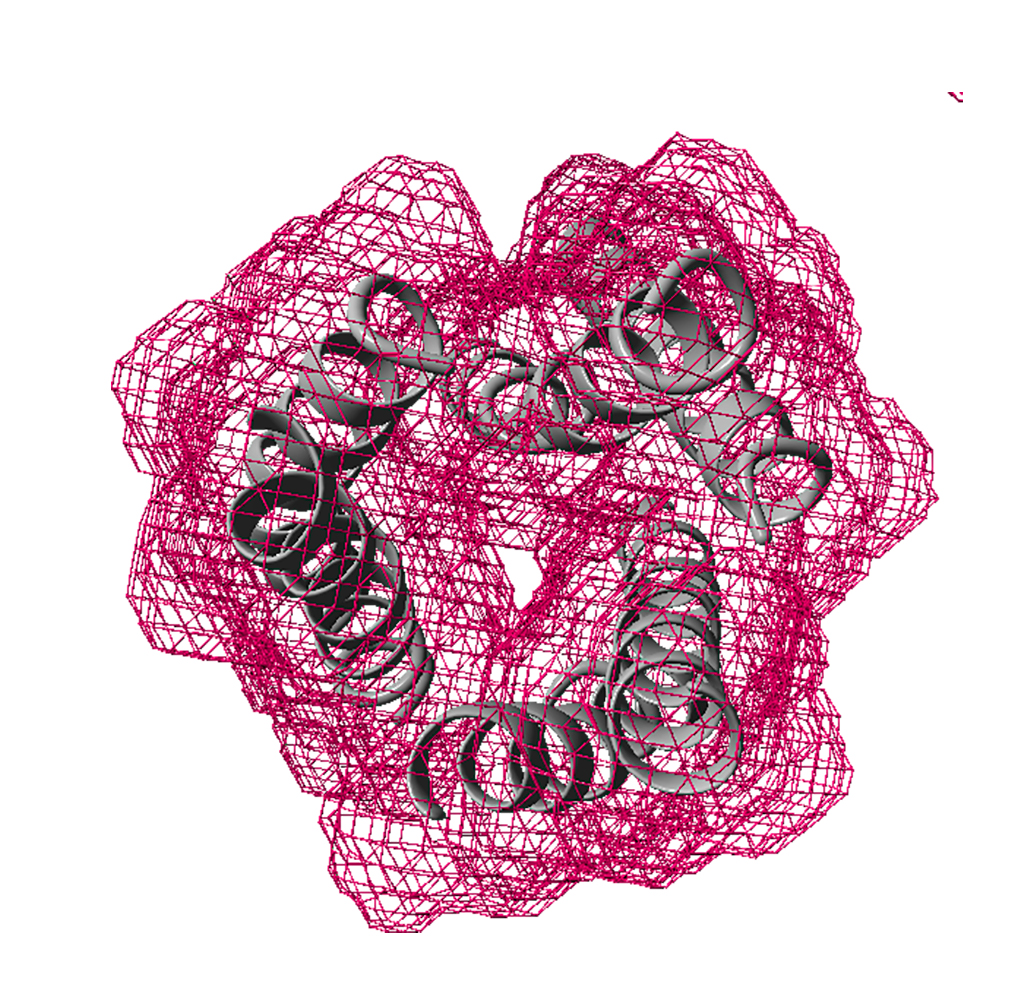
***

**Figure 4: HpUreI pore.** YASARA’s visualization of the WHAT IF calculated excluded volume around the poly-alanine mutated *H. pylori* UreI core, HpUreI. The probe radius P is 1.5Angstrom. This is top-view of the protein, see Figure 2 for the complete hexamer HpUreI structure.

A polar core is observed in *H. pylori* OMPLA structure model (Figure 4) containing more negatively charged residues as compared to the *E. coli* OMPLA structure, see Figure 2. There are many positively charged residues found at the top and bottom of the barrel core (coloured blue in Figure 5), but the majority of these are facing the periplasm.

*
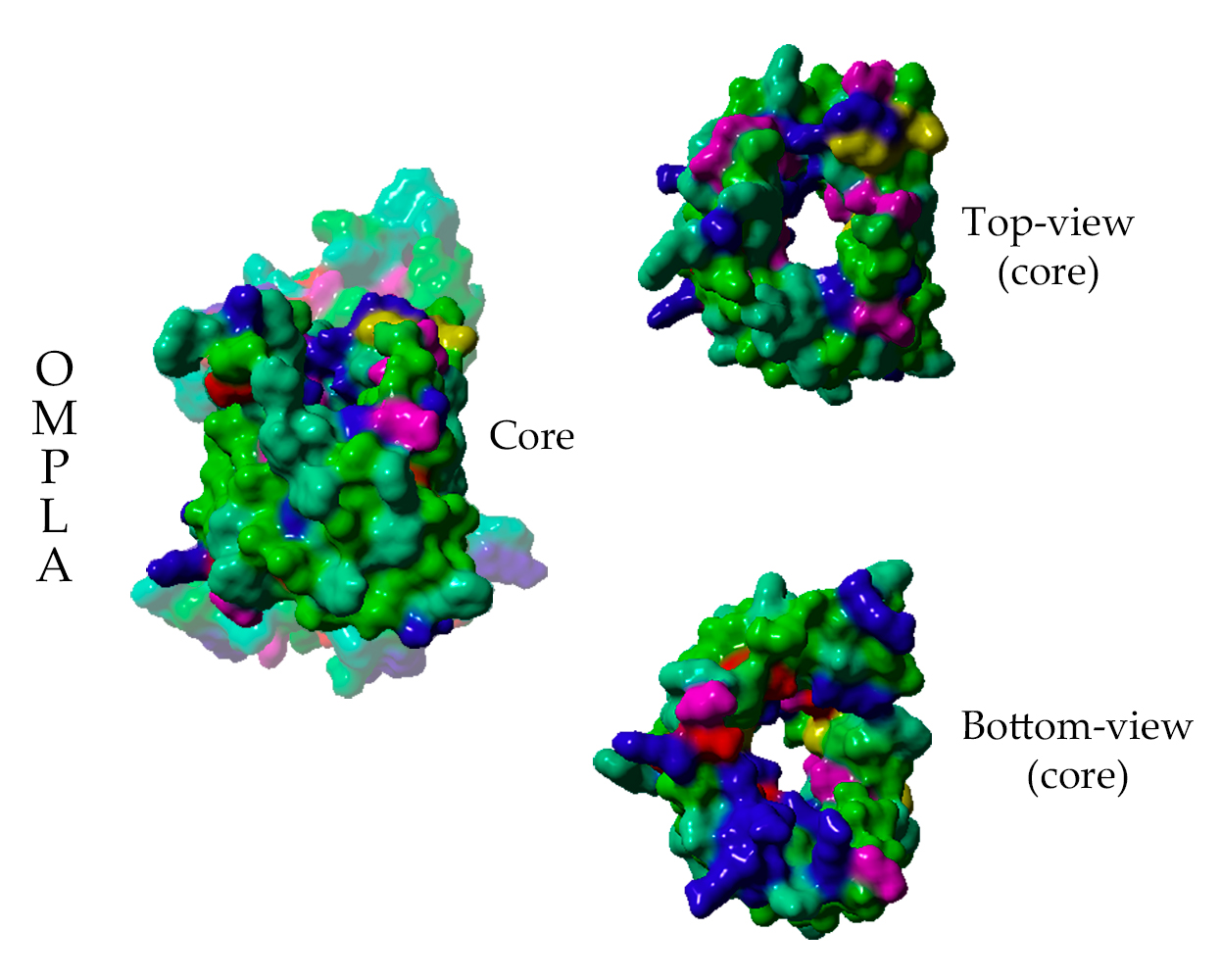
*

**Figure 5: Polar core.** The core residues of *H. pylori* OMPLA model core highlighted with molecular surface using YASARA-WHAT IF twinset. OMPLA residues are coloured according to the amino acid properties, as described in Figure 1.
