# Supplementary material for: Outer membrane phospholipase A’s roles in Helicobacter pylori acid adaptation
Source: Gut Pathog. 2017 Jun 12;9:36. doi: 10.1186/s13099-017-0184-y (PMC5469174; doi:10.1186/s13099-017-0184-y)
Supplement: Supplementary file 5 — Additional file 5. H. pylori OMPLA variants survival in acidic environment. [file 13099_2017_184_MOESM5_ESM.docx]

# **Additional File 5: Detailed results of *H. pylori* OMPLA variants survival in acidic environment**

After screening of *H. pylori* isolates for isogenic variants with high and low OMPLA activity, five strains were detected, see Figure 1 (and Table 1 in the article). Five clinical isolates were examined for spontaneous colony variants showing altered phospholipase A activity by thin-layer chromatography (TLC) of isolated phospholipids as previously described [84] (see Figure 1A). Isogenicity of the five selected variant pairs were confirmed by amplified fragment length polymorphism (AFLP) [32] (see Figure 1B). All variants were confirmed to be *cagA* positive by PCR (using the methodology described by Tannæs *et al.* [1]).


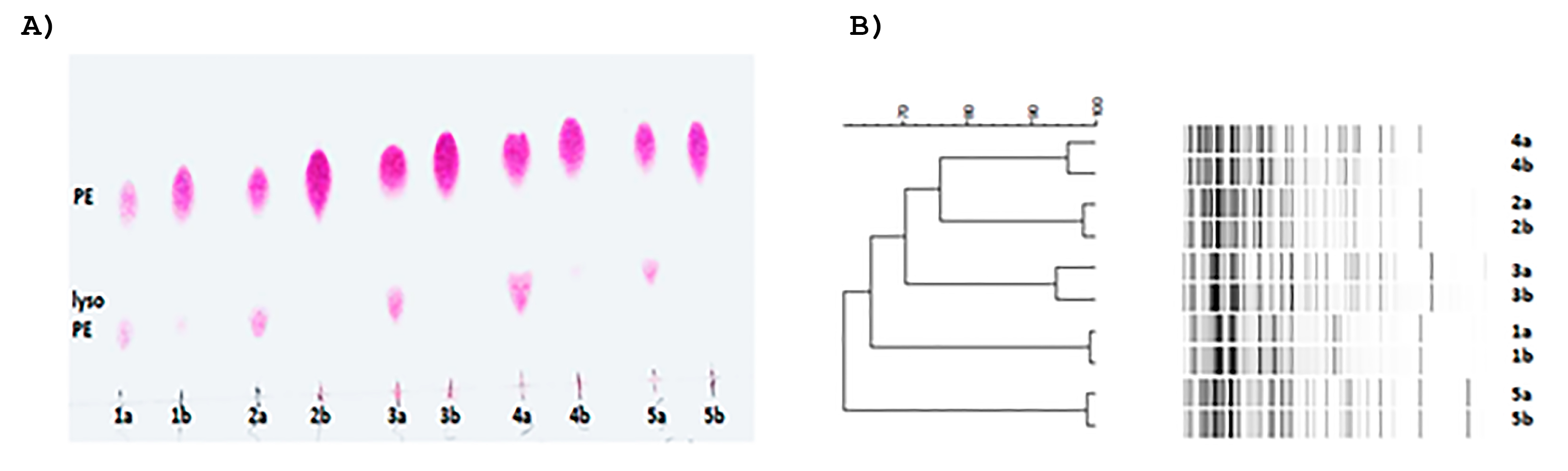


**Figure 1: Characterization of *H. pylori* isolates**. A) Phenotypic characterization: TLC of phospholipids (PE) and lysophospholipids (lysoPE) indicating the variation in OMPLA activity; B) Genotypic characterization: AFLP confirming that the variants originate from 5 single clones. The nomenclature used in both figures: 1, euBL; 2, euAP; 3, euBF; 4, euBZ; 5, euBB; a, OMPLA_ON_; and b, OMPLA_OFF_.

The ability to survive at pH 3.5 varied between the isolates, see Figure 2. As expected the truncated OMPLA variants did not survive the low pH conditions to any significant extent [2]. This was also found to be the case for the missense mutants with a complete, but modified, OMPLA sequence.


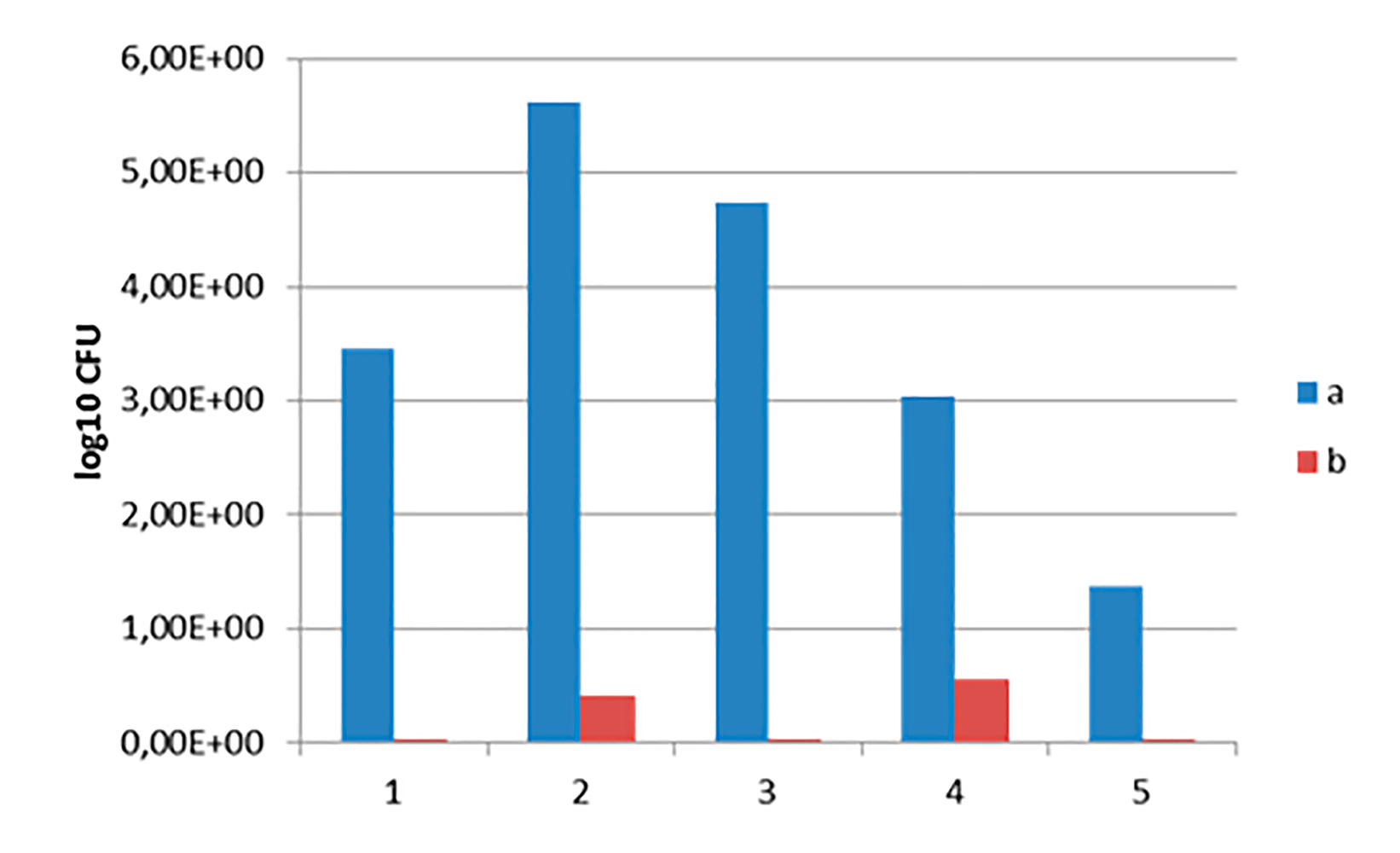


**Figure 2: Survival at pH 3.5.** Survival of isogenic OMPLA variants at pH=3.5. *H. pylori* survival of the different strains (10^8^ CFU) after one passage on blood agar plates at pH 3.5. Numbers on the x-axis corresponds to the five clones 1: euBL; 2: euAP; 3: euBF; 4: euBZ; 5: euBB, where OMPLA_ON_ is highlighted as blue, and OMPLA_OFF_ strains hare marked as red b.

**References**

1. Tannaes T, Bukholm IK, Bukholm G. High relative content of lysophospholipids of *Helicobacter pylori* mediates increased risk for ulcer disease. FEMS Immunol Med Microbiol. 2005; 44:17-23.

2. Tannaes T, Dekker N, Bukholm G, Bijlsma JJ, Appelmelk BJ. Phase variation in the *Helicobacter pylori* phospholipase A gene and its role in acid adaptation. Infect Immun. 2001; 69:7334-7340.
